# Diastereoselective Synthesis of Novel Spiro-Phosphacoumarins and Evaluation of Their Anti-Cancer Activity

**DOI:** 10.3390/ijms232214348

**Published:** 2022-11-18

**Authors:** Valeriia V. Sennikova, Alena V. Zalaltdinova, Yulia M. Sadykova, Ayrat R. Khamatgalimov, Almir S. Gazizov, Alexandra D. Voloshina, Anna P. Lyubina, Syumbelya K. Amerhanova, Julia K. Voronina, Elena A. Chugunova, Nurbol O. Appazov, Alexander R. Burilov, Michail A. Pudovik

**Affiliations:** 1 Arbuzov Institute of Organic and Physical Chemistry, FRC Kazan Scientific Center, Russian Academy of Sciences, Arbuzova str. 8, 420088 Kazan, Russia; 2 N.S. Kurnakov Institute of General and Inorganic Chemistry, Russian Academy of Sciences, Leninsky Ave. 31, 119991 Moscow, Russia; 3 Korkyt Ata Kyzylorda State University, 29A Aiteke Bi St., Kyzylorda 120014, Kazakhstan

**Keywords:** phosphacoumarin, azomethine ylide, cycloaddition, anti-cancer, cytotoxicity, quantum chemistry

## Abstract

Herein we present the regio- and diastereoselective synthesis of novel pyrrolidine-fused spiro-dihydrophosphacoumarins via intermolecular [3 + 2] cycloaddition reaction. The presented approach is complementary to existing ones and provides an easy entry to the otherwise inaccessible derivatives. Additionally, the unprecedented pathway of the reaction of 4-hydroxycoumarin with azomethine ylides is described. The anti-cancer activity of the obtained compounds was tested in vitro, the most potent compound being 2.6-fold more active against the HuTu 80 cell line than the reference 5-fluorouracil, with a selectivity index > 32.

## 1. Introduction

Coumarins and dihydrocoumarins are ubiquitous in nature and have attracted considerable attention due to their biological properties [1,2,3]. Among known activities of coumarins, their anti-cancer properties [4,5] have gained an increasing interest and represent an emerging area of research, as indicated by recent reviews [6,7]. A number of substituted coumarins have been synthesized and tested in attempts to enhance their activity and pharmacological properties. The phosphorus-containing analogues of coumarins, the phosphacoumarins, have recently appealed as promising compounds possessing interesting structural, chemical and biological properties [8,9]. Various approaches to these compounds exist [8,10,11,12], which have been summarized in a recent review paper [13]. Despite the ongoing research in this field, the synthesis of heterocycle annelated and spiro derivatives of phosphacoumarins and dihydrophosphacoumarins is still scarce (see [14] for the review).

**Scheme 1:**
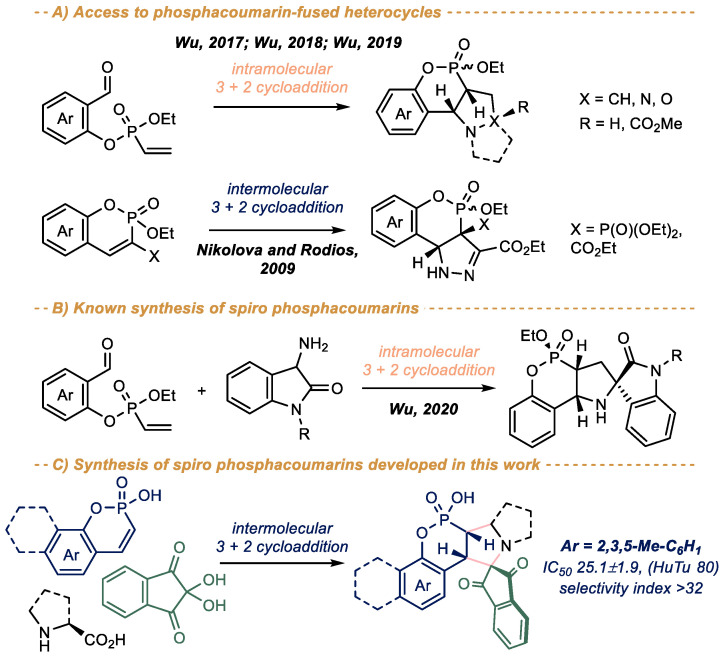
Synthesis of heterocycle-fused phosphacoumarins via [3 + 2] cycloaddion reactions [15,16,17,18] (**A**), the only reported synthesis of spiro phosphacoumarins [19] (**B**) and the complementary approach developed in this work (**C**).

The approach to the pyrazolidin-3-on-, pyrrolidine- and isoxazolidine-fused phosphacoumarins via intramolecular 1,3-dipolar cycloaddition of salicylaldehyde-derived vinylphosphonates has been developed by Wu and coworkers (Scheme 1A) [15,16,17]. Earlier, Nikolova and Rodios reported the synthesis of pyrazole-fused phosphacoumarins using intermolecular [3 + 2] cycloaddition of phosphacoumarins with ethyl diazoacetate [18]. The only synthesis of oxindole spiro phosphacoumarins has been described by Wu [19], which also involved the intramolecular [3 + 2] cycloaddition of vinylphosphonates (Scheme 1B). This is in sharp contrast to parent coumarins, for which a number of spiro derivatives are known [20,21], including rather complex polycyclic [22] and even fullerene-fused ones [23].

As a result of our ongoing research in this area [24,25,26], herein we report a highly diastereoselective synthesis of spiro dihydrophosphacoumarins via intramolecular [3 + 2] cycloaddition of phosphacoumarins with some azomethine ylides (Scheme 1C). The proposed approach is complementary to that reported by Wu [19] and provides an easy entry to the novel pyrrolidine-fused spiro-phosphacoumarins derivatives, which are inaccessible via Wu’s method. The mechanism of the reaction and its regio- and diastereoselectivity was explored using quantum chemistry calculations. The cytotoxicity of the obtained compounds towards normal and cancer cell lines was also tested, the most potent compound being 3-fold more active that reference 5-fluorouracil and exhibiting a selectivity index > 32.

## 2. Results and Discussion

### 2.1. Chemistry

**Scheme 2:**
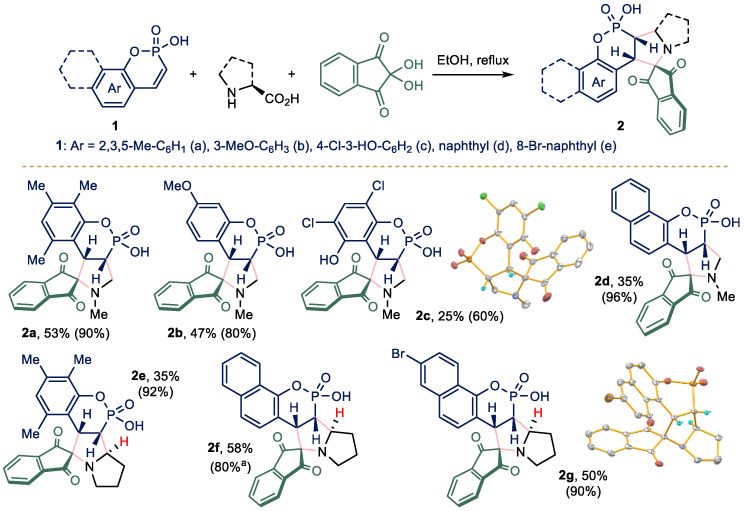
Synthesis of spiro pyrrolidinophosphacoumarins **2**. Isolated yield is given, yield according to ^31^P NMR is given in parenthesis. ^a^ Isolated as a mixture of diastereomers with *dr* 12: 1 (see discussion in the text).

We started our research with the optimization of reaction conditions using phosphacoumarin **1a**, ninhydrin and sarcosine as model compounds. Pleasingly, simple refluxing of reactants in ethanol provided the target compound **2a** with a *ca* 90% yield according to ^31^P NMR data (Scheme 2). Additionally, the reaction proceeded in a highly regio- and diastereoselective manner (*dr* > 95: 5), which was also evidenced by ^31^P NMR data. We succeeded in the isolation of the single diastereomer from the reaction mixture with a 53% yield. Convinced of the possibility of the formation of the desired spiro dihydrophophosphacoumarins, we further extended the reaction scope using phosphacoumarins **1b**–**e**. The reaction proceeded smoothly, providing target spiro compounds with a 60–96% yield according to NMR data. However, isolated yields were considerably lower due to the degradation of phosphacoumarins **2** during silica gel column purification, leading to the formation of highly polar compounds. Unfortunately, we were not able to isolate and identify these byproducts.

Presumably, the electron-donating groups in aromatic moiety promote the reaction, whereas electron withdrawing chlorine substituent clearly lowers the yield of the compound **2c**. In all cases, the target compounds were formed as single regioisomers with an excellent diastereoselectivity (*dr* > 95: 5). The configuration of the compound **2c** was determined to be *SS/RR* with X-ray analysis.

We also tested *L*-proline as an amino acid component in this reaction, which also furnished tetracyclic spiro phosphacoumarins **2e**–**g**. Despite one more stereocenter being present in these cases, the diastereoselectivity of the reaction remained fairly high (*dr* > 95: 5 according to ^31^P NMR data). Fortunately, we were able to grow crystals which were suitable for x-ray analysis of the compound **2g**, which allowed us to assign *SRS/RSR* configuration to the obtained diastereomers.

Despite the ^31^P NMR data of the reaction mixtures in all cases indicating the presence of single desired product, the compound **2f** was isolated as a mixture of diastereomers (*dr* 12: 1, see Supporting Information, Figure S31). This may be explained by the relatively low sensitivity of the ^31^P NMR method, not allowing the detection of very low concentrations (*ca* 2–3%) of the second diastereomer in the reaction mixtures. However, the reaction mixture could be enriched with this unobservable diastereomer during work-up, which is presumably the case for the compound **2f**.

### 2.2. Quantum Chemistry Studies

**Scheme 3:**
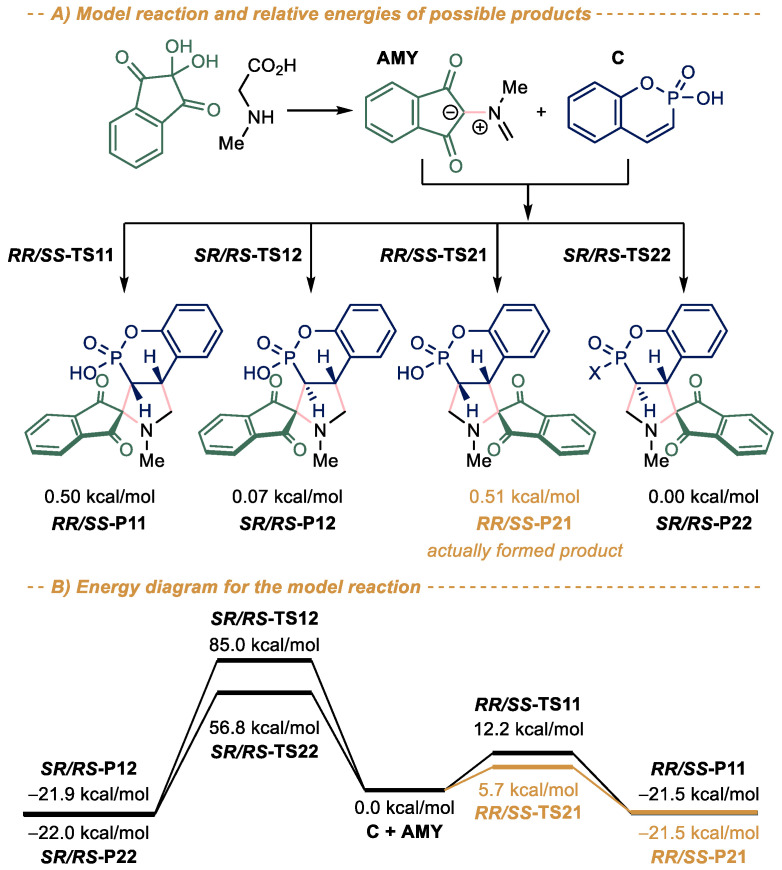
(**A**) Model [3 + 2] cycloaddition of phosphacoumarin **C** with ninhydrin-derived azomethine ylide **AMY** alongside relative energies of the possible isomeric products (ΔE, kcal/mol); (**B**) Energy diagram (ΔE, kcal/mol) as obtained from quantum chemistry calculations (PW6B95D/def2-TZVPD//B3LYP/6–31 + G*, Gaussian16). The sum of the total energies of reactants (**C** + **AMY**) is taken as zero.

In order to gain more insight into the regio- and diastereoselectivity of the reaction of phosphacoumarins with azomethine ylides, quantum chemistry studies were carried out using unsubstituted phosphacoumarin **C** as the model compound (Scheme 3A). The first stage of the reaction, i.e., the formation of azomethine ylide **AMY** from ninhydrin and sarcosine, is a well-known process (see, e.g., [27,28]) and therefore was not modelled. The second stage is the [3 + 2] dipolar cycloaddition of intermediate **AMY** and compound **C**. In principle, two regioisomeric products may be formed at this stage. Since each one can exist as two diastereomers, this gives four possible final products in total. So, to identify the most preferred regioisomer of the reaction, the quantum chemistry calculations of the reagents **C** and **AMY**, the products **P** as well as the corresponding transitions states **TS** were performed. Analysis of quantum chemical results shows that for the charged species taking solvation model with a protic ethanol solvent into account play a crucial role in stabilizing the intermediates and products of [3 + 2]-cycloaddition (see Table S2 in Supporting Information).

According to the obtained quantum chemistry data, the reaction under study is exothermic (thermal effects are 21.5–22.0 kcal/mol for all expected reaction pathways) with the compound ***SR*/*RS*-P22** being slightly lower in energy compared to all the others (Scheme 3A, see also Supporting Information, Table S1). This is somewhat counterintuitive, since one would expect isomers ***RR*/*SS*-P21** and ***SR*/*RS*-P22** to be much more unfavourable due to sterical hindrance caused by the ninhydrin moiety. However, the energy difference appeared to be *ca* 0.1–0.5 kcal/mol only. Obviously, the preferable formation of the ***RR*/*SS*-P21** isomer cannot be attributed to its thermodynamic stability.

On the other hand, the calculated transition state energies differ significantly for all products (Scheme 3B, see also Supporting Information, Table S2). The transition state energies for the ***SR*/*RS***-diastereomers are significantly higher than for their ***SS/RR***-counterparts for both regioisomers. Taking into account the energy difference (*ca* 51–73 kcal/mol), the barriers can be considered prohibitively high for the ***SR*/*RS***-diastereomers formation. On the other hand, the barrier for the formation of the ***SS*/*RR*-P21** diastereomer is *ca* 7 kcal/mol lower compared to that of the ***SS*/*RR*-P11**-diastereomer. According to the Arrhenius equation, 1 kcal/mol difference in activation energies results in more than a 6-fold difference in reaction rates at room temperature. Thus, the formation of the compound ***SS*/*RR*-P21** is much more preferable. These results are in complete agreement with the experimental observations. Thus, the high regio- and diastereoselectivity of the reaction may be attributed to the faster formation of the ***SS*/*RR*-P21**-isomer, which appears to be a product of a kinetic control.

### 2.3. Biological Studies

#### 2.3.1. In Vitro Cytotoxicity

Next, the obtained compounds were tested for cytotoxicity against normal and cancer human cell lines at concentrations of 1–100 µM. The compounds **2f** and **2g** were excluded from these studies, however, due to their extremely low solubility in water. As seen from Table 1, all of the tested compounds exhibit low cytotoxicity against MCF-7 cancer cell line, whereas the cytotoxicity against M-HeLa cells is comparable to that of the reference compound 5-fluorouracil. Notably, some of the compounds appeared to be non-toxic to normal cells in the studies’ concentrations range. Similarly, the cytotoxicity of the obtained compounds against HuTu 80 cancer cells is either lower or comparable to the cytotoxicity of 5-fluorouracil.

The remarkable exception is the phosphacoumarin **2a**. Its cytotoxicity against HuTu 80 cell line is *ca* 2.6-fold higher than the cytotoxicity of the reference compound, whereas the cytotoxicity against the Chang liver normal cell line is more than 10-fold lower, which gives a selectivity index > 32. For the M-HeLa cells, the cytotoxicity of compound **2a** was somewhat higher than that of the reference compound (52.6 ± 4.1 vs. 62.0 ± 4.7), with a selectivity index > 15. Finally, although the activity against MCF-7 cell line was considerably lower compared to the 5-fluorouracil, the selectivity index still remained above 10. Since compounds with a selectivity index > 10 are considered highly selective [29], the phosphacoumarin **2a** is a promising lead for further studies.

#### 2.3.2. Cell Cycle Analysis

**Figure 1:**
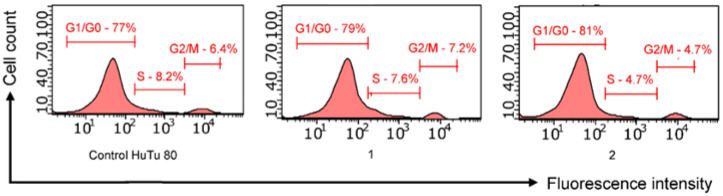
Effect of compound **2a** on cell cycle of HuTu 80 by flow cytometry. 1–**2a** at IC_50_/2 concentration (12.5 µM); 2–**2a** at IC_50_ concentration (25 µM).

Taking into account the high potency and selectivity of the compound **2a**, some additional experiments were carried out to study in more details its anti-cancer action. The mechanism of action of cytotoxic agents is often associated with cell cycle arrest, which leads to a slowing down of cell proliferation. So, we have performed a cell cycle analysis for the HuTu 80 cells using flow cytometry. According to the obtained data, the presence of compound **2a** at concentrations of IC_50_/2 (12.5 μM) and IC_50_ (25 μM) after 24 h leads to an increase in the number of cells in the G1/G0 phase up to 79.0% and 81.0%, respectively, compared with the control of 77% (Figure 1). Meanwhile, the proportion of cells in S phase decreased almost by half (8.2% vs. 4.7%). Taken together, these results indicate that the compound **2a** treatment induces G0/G1 phase arrest and reduces the S phase of the cell cycle, leading to an inhibition in the proliferation of HuTu 80 cells.

#### 2.3.3. Induction of Apoptotic Effects

**Figure 2:**
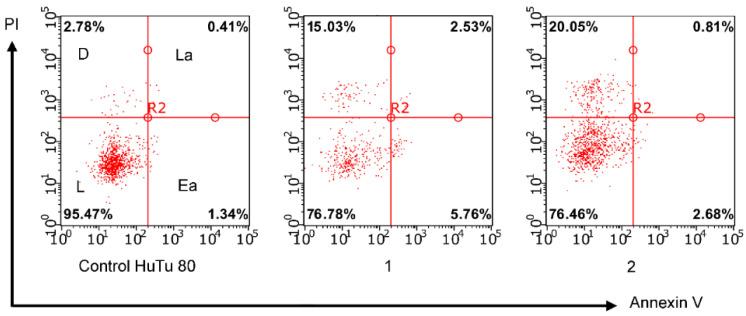
Apoptotic effects of compounds ***2a*** on HuTu 80 cells. 1–**2a** at IC_50_/2 concentration (12.5 µM); 2–**2a** at IC_50_ concentration (25 µM).

**Figure 3:**
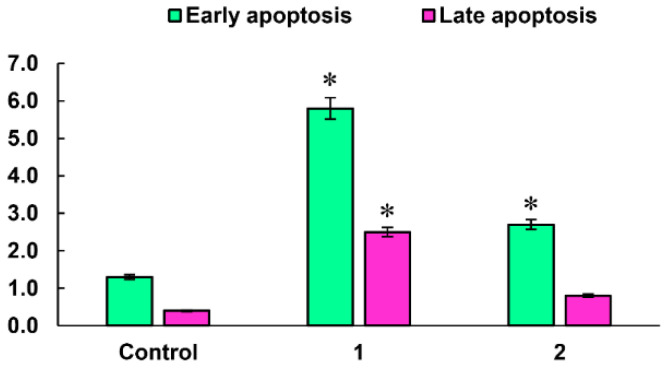
Representative histograms for the numbers of cells (% of total) in the early and late stages of apoptosis for the control and experimental groups. 1–**2a** at IC_50_/2 concentration (12.5 µM); 2–**2a** at IC_50_ concentration (25 µM). The values are presented as the mean ± SD; (∗) *p* < 0.05 compared to control.

Apoptosis is one of the most important mechanisms used to screen for new anticancer agents. The ability of the lead compound **2a** to induce apoptosis in HuTu 80 cells was determined by flow cytometry using annexin V-Alexa Fluor 647. Cells were incubated in the presence of **2a** at concentrations of IC_50_/2 and IC_50_ (Figure 2). It can be seen that after a 24-h incubation, the test compound begins to induce apoptosis in HuTu 80 cells. The most active apoptotic effects are manifested at an IC_50_/2 concentration (12.5 μM) in the early apoptosis stage (Figure 3).

#### 2.3.4. Mitochondrial Membrane Potential

**Figure 4:**
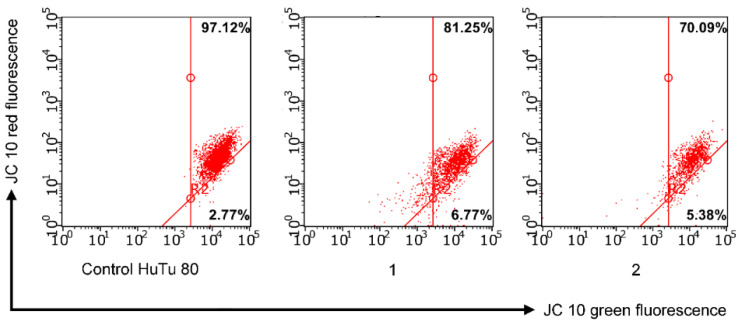
Flow cytometry analysis of HuTu 80 cells treated with compound **2a**. 1–**2a** at IC_50_/2 concentration (12.5 µM); 2–**2a** at IC_50_ concentration (25 µM).

**Figure 5:**
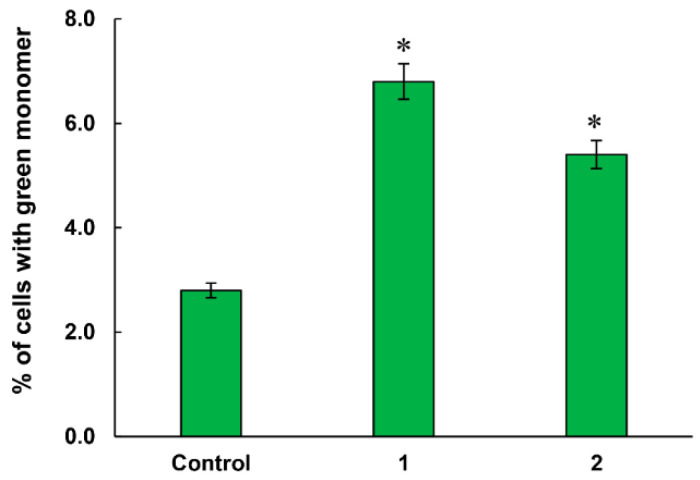
Quantitative determination of % cells with green monomers; 1–**2a** at IC_50_/2 concentration (12.5 µM); 2–**2a** at IC_50_ concentration (25 µM). Values are presented as mean ± SD; (∗) *p* < 0.05 compared to control.

The possibility of apoptosis through the mitochondrial pathway was assessed by flow cytometry using the JC-10 fluorescent dye (in the Mitochondria Membrane Potential Kit). In normal cells with a high membrane potential, JC-10 accumulates in the mitochondrial matrix, where it forms aggregates with a red fluorescence. However, in apoptotic cells, a decrease in the membrane potential occurs. JC-10 diffuses from mitochondria and turns into a monomeric form, emitting a green fluorescence, which is recorded by a flow cytometer (Figure 4). After treatment with lead compound **2a** at concentrations of IC_50_/2 and IC_50_, the intensity of the green fluorescence significantly increased compared to the control (Figure 5). The results obtained indicated a significant decrease in the mitochondrial membrane potential of HuTu 80 cells. As in the experiment with V-Alexa Fluor 647, the cytotoxic effect of compound **2a** was more pronounced at IC_50_/2 concentration. The results described above suggest that the mechanism of action of the leader of compound **2a** can be associated with the induction of apoptosis proceeding via the mitochondrial pathway.

**Figure 6:**
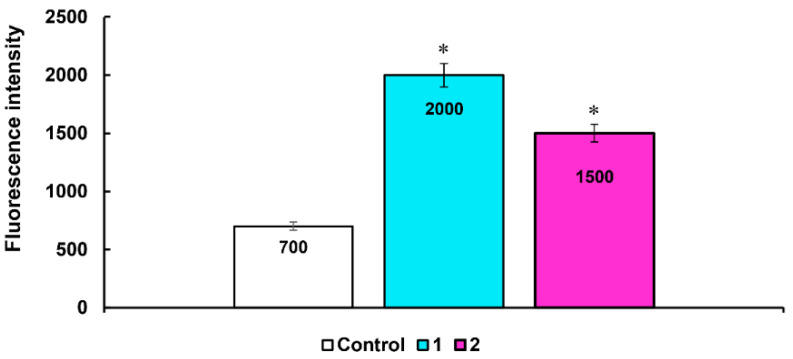
Induction of ROS production by lead compound **2a**. 1–**2a** at concentration IC_50_/2 (12.5 µM); 2–**2a** at concentration IC_50_ (25 µM); (∗) *p* < 0.05 compared to control.

An increase in the production of reactive oxygen species (ROS) by compounds also characterizes the development of apoptosis along the mitochondrial pathway. Mitochondria are a potential source and target of ROS. ROS leads to dysfunction of the mitochondria and, consequently, to irreversible cell damage. In this regard, the effect of the lead compound **2a** in HuTu 80 cells on ROS production was investigated using a flow cytometry assay and the CellROX^®^ Deep Red flow cytometry kit. The data presented in Figure 6 show a significant increase in CellROX^®^ Deep Red fluorescence intensity dominated by IC_50_/2. This indicates an increase in ROS production in the presence of compound **2a**.

## 3. Materials and Methods

### 3.1. Quantum Chemistry Calculations

All calculations were performed with Becke’s three parameter hybrid exchange functional [30] and the gradient-corrected nonlocal correlation functional of Lee et al. [31] (B3LYP) in combination with the standard 6–31 + G* basis set [32,33,34] in the Gaussian16 package [35]. All geometry optimizations were performed without symmetry constraints (see Supporting Information, Table S3 for optimized cartesian coordinates). Since the method and the basis set used are known to have many limitations [36], geometry optimization was followed by a single-point calculation with PW6B95D functional [37] and def2-TZVPD basis set [38] to improve energies. Additionally, the Polarizable Continuum Model with the CPCM polarizable conductor calculation model was used in single-point calculations as a solvation model with the molecule of interest inside a cavity in a continuous, homogenous dielectric medium. Ethanol was used as a solvent in the used solvation model.

To ensure the calculated geometries correspond to true minima, vibrational analyses were performed using the same level of theory and the structure was accepted only if all eigenvalues of the Hessian matrix were positive. The transition states were confirmed by the presence of one negative eigenvalue in the Hessian matrix of the second derivatives. Additionally, the intrinsic reaction coordinate (IRC) was traced to ensure the transition state really connects the species involved in the reaction. The energy diagram was created with the aid of the Energy Diagram Plotter (CDXML) program [39].

### 3.2. Chemistry

#### 3.2.1. General Methods

IR spectra were recorded on a UR-20 spectrometer in a 400–3600 cm^−1^ range in KBr. ^1^H NMR spectra were recorded on a Bruker MSL 400 spectrometer (399.93 MHz) with respect to the signals of residual protons in the deuterated solvent (CDCl_3_, DMSO-d6, D_2_O, CF_3_COOD). The ^13^C NMR spectra were recorded on a Bruker Avance 600 (151 MHz) spectrometer relative to the signals of residual protons from the deuterated solvent (CDCl_3_, DMSO-d6, D_2_O, CF_3_COOD) (see Supporting information, Figures S3–S30 and Figures S32–S36 for the copies of NMR spectra). The ^31^P NMR spectra were recorded on a Bruker Avance 600 (151 MHz) spectrometer. Elemental analysis was performed on a Carlo Erba device EA 1108. The melting points were determined in glass capillaries on a Stuart SMP 10 instrument.

The X-ray diffraction data for the crystals were collected on a Bruker D8 Venture diffractometer equipped with a CCD detector (Mo-Kα, λ = 0.71073 Å, graphite monochromator). Semi-empirical absorption correction was applied by the SADABS program [40]. The structures were solved by direct methods and refined by the full-matrix least squares in the anisotropic approximation for non-hydrogen atoms. The calculations were carried out by the SHELX-2014 program package [41] using Olex2 1.2 [42]. Crystallographic data for the structures reported in this paper have been deposited with the Cambridge Crystallographic Data Center (CCDC 2179367,2179368, see Supporting information, Tables S4 and S5 and Figures S1 and S2 for the detailed data). Starting phosphacoumarins **1** were obtained by reported procedures [43].

#### 3.2.2. General Procedure for the Synthesis of Compounds **2**

To a refluxing solution of phosphacoumarin 1 (1.8 mmol) in ethanol (5 mL), a mixture of ninhydrin (0.32 g, 1.8 mmol) and appropriate aminoacid (1.8 mmol) was added in one portion. The reaction mixture was refluxed until full consumption of starting materials (^31^P NMR control, *ca* 12 h). The target compounds **2** were isolated in a pure form in two ways. In case of compounds **2a**–**d** the solvent was removed in a vacuum and the residue was purified by gradient-elution column chromatography using DCM: ethanol as eluent. In the case of compounds **2e**–**g**, the precipitate formed was filtered off, washed thoroughly with distilled water and dried in vacuum.

*(3aS,9bS)-4-hydroxy-2,6,7,9-tetramethyl-2,3,3a,9b-tetrahydrospiro[benzo [5,6][1,2]oxaphosphinino [3,4-c]pyrrole-1,2’-indene]-1’,3’-dione 4-oxide (**2a**)*

Yield 0.25 g (53%), yellow solid, m.p. > 300 °C. IR (ν, cm^−1^): 1080 (C-O), 1232 (P = O), 1460 (CH_2_), 1596 (C-C_Ar_), 1705 (C = O), 1744 (C = O), 2939 (CH_3_). ^1^H NMR (600 MHz, D_2_O): 1.64 (s, 3H), 1.94 (s, 3H), 2.04 (s, 3H), 2.31 (s, 3H), 2.58 (s, 1H), 3.03–3.13 (m, 1H), 3.53–3.60 (m, 1H), 3.85–3.95 (m, 1H), 4.41 (dd, 1H, PCH, ^3^*J*_HH_ 11.74 Hz, ^2^*J*_PH_ 17.08 Hz), 6.19 (s, 1H), 7.36 (d, 1H, ^3^*J*_HH_ 7.71 Hz), 7.72 (t, 1H, ^3^*J*_HH_ 7.56 Hz), 7.85 (t, 1H, ^3^*J*_HH_ 7.55 hz), 7.99 (d, 1H, ^3^*J*_HH_ 7.69 Hz). ^13^C NMR (151 MHz, D_2_O): 11.0, 18.8, 24.8, 35.7 (d, ^1^*J*_PC_ 229.4 Hz), 49.0 (d, ^2^*J*_PC_ 10.6 Hz), 56.8, 80.1, 118.4 (d, ^2^*J*_PC_ 14.1 Hz), 122.3, 123.4, 125.7, 126.6, 134.0, 137.1, 138.0, 138.4, 141.2, 141.4, 151.1 (d, ^3^*J*_PC_ 6.3 Hz), 201.0, 201.9 (s). ^31^P NMR (243 MHz, D_2_O): 22.7. MS (MALDI TOF), *m*/*z*: 434 [M + Na]^+^. Calcd. (%) for C_22_H_24_NO_5_P: C, 63.92; H, 5.19; O, 20.01 N, 3.39; P, 7.49. Found: C, 64.23; H, 5.39; O, 19.75; N, 3.19; P, 7.43.

*(3aS,9bS)-4-hydroxy-7-methoxy-2-methyl-2,3,3a,9b-tetrahydrospiro[benzo [5,6][1,2]* *oxaphosphinino [3,4-c]pyrrole-1,2’-indene]-1’,3’-dione 4-oxide (**2b**)*

Yield 0.36 g (47%), yellow solid, m.p. > 300 °C. IR (ν, cm^−1^): 1081 (C-O), 1162 (O-CH_3_), 1229 (P = O), 1504 (CH_2_), 1618 (C-C_Ar_), 1705 (C = O), 1742 (C = O), 2930 (CH_3_). ^1^H NMR (600 MHz, D_2_O): 2.18 (s, 3H, NCH_3_); 3.03–3.12 (m, 1H, CH_2_); 3.37–3.44 (mp, 1H, CH); 3.54 (s, 3H, OCH_3_); 3.70–3.79 (m, 1H, CH_2_) 4.05–4.12 (m, 1H, PCH); 5.97 (d, 1H, CH, ^3^*J*_HH_ 8.19 Hz); 6.17 (d, 1H, CH, ^3^*J*_HH_ 8.43 Hz); 6.44 (s, 1H, CH); 7.34 (d, 1H, CH, ^3^*J*_HH_ 7.54 Hz); 7.73 (t, 1H, CH, ^3^*J*_HH_ 7.15 Hz); 7.85 (t, 1H, CH, ^3^*J*_HH_ 7.49 Hz); 7.96 (d, 1H, CH, ^3^*J*_HH_ 7.57 Hz). ^13^C NMR (100.6 MHz, D_2_O): 35.7 (s); 35.81 (d, ^1^*J*_PC_ 136.4 Hz); 52.0 (d, ^2^*J*_PC_ 4.4 Hz); 56.0 (s); 57.3 (s); 80.8 (s); 106.2 (d, ^2^*J*_PC_ 4.3 Hz); 109.8 (s); 115.2 (s); 115.4 (s); 122.8 (s); 124.1 (s); 130.8 (s); 137.7 (s); 138.4 (s); 142.1 (d, ^3^*J*_PC_ 2.3 Hz); 153.7 (d, ^3^*J*_PC_ 5.9 Hz); 160.2 (s); 202.7 (s); 203.5 (s). ^31^P NMR (242.9 MHz, D_2_O): 21.3. MS (MALDI-TOF) *m*/*z*: 420 [M + Na]^+^. Calcd: (%) for C_20_H_18_NO_6_P: C, 60.15; H, 4.54; O, 24.04; N, 3.51; P, 7.76. Found: C, 60.23; H, 4.39; O, 23.95; N, 3.27; P, 7.52.

*(3aS,9bS)-6,8-dichloro-4,9-dihydroxy-2-methyl-2,3,3a,9b-tetrahydrospiro[benzo [5,6][1,2]* *oxaphosphinino [3,4-c]pyrrole-1,2’-indene]-1’,3’-dione 4-oxide (**2c**)*

Yield 0.17 g (25%), yellow solid, m.p. > 300 °C. IR (ν, cm^−1^):1078 (C-Cl), 1229 (P = O), 1456 (CH_2_), 1591 (C-C_Ar_), 1703 (C = O), 1736 (C = O), 2871 (CH_3_). ^1^H NMR (399.93 MHz, D_2_O): 2.21 (s, 3H, NCH_3_); 3.01–3.15 (m, 1H, CH_2_); 3.37–3.44 (m, 1H, CH); 3.64–3.75 (m, 1H, CH_2_); 4.36–4.46 (m, 1H, PCH); 7.25 (s, 1H, CH); 7.54 (d, 1H, CH, ^3^*J*_HH_ 7.86 Hz); 7.85 (t, 1H, CH, ^3^*J*_HH_ 7.18 Hz); 7.94 (t, 1H, CH, ^3^*J*_HH_ 7.76 Hz); 8.01 (d, 1H, CH, ^3^*J*_HH_ 7.48 Hz). ^13^C NMR (100.6 MHz, D_2_O): 34.4 (d, ^1^*J*_PC_ 128.6 Hz); 46.5 (s); 56.9 (s); 78.6 (s); 114.6 (d, ^2^*J*_PC_ 15.2 Hz); 115.5 (s); 122.2 (s); 123.4 (s); 129.0 (s); 137.4 (s); 139.9 (s); 142.4 (s); 148.0 (d, ^3^*J*_PC_ 4.9 Hz); 202.2 (s); 203.1 (s). ^31^P NMR (161.9 MHz, D_2_O): 21.2. MS (MALDI-TOF) *m*/*z*: 492 [M + K]^+^. Calcd: (%) for C_19_H_14_NCl_2_O_6_P: C, 50.24; H, 3.11; O, 21.14; Cl, 15.61; N, 3.08; P, 6.82. Found: C, 50.23; H, 3.39; O, 20.95; N, 3.17; P, 6.41.

*(3a’S,11a’S)-11’-hydroxy-2’-methyl-1’,2’,3a’,11a’-tetrahydrospiro[indene-2,3’-naphtho [2’,1’:5,6][1,2]* *oxaphosphinino [3,4-c]pyrrole]-1,3-dione 11’-oxide (**2d**)*

Yield 0.25 g (34.7%), yellow solid, m.p. > 300 °C. IR (ν, cm^−1^): 1243 (P = O), 1466 (CH_2_), 1596 (C-C_Ar_), 1703 (C = O), 1749 (C = O). ^1^H NMR (399.93 MHz, D_2_O): 2.25 (s, 3H, NCH_3_); 3.23–3.33 (m, 1H, CH_2_); 3.51–3.58 (m, 1H, CH); 3.84–3.96 (m, 1H, CH_2_); 4.97 (dd, 1H, PCH, ^3^*J*_HH_ 11.87 Hz, ^2^*J*_PH_ 16.02 Hz); 7.04–7.09 (m, 2H, CH); 7.13 (t, 1H, CH, ^3^*J*_HH_ 7.56 Hz); 7.19 (t, 1H, CH, ^3^*J*_HH_ 7.84 Hz); 7.40 (t, 1H, CH, ^3^*J*_HH_ 7.62 Hz); 7.42–7.52 (m, 3H, CH); 7.59 (d, 1H, CH, ^3^*J*_HH_ 8.87 Hz); 7.64 (d, 1H, CH, ^3^*J*_HH_ 7.73 Hz). ^13^C NMR (100.6 MHz, D_2_O): 35.6 (d, ^1^*J*_PC_ 136.9 Hz); 47.7 (d, ^2^*J*_PC_ 4.35 Hz); 56.7 (s); 80.6 (s); 116.4 (d, ^2^*J*_PC_ 15.5 Hz); 120.5 (d, ^3^*J*_PC_ 10.1 Hz); 121.3 (d, ^3^*J*_PC_ 9.0 Hz); 122.9 (s); 123.3 (s); 124.7 (s); 127.1 (s); 128.6 (s); 130.1 (s); 130.5 (s); 131.5 (s); 136.4 (s); 137.2 (s); 140.2 (s); 141.7 (s); 151.2 (d, ^3^*J*_PC_ 6.1 Hz); 202.2 (s); 203.0 (s). ^31^P NMR (161.9 MHz, D_2_O): 23.6. MS (MALDI-TOF) *m*/*z*: 420 [M + 1]^+^, 442 [M + Na]^+^. Calcd: (%) for C_23_H_18_NO_5_P: C, 65.87; H, 4.33; O, 19.08; N, 3.34; P, 7.39. Found: C, 65.65; H, 4.31; O, 19.01; N, 3.21; P. 7.40.

*(6aS,6bR,11aS)-6-hydroxy-1,3,4-trimethyl-6a,6b,7,8,9,11a-hexahydrospiro[benzo [5,6][1,2]oxaphosphinino* *[3,4-a]pyrrolizine-11,2’-indene]-1’,3’-dione 6-oxide (**2e**)*

Yield 0.28 g (35%), white solid, m.p. > 300 °C. IR (ν, cm^−1^): 1252 (P = O), 1460 (CH_2_), 1591 (C-C_Ar_), 1708 (C = O), 1751 (C = O), 2929 (CH_3_).^1^H NMR (399.93 MHz, D_2_O): 1.82 (s, 3H, CH_3_); 1.97 (s, 3H, CH_3_); 2.01 (s, 3H, CH_3_); 2.10–2.29 (m, 2H, CH_2_); 2.44–2.57 (m, 2H, CH_2_); 2.87–3.00 (m, 1H, CH_2_); 3.72–3.80 (m, 1H, CH); 4.23–4.32 (m, 1H, CH_2_); 4.95 (dd, 1H, PCH, ^3^*J*_HH_ 11.50 Hz, ^2^*J*_PH_ 18.93 Hz); 5.51 (s, 1H, CH); 6.26 (s, 1H, CH); 7.49–7.52 (m, 1H, CH); 7.81 (td, 1H, CH, ^3^*J*_HH_ 6.35 Hz); 7.87 (td, 1H, CH, ^3^*J*_HH_ 7.58 Hz); 8.00–8.04 (m, 1H, CH). ^13^C NMR (100.6 MHz, D_2_O): 10.7 (s); 18.4 (s); 18.7 (s); 23.5 (s); 29.5 (d, ^3^*J*_PC_ 3.4 Hz) 41.5 (d, ^1^*J*_PC_ 135.1 Hz); 47.1 (d, ^2^*J*_PC_ 5.2 Hz); 50.4 (s); 72.0 (s); 77.1 (s); 115.6 (d, ^2^*J*_PC_ 14.2 Hz); 122.9 (s); 123.9 (s); 125.9 (d, ^3^*J*_PC_ 3.3 Hz); 127.3 (s); 134.3 (s); 137.3 (s); 138.1 (s); 139.5 (s); 139.9 (s); 141.0 (s); 150.5 (d, ^3^*J*_PC_ 6.8 Hz); 192.9 (s); 193.8 (s). ^31^P NMR (161.9 MHz, D_2_O): 19.0. MS (MALDI-TOF) *m*/*z*: 460 [M + Na]^+^. Calcd: (%) for C_24_H_24_NO_5_P: C, 65.90; H, 5.53; O, 18.29; N, 3.20; P, 7.08. Found: C, 65.65; H, 5.31; O, 18.11; N, 3.11; P. 7.00.

*(8a’S,8b’R,13a’S)-8’-hydroxy-8a’,8b’,9’,10’,11’,13a’-hexahydrospiro[indene-2,13’-naphtho [2’,1’:5,6][1,2]* *oxaphosphinino [3,4-a]pyrrolizine]-1,3-dione 8’-oxide (**2f**)*

Yield 0.44 g (58%), white solid, m.p. > 300 °C. IR (ν, cm^−1^): 1223 (P = O), 1465 (CH_2_), 1594 (C-C_Ar_), 1715 (C = O), 1753 (C = O). ^1^H NMR (399.93 MHz, CF_3_COOD): 2.29–2.41 (m, 1H, CH_2_); 2.50–2.62 (m, 1H, CH_2_); 2.65–2.73 (m, 1H, CH_2_); 2.87–2.96 (m, 1H, CH_2_); 3.82–3.92 (m, 1H, CH_2_); 3.98–4.09 (m, 1H, CH_2_); 4.25–4.35 (m, 1H, CH); 4.39–4.45 (m, 1H, CH); 5.06–5.18 (m, 1H, PCH); 7.39 (d, 1H, CH, ^3^*J*_HH_ 8.94 Hz); 7.71–7.82 (m, 2H, CH); 7.86–7.95 (m, 2H, CH); 8.05 (d, 1H, CH, ^3^*J*_HH_ 8.97 Hz); 8.11–8.18 (m, 2H, CH); 8.47 (d, 1H, CH, ^3^*J*_HH_ 7.31 Hz). ^13^C NMR (100.6 MHz, CF_3_COOD): 7.5 (s); 23.2 (s); 25.4 (s); 37.2 (d, ^1^*J*_PC_ 140.9 Hz); 42.9 (s); 51.0 (s); 61.5 (s); 68.5 (d, ^3^*J*_PC_ 4.5 Hz); 72.3 (s); 77.4 (d, ^2^*J*_PC_ 11.9 Hz); 105.6 (d, ^2^*J*_PC_ 11.0 Hz); 115.7 (d, ^3^*J*_PC_ 5.7 Hz); 121.0 (s); 121.9 (s); 122.1 (s); 122.2 (s); 123.0 (s); 123.8 (s); 126.1 (s); 126.6 (s); 126.9 (s); 127.4 (s); 127.9 (s); 143.8 (d, ^3^*J*_PC_ 6.8 Hz); 147.2 (s); 192.6 (s). ^31^P NMR (161.9 MHz, CF_3_COOD): 18.9. MS (ESI) *m*/*z*: 448 [M + 3]^+^. Calcd: (%) for C_25_H_20_NO_5_P: C, 67.41; H, 4.53; O, 17.96; N, 3.14; P, 6.95. Found: C, 67.65; H, 4.31; O, 18.01; N, 3.01; P. 6.85.

*(8a’S,8b’R,13a’S)-4’-bromo-8’-hydroxy-8a’,8b’,9’,10’,11’,13a’-hexahydrospiro[indene-2,13’-naphtho [2’,1’:5,6][1,2]* *oxaphosphinino [3,4-a]pyrrolizine]-1,3-dione 8’-oxide (**2g**)*

Yield 0.23 g (50%), white solid, m.p. > 300 °C. IR (ν, cm^−1^): 1085 (C-Br), 1251 (P = O), 1500 (CH_2_), 1588 (C-C_Ar_), 1713 (C = O), 1755 (C = O), 2926 (CH_3_). ^1^H NMR (399.93 MHz, DMSO-d_6_): 1.73–1.84 (m, 1H, CH_2_); 1.87–1.94 (m, 1H, CH_2_); 2.05–2.16 (m, 2H, CH_2_); 2.87–2.97 (m, 2H, CH_2_); 3.47–3.55 (m, 1H, CH); 4.75–4.83 (m, 1H, CH); 5.19–5.26 (m, 1H, PCH); 7.09 (d, 1H, CH, ^3^*J*_HH_ 8.85 Hz); 7.30–7.36 (m, 2H, CH); 7.43 (d, 1H, CH, ^3^*J*_HH_ 9.26 Hz); 7.62 (d, 1H, CH, ^3^*J*_HH_ 8.92 Hz); 7.(td, 1H, CH, ^3^*J*_HH_ 6.55 Hz); 7.71 (td, 1H, CH, ^3^*J*_HH_ 8.61 Hz); 7.78 (d, 1H, CH, ^3^*J*_HH_ 7.58 Hz); 7.89 (d, 1H, CH, ^3^*J*_HH_ 2.1 Hz). ^13^C NMR (100.6 MHz, DMSO-d_6_): 24.0 (s); 29.0 (s); 41.4 (d, ^1^*J*_PC_ 131.9 Hz); 45.4 (s); 48.7 (s); 69.9 (s); 77.7 (s); 117.2 (d, ^2^*J*_PC_ 14.9 Hz); 117.7 (s); 119.7 (s); 122.5 (d, ^3^*J*_PC_ 3.0 Hz); 122.9 (s); 123.7 (s); 125.4 (s); 129.3 (s); 129.6 (s); 130.2 (s); 130.7 (s); 131.5 (s); 136.4 (s); 137.4 (s); 140.1 (s); 141.5 (s); 152.2 (d, ^3^*J*_PC_ 6.3 Hz). ^31^P NMR (161.9 MHz, DMSO-d_6_): 20.06. MS (ESI) *m*/*z*: 520 [M-1]. Calcd: (%) for C_25_H_19_NBrO_5_P: C, 57.27; H, 3.65; O, 15.26; Br, 15.24; N, 2.67; P, 5.91. Found: C, 57.25; H, 3.61; O, 15.01; Br, 15.31; N, 2.61; P. 5.85.

*1-methyl-1,3-dihydro-4H-spiro[chromeno [4,3-b]pyrrole-2,2’-indene]-1’,3’,4-trione (**4**)*

Yield 0.25 g (25%), orange solid, m.p. > 300 °C. IR (ν, cm^−1^): 1511 (CH_2_), 1594 (C-C_Ar_), 1614 (C = C), 1707 (C = O), 1750 (C = O). ^1^H NMR (399.93 MHz, CDCl_3_): 3.25 (s, 3H, NCH_3_); 3.28 (s, 2H, CH_2_); 7.24–7.30 (m, 1H, CH); 7.43 (d, 1H, CH, ^3^*J*_HH_ 8.04 Hz); 7.57 (t, 1H, CH, ^3^*J*_HH_ 8.12 Hz); 7.99 (d, 1H, CH, ^3^*J*_HH_ 8.11 Hz); 7.97–8.04 (m, 2H, CH); 8.10–8.15 (m, 2H, CH). ^13^C NMR (100.6 MHz, CDCl_3_): 35.0 (s); 36.2 (s); 78.0 (s); 96.0 (s); 113.2 (s); 118.4 (s); 123.1 (s); 123.4 (s); 124.6 (s); 132.0 (s); 137.1 (s); 141.1 (s); 155.0 (s); 158.1 (s); 159.3 (s); 196.5. MS (ESI) *m*/*z*: 330 [M-1]. Calcd: (%) for C_20_H_13_NO_4_: C, 72.50; H, 3.95; O, 19.32; N, 4.23. Found: C, 72.10; H, 3.91; O, 19.31; N, 4.15.

### 3.3. Biological Studies

#### 3.3.1. Cell Toxicity Assay (MTT-Test)

The toxic effect on cells was determined using the colorimetric method of cell proliferation MTT (Thiazolyl Blue Tetrazolium Bromide, Sigma). For this, 10 μL of MTT reagent in Hank’s balanced salt solution (HBSS) (final concentration 0.5 mg/ml) was added to each well. The plates were incubated at 37 °C for 2–3 h in an atmosphere humidified with 5% CO_2_. Absorbance was recorded at 540 nm using an Invitrologic microplate reader (Russia). Experiments for all compounds were repeated three times. The M-HeLa clone 11 human, epithelioid cervical carcinoma, strain of HeLa, clone of M–HeLa; human duodenal cancer cell line (HuTu 80); human breast adenocarcinoma cells (MCF-7) and Chang liver cell line (Human liver cells) from the N. F. Gamaleya Research Center of Epidemiology and Microbiology and the Type Culture Collection of the Institute of Cytology (Russian Academy of Sciences) were used in the experiments. The cells were cultured on a standard nutrient medium “Igla” produced by the Moscow Institute of Poliomyelitis and Viral Encephalitis. M.P. Chumakov by PanEco, with the addition of 10% fetal calf serum and 1% nonessential amino acids (NEAA).

The cells were sown on a 96-well panel from Eppendorf at a concentration of 5 × 10^3^ cells per well with a volume of 100 μL medium, and cultured in a CO_2_ incubator at 37 °C. In 48 h after planting the cells, the culture medium was taken into the wells, and 100 μL solutions of the studied drug in the specified dilutions were added to the wells. Dilutions of the compounds were prepared directly in growth medium supplemented with 5% DMSO to improve solubility. The cytotoxic effect of the test compounds was determined at concentrations of 0.1–100 μM. The calculation of the IC_50_, the concentration of the drug causing inhibition of cell growth by 50%, was performed using the program: MLA—“Quest Graph ™ IC50 Calculator”. AAT Bioquest, Inc., Pleasanton, CA, USA, https://www.aatbio.com/tools/ic50-calculator (accessed on 25 January 2022).

#### 3.3.2. Induction of Apoptotic Effects by Test Compounds (Flow Cytometry Assay)

*Cell Culture.* HuTu 80 cells at 1 × 10^6^ cells/well in a final volume of 2 mL were seeded into six-well plates. After 24 h of incubation, various concentrations of compound ***2a*** were added to wells.

*Cell Apoptosis Analysis.* The cells were harvested at 2000 rpm for 5 min and then washed twice with ice-cold PBS, followed by resuspension in binding buffer. Next, the samples were incubated with 5 μL of annexin V- Alexa Fluor 647 (Sigma-Aldrich, St. Louis, MO, USA) and 5 μL of propidium iodide for 15 min at room temperature in the dark. Finally, the cells were analyzed by flow cytometry (Guava easy Cyte, MERCK, Rahway, NJ, USA) within 1 h. The experiments were repeated three times.

#### 3.3.3. Mitochondrial Membrane Potential

Cells were harvested at 2000 rpm for 5 min and then washed twice with ice-cold PBS, followed by resuspension in JC-10 (10 µg/mL) and incubation at 37 °C for 10 min. After the cells were rinsed three times and suspended in PBS, the JC-10 fluorescence was observed by flow cytometry (Guava easy Cyte, MERCK, Rahway, NJ, USA).

#### 3.3.4. Detection of Intracellular ROS

HuTu 80 cells were incubated with compound ***2a*** at concentrations of IC_50_ for 24 h. ROS generation was investigated using flow cytometry assay and CellROX^®^ Deep Red flow cytometry kit. For this HuTu 80 cells were harvested at 2000 rpm for 5 min and then washed twice with ice-cold PBS, followed by resuspension in 0.1 mL of medium without FBS, to which was added 0.2 μL of CellROX^®^ Deep Red and incubated at 37 °C for 30 min After being washed three times, the cells were suspended in PBS, and the production of ROS in the cells was immediately monitored using a flow cytometer Guava easy Cyte, MERCK, Rahway, NJ, USA).

#### 3.3.5. Statistical Analysis

The IC_50_ values were calculated using the online calculator MLA—Quest Graph™ IC50 Calculator AAT Bioquest, Inc., Pleasanton, CA, USA, 25 January 2022. The statistical analysis was performed using the Mann-Whitney test (*p* < 0.05). Tabular and graphical data contains averages and standard errors.

## 4. Conclusions

In conclusion, a series of novel pyrrolidine-fused spiro dihydrophosphacoumarins were obtained via intermolecular [3 + 2] cycloaddition of phosphacoumarins with ninhydrin-based azomethine ylides. The reaction proceeded in a highly regioselective manner, leading to the formation of up to three stereocentres with excellent diastereoselectivity. The mechanism of the reaction was studied with quantum chemistry methods. The obtained results were in a good agreement with the experimental data and indicate that the preferential formation of a single regio- and diastereoisomer is due to kinetic reasons. Additionally, a novel pathway of the reaction of 4-hydroxycoumarin with azomethine ylides has been revealed, which will be a subject for further research. The anti-cancer activities of spiro phosphacoumarins were tested in vitro. The compound possessing three methyl groups in aromatic moiety appeared to be the most potent against all tested cancer cell lines (M-HeLa, HuTu 80 and MCF-7). Its cytotoxicity was up to 2.6-fold higher than the cytotoxicity of areference compound. At the same time, its cytotoxicity against normal cell lines (Chang liver) was much lower, thus giving the selectivity index ranging from 10 (M-HeLa cell line) to >32 (HuTu 80 cell line). The more detailed studies of the anti-cancer activity of the lead compound revealed that it arrests the cell cycle at the G1/G0 phase and leads to an increased level of ROS in HuTu 80 cells, as well as decreasing the mitochondrial potential. Thus, the death of cancer cells presumably occurs via an intrinsic mitochondrial apoptosis pathway.

## Figures and Tables

**Table 1:** Cytotoxic effects of phosphacoumarins **2** on the cancer and normal human cell lines ^1^.

Cmpd	Cancer Cell Line						Normal Cell Line
	M-HeLa		MCF-7		HuTu 80		Chang Liver
	IC_50_, µM	SI	IC_50_, µM	SI	IC_50_, µM	SI	IC_50_, µM
**2a**	52.6 ± 4.1	>15	82.3 ± 7.5	>10	25.1 ± 1.9	>32	>800
**2b**	59.7 ± 4.6	1	>100	ns	53.4 ± 4.2	1.2	62.0 ± 5.5
**2c**	>100	ns	77.6 ± 6.2	ns	100 ± 8.4	ns	>100
**2d**	60 ± 5.4	ns	92.2 ± 8.3	ns	82.6 ± 7.6	ns	57.0 ± 4.3
**2e**	>100	ns	>100	ns	>100	ns	>100
**5-fluorouracil**	62.0 ± 4.7	1.4	16.7 ± 1.3	5	65.2 ± 5.6	1.3	86.3 ± 6.5

^1^ Three independent experiments were carried out; “ns” means no selectivity.

## Data Availability

The data presented in this study are contained within the article or in Supplementary Materials, or are available upon request from the corresponding authors Ayrat Khamatgalimov or Almir Gazizov.

## Supplementary Materials

The following supporting information can be downloaded at: https://www.mdpi.com/article/10.3390/ijms232214348/s1.
