# Supplementary material for: Diastereoselective Synthesis of Novel Spiro-Phosphacoumarins and Evaluation of Their Anti-Cancer Activity
Source: Int J Mol Sci. 2022 Nov 18;23(22):14348. doi: 10.3390/ijms232214348 (PMC9695012; doi:10.3390/ijms232214348)
Supplement: Supplementary file 1 [file ijms-23-14348-s001.zip › ijms-2026325-supplementary.pdf]

SUPPLEMENTARY MATERIALS

Diastereoselective synthesis of novel spiro-phosphacoumarins and evaluation of their anti-cancer activity

Valeriia V. Sennikova,[a] Alena V. Zalaltdinova,[a] Yulia M. Sadykova,[a] Ayrat R. Khamatgalimov,[a] Almir S. Gazizov,[a] Alexandra D. Voloshina,[a] Anna P. Lyubina,[a] Syumbelya K. Amerhanova,[a] Julia K. Voronina,[b] Elena A. Chugunova,[a] Nurbol O. Appazov,[c] A.R. Burilov,[a] M.A. Pudovik[a]

[a] *Arbuzov Institute of Organic and Physical Chemistry, FRC Kazan Scientific Center, Russian Academy of Sciences, 420088, Arbuzova str., 8, Kazan, Russian Federation*

[b] *N.S. Kurnakov Institute of General and Inorganic Chemistry, Russian Academy of Sciences, Leninsky Ave. 31, 119991 Moscow, Russia*

[c] *Korkyt Ata Kyzylorda State University, 29A Aiteke Bi St., Kyzylorda 120014, Kazakhstan*

Contents

Synthesis of starting phosphacoumarins 2

Quantum chemistry data 2

X-Ray data 10

References 12

Copies of NMR spectra 13

Synthesis of starting phosphacoumarins

The phosphacoumarins **1** were obtained as described previously.[1] Herein, we provide the synthesis of compound **1a** as the representative example:

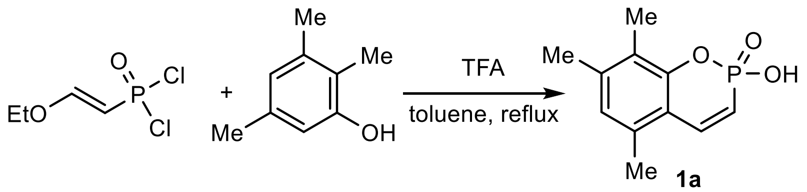

A solution of (2-ethoxyvinyl)phosphonic dichloride (1.90 g, 5.3 mmol) in toluene (5 mL) was added dropwise to the refluxing mixture of 2,3,5-trimethylphenol (1.44 g, 10.6 mmol) and trifluoroacetic acid (0.4 g, 5.3 mmol) in toluene (30 mL). The reaction mixture was refluxed for 3 h. The solvent was decanted from the oily precipitate, the isopropyl alcohol was added to the residue and refluxed until a white precipitate formed. The precipitate thus formed was filtered and dried to a constant weight.

Yield 85%; m.p. 206-208 ºC; IR (KBr); v = 1225 (P = O), 1590, 1617(CH_{arom}), 2924 (CH_3) cm^{-1}. ^{1}H NMR (DMSO-d_6, 400 MHz): 2.14 (s, 3H), 2.24 (s, 3H), 2.34 (s, 3H), 6.24(dd $^3J_{HH}$ = 13.0 Hz, $^3J_{PH}$ = 21.0 Hz, 1H), 6.85 (s, 1H), 7.51 (dd $^3J_{HH}$ = 13.0 Hz, $^2J_{PH}$ = 42.0 Hz, 1H). ^{31}P NMR (DMSO-d_6, 162 MHz): 5.17. Anal. Calcd for $C_{11}H_{13}O_3P$: C, 58.93; H, 5.84; P 13.82. Found: C, 58.40, H, 5.84; P,13.66%. MS (MALDI-TOF) $[M]^+$ 224, $[M+Na]^+$ 247.

Quantum chemistry data

Table S1 Relative energies (ΔE, kcal/mol), enthalpies (ΔH, kcal/mol), Gibbs energies (ΔG, kcal/mol) and HOMO-LUMO gaps (eV) of possible regioisomers products as obtained from quantum chemistry calculations (B3LYP/6–31+G*, Gaussian16)

Reaction products	ΔE	ΔH	ΔG	HOMO-LUMO
***RR/SS*-P11**	0.77	0.54	1.19	3.84
***SR/RS*-P12**	0.00	0.00	0.00	3.43
***RR/SS*-P21**	1.22	0.96	1.54	3.64
***SR/RS*-P22**	0.42	0.31	0.68	3.35

Table S2 Relative energies of possible products and transition states (kcal/mol) as obtained from quantum chemistry calculations (Gaussian16)[a]

	***RR/SS*-11**		***SR/RS*-12**		***RR/SS*-21**		***SR/RS*-22**	
	gas phase (B3LYP/ 6–31+G*)	CPCM-model (PW6B95D/ def2-TZVPD)	gas phase (B3LYP/ 6–31+G*)	CPCM-model (PW6B95D/ def2-TZVPD)	gas phase (B3LYP/ 6–31+G*)	CPCM-model (PW6B95D/ def2-TZVPD)	gas phase (B3LYP/ 6–31+G*)	CPCM-model (PW6B95D/ def2-TZVPD)
**TS**	18.76	12.19	80.27	85.00	11.71	5.71	63.95	56.83
**P**	-11.65	-21.52	-12.42	-21.95	-11.21	-21.51	-12.00	-22.02

[a] *Sum of the total energies of reactants (**C + AMY**, see discussion in the text) is taken as zero*

Table S3 Optimized cartesian coordinates for reactants, transition states and products as obtained from quantum chemistry calculations (B3LYP/6–31+G*, Gaussian16)

Atom	X	Y	Z
	**phosphacoumarin C**		
C	3.111251	-1.125966	0.006715
C	1.775702	-1.523351	-0.019027
C	0.772083	-0.554344	-0.028539
C	1.080408	0.821209	-0.017476
C	2.439644	1.191434	0.007818
C	3.448416	0.234783	0.020971
H	3.892554	-1.881029	0.016773
H	1.492113	-2.570931	-0.030311
C	0.026604	1.821655	-0.039203
H	2.690268	2.249822	0.017663
H	4.490217	0.540816	0.041756
C	-1.296481	1.549530	-0.024183
H	0.350539	2.861409	-0.071125
H	-2.034456	2.346738	-0.034182
O	-0.524625	-1.004677	-0.076800
O	-2.687195	-0.484790	1.322299
P	-1.892238	-0.117775	0.122820
O	-2.680678	-0.429765	-1.263765
H	-3.503442	-0.916040	-1.079209
	**azomethyne ylide AMY**		
C	-1.077545	-0.698186	-0.000023
C	-1.110228	0.702012	-0.000041
C	-2.312634	1.395476	-0.000044
C	-3.500596	0.646604	-0.000023
C	-3.468121	-0.753859	0.000005
C	-2.246460	-1.446475	0.000009
C	0.352097	-1.165788	-0.000053
C	1.153735	0.050927	-0.000031
C	0.297085	1.222616	-0.000042
H	-2.326270	2.481789	-0.000056
H	-2.208752	-2.532253	0.000026
O	0.707365	-2.347851	0.000032
O	0.622808	2.416800	-0.000024
N	2.539571	0.110541	0.000024
C	3.283514	-1.176816	-0.000007
H	3.006026	-1.751836	0.882629
H	3.005301	-1.752246	-0.882132
H	4.350242	-0.953303	-0.000479
C	3.230576	1.225055	0.000151
H	2.697461	2.166979	0.000229
H	4.310310	1.157209	0.000144
H	-4.459337	1.159187	-0.000017
H	-4.401894	-1.310305	0.000023
	***RR/SS*-TS11**		
C	-5.119986	-0.878997	1.013043
C	-3.932144	-1.585734	0.827866
C	-2.838037	-0.952045	0.239679
C	-2.896314	0.391699	-0.168046
C	-4.107844	1.077229	0.022543

C	-5.212285	0.455908	0.603036
C	-1.716341	1.071887	-0.758252
C	-0.616348	0.334134	-1.254769
O	-1.678572	-1.693642	0.101670
O	0.702576	-2.139140	-0.799410
P	-0.571014	-1.427895	-1.078059
O	-1.295157	-1.969695	-2.441068
H	-5.973655	-1.372665	1.469243
H	-3.833312	-2.624151	1.128567
H	-4.178471	2.114999	-0.298346
H	-6.138758	1.007757	0.734730
H	-1.967993	1.973369	-1.318582
H	0.000904	0.750354	-2.044868
H	-0.919875	-2.828558	-2.704471
C	3.226524	0.289251	-0.079586
C	2.607170	-0.364662	0.993602
C	3.198272	-1.462347	1.608444
C	4.435436	-1.895189	1.118744
C	5.055692	-1.243005	0.040310
C	4.454300	-0.139506	-0.575282
C	2.381254	1.430130	-0.530267
C	1.150175	1.380271	0.295832
C	1.307142	0.296668	1.303261
O	2.653386	2.225853	-1.426511
O	0.562179	0.027407	2.239769
N	0.260506	2.406668	0.438168
C	0.444241	3.665989	-0.305650
C	-1.002220	2.086994	0.837194
H	2.701945	-1.968737	2.430622
H	4.922861	-2.753785	1.573196
H	6.015985	-1.603115	-0.319613
H	4.924464	0.373281	-1.409440
H	0.271263	3.512673	-1.375956
H	-0.266700	4.394135	0.089082
H	1.461090	4.031928	-0.175495
H	-1.071964	1.277321	1.552863
H	-1.673526	2.928639	0.986376

***RR/SS*-TS21**

C	-0.874603	3.941410	-0.037745
C	-1.859433	3.096527	0.474563
C	-1.866562	1.753864	0.110949
C	-0.893910	1.211926	-0.762940
C	0.085461	2.099185	-1.268657
C	0.097840	3.442183	-0.916049
C	-0.911941	-0.176316	-1.145630
C	-1.862917	-1.108441	-0.682042
O	-2.868186	0.964982	0.649622
O	-4.181844	-1.243347	0.933959
P	-3.386746	-0.433399	-0.021674
O	-4.219242	0.016180	-1.347206
H	-0.868748	4.990355	0.245292
H	-2.626199	3.459502	1.151625
H	0.840655	1.705567	-1.944545
H	0.861718	4.101524	-1.317959

H	-0.204578	-0.485269	-1.909998
H	-2.006574	-1.998798	-1.293051
H	-5.178359	-0.057011	-1.196842
C	2.582207	0.197847	0.748435
C	3.140208	-0.604763	-0.255286
C	4.351015	-0.276332	-0.852464
C	5.004263	0.885078	-0.412001
C	4.448019	1.685763	0.595644
C	3.221849	1.349535	1.189435
C	1.284510	-0.401702	1.199510
C	1.087498	-1.597789	0.375923
C	2.242309	-1.778387	-0.510310
O	0.569026	0.045907	2.098616
O	2.457639	-2.675092	-1.328560
N	0.065485	-2.502197	0.550531
C	0.129626	-3.822612	-0.100154
C	-1.146420	-2.048351	0.959264
H	4.775254	-0.907850	-1.628060
H	5.956718	1.166804	-0.853708
H	4.975970	2.578718	0.920610
H	2.780351	1.962763	1.969778
H	-0.678615	-4.436127	0.301847
H	1.090828	-4.290479	0.108306
H	0.028715	-3.728245	-1.186035
H	-1.118217	-1.200952	1.633659
H	-1.899385	-2.804946	1.156366

***SR*/*RS*-TS12**

C	-5.663120	-0.369462	0.794422
C	-4.648984	-1.312966	0.630479
C	-3.364302	-0.879188	0.267957
C	-3.073777	0.472813	0.073132
C	-4.107065	1.398989	0.249121
C	-5.388201	0.992898	0.619077
C	-1.660689	0.841068	-0.361170
C	-0.580958	0.021244	0.120136
O	-2.395045	-1.880684	0.120485
O	-0.024179	-2.863529	0.015520
P	-0.855847	-1.702115	-0.41687
O	-1.003013	-1.570746	-2.036757
H	-6.663262	-0.698981	1.084316
H	-4.833079	-2.381041	0.763893
H	-3.906055	2.453850	0.087552
H	-6.182804	1.731920	0.750674
H	-1.618162	0.831931	-1.471413
H	-0.717063	-0.038838	1.205964
H	-0.779930	-2.410276	-2.469155
C	3.078212	-0.172522	-0.408096
C	3.006499	0.019839	0.985034
C	3.966922	-0.529294	1.835191
C	5.001599	-1.284482	1.264237
C	5.071680	-1.481371	-0.133998
C	4.120665	-0.915169	-0.984249
C	1.928646	0.489324	-1.091631
C	0.886622	0.982348	0.023990

C	1.800947	0.813163	1.327535
O	1.813654	0.698811	-2.283343
O	1.541714	1.289568	2.420207
N	0.666879	2.476052	-0.168459
C	1.777674	3.369567	-0.503937
C	-0.609789	2.730193	-0.855355
H	3.896110	-0.378565	2.907296
H	5.752343	-1.739679	1.909368
H	5.881738	-2.080228	-0.533779
H	4.170206	-1.051714	-2.054219
H	2.628575	3.196806	0.181035
H	2.145824	3.256972	-1.554453
H	1.453695	4.403522	-0.340246
H	-1.290561	3.332257	-0.216259
H	-0.488554	3.262127	-1.818381

***SR/RS*-TS22**

C	-0.844837	4.125151	-0.253734
C	-2.022670	3.368312	-0.347074
C	-1.968315	1.976449	-0.182937
C	-0.711778	1.332911	0.035495
C	0.448763	2.115768	0.087652
C	0.394695	3.511125	-0.039810
C	-0.874693	0.131368	0.012482
C	-1.918159	0.570631	0.838865
O	-3.189356	1.299430	-0.293577
O	-4.762153	0.662436	0.383318
P	-3.385240	0.366528	-0.097907
O	-3.078285	-0.992351	-1.579940
H	-0.907237	5.209168	-0.353410
H	-2.982039	3.833200	-0.513493
H	1.417282	1.628800	0.246457
H	1.304273	4.114827	0.026333
H	-0.723331	-0.627964	-0.978244
H	-2.048996	0.078430	1.715194
H	-3.914997	-1.270340	-2.003115
C	2.825084	-0.163873	0.664849
C	2.900815	-0.630467	-0.664862
C	3.927135	-0.241646	-1.511370
C	4.901128	0.634431	-1.000346
C	4.826477	1.103358	0.327182
C	3.785351	0.707000	1.180417
C	1.624873	-0.766655	1.327896
C	0.919619	-1.525687	0.275463
C	1.745942	-1.570706	-0.934769
O	1.313306	-0.637444	2.517257
O	1.597201	-2.222304	-1.961500
N	-0.154319	-2.388848	0.588821
C	-0.624624	-3.324992	-0.464548
C	-0.961823	-2.151485	1.625527
H	3.974541	-0.612744	-2.530196
H	5.724587	0.952110	-1.637868
H	5.596319	1.776253	0.703935
H	3.713493	1.068415	2.205125
H	-1.056770	-2.760133	-1.298073

H	-1.376738	-3.980627	-0.014304
H	0.220501	-3.907337	-0.837808
H	-0.553839	-1.534804	2.437510
H	-1.811805	-2.808524	1.796106
	***RR/SS*-P11**		
C	5.201236	-0.719688	-1.041770
C	4.069688	-1.478178	-0.749045
C	2.911714	-0.846851	-0.293126
C	2.845197	0.542330	-0.115849
C	4.007807	1.277722	-0.400179
C	5.173287	0.666763	-0.860994
C	1.594193	1.282507	0.315182
C	0.429896	0.455338	0.952816
O	1.819869	-1.675486	-0.044824
O	-0.441250	-2.232965	1.061745
P	0.73404	-1.327787	1.117087
O	1.580282	-1.384761	2.509546
H	6.101790	-1.210616	-1.400469
H	4.059593	-2.557128	-0.869311
H	3.990316	2.356276	-0.254750
H	6.053255	1.267297	-1.074101
H	1.899686	2.037320	1.048475
H	0.241135	0.809286	1.971941
H	1.505552	-2.255937	2.936377
C	-3.208983	0.160616	0.212231
C	-2.690216	-0.378239	-0.971576
C	-3.502430	-1.085001	-1.858839
C	-4.851647	-1.231329	-1.532243
C	-5.373846	-0.689456	-0.342081
C	-4.556758	0.009413	0.547109
C	-2.135201	0.828364	0.979072
C	-0.844199	0.820994	0.119608
C	-1.232427	-0.112937	-1.065439
O	-2.218221	1.287709	2.104022
O	-0.482624	-0.506959	-1.937389
N	-0.539569	2.130234	-0.509489
C	-0.831563	3.323508	0.298020
C	0.878474	2.008168	-0.875022
H	-3.085849	-1.509842	-2.767207
H	-5.511647	-1.777278	-2.201261
H	-6.427829	-0.824245	-0.113979
H	-4.945673	0.424081	1.472494
H	-0.336018	3.340316	1.282301
H	-0.507517	4.197272	-0.275129
H	-1.907513	3.414567	0.461926
H	0.966930	1.394087	-1.774730
H	1.300397	2.995139	-1.084526
	***RR/SS*-P12**		
C	5.553555	-0.569772	-0.213752
C	4.469857	-1.436855	-0.344852
C	3.174495	-0.960010	-0.142742
C	2.925607	0.386011	0.181627
C	4.035758	1.232833	0.312620
C	5.338053	0.770425	0.118496

C	1.511940	0.862318	0.434692
C	0.475551	0.093581	-0.417951
O	2.161816	-1.907168	-0.308337
O	-0.272905	-2.647903	-0.791090
P	0.566018	-1.676342	-0.049705
O	0.396242	-1.750922	1.566980
H	6.561864	-0.942734	-0.371386
H	4.605422	-2.482375	-0.603662
H	3.873221	2.275018	0.575286
H	6.176819	1.452552	0.224938
H	1.267035	0.681804	1.489244
H	0.754815	0.160417	-1.479878
H	-0.010881	-2.591800	1.837136
C	-2.882224	0.072155	0.688889
C	-3.020248	0.007067	-0.706421
C	-4.153238	-0.559710	-1.293989
C	-5.146077	-1.058955	-0.450319
C	-5.010298	-0.990598	0.949772
C	-3.877760	-0.423216	1.535026
C	-1.593539	0.714995	1.048075
C	-0.795688	0.959481	-0.266542
C	-1.838182	0.610995	-1.370993
O	-1.240251	1.065165	2.160722
O	-1.689004	0.803419	-2.560246
N	-0.217958	2.287734	-0.405379
C	-1.054885	3.445684	-0.143094
C	1.153833	2.332887	0.128705
H	-4.245911	-0.608176	-2.374886
H	-6.039670	-1.507649	-0.876017
H	-5.802212	-1.386641	1.580124
H	-3.763093	-0.363528	2.613562
H	-2.001127	3.351783	-0.687249
H	-1.272986	3.596883	0.928558
H	-0.550567	4.341365	-0.522236
H	1.825863	2.774431	-0.620243
H	1.210611	2.943024	1.042210

***SR/RS*-P21**

C	-1.283884	3.862096	-0.343758
C	-2.057206	2.962290	0.387690
C	-1.882999	1.590833	0.203034
C	-0.942970	1.079230	-0.702968
C	-0.198987	2.008986	-1.448074
C	-0.355213	3.384106	-1.273137
C	-0.686460	-0.405946	-0.895408
C	-1.759948	-1.413477	-0.363178
O	-2.683487	0.755948	0.977031
O	-3.999441	-1.425041	1.377054
P	-3.236068	8-0.65409	10.366142
O	-4.086946	-0.233334	-0.961527
H	-1.416329	4.930627	-0.197133
H	-2.796917	3.299475	1.107282
H	0.512232	1.641258	-2.184760
H	0.238679	4.076187	-1.863700
H	-0.569200	-0.576689	-1.970447

H	-2.115066	-2.032424	-1.193702
H	-5.034870	-0.144831	-0.761055
C	2.575391	0.085566	0.908701
C	3.010790	-0.250654	-0.379365
C	4.336295	-0.046645	-0.772453
C	5.217023	0.495195	0.164393
C	4.778832	0.833640	1.459365
C	3.451624	0.636554	1.845062
C	1.120823	-0.194316	1.050099
C	0.651889	-0.927640	-0.238472
C	1.879564	-0.784354	-1.172388
O	0.433476	0.074398	2.015762
O	1.887740	-1.036062	-2.364774
N	0.405127	-2.325996	0.184662
C	0.660897	-3.370814	-0.817033
C	-0.988385	-2.301779	0.657527
H	4.661152	-0.306568	-1.775801
H	6.256256	0.662473	-0.105921
H	5.487695	1.258144	2.165431
H	3.100683	0.901263	2.838173
H	0.382802	-4.330452	-0.371761
H	1.726256	-3.412674	-1.054785
H	0.107131	-3.242877	-1.761143
H	-1.019437	-1.858580	1.655876
H	-1.389134	-3.315505	0.722503
		***SR/RS*-P22**	
C	-1.493813	3.997005	-0.213598
C	-2.513754	3.082684	0.039193
C	-2.245976	1.713177	0.007489
C	-0.953749	1.218956	-0.257034
C	0.044966	2.165485-	-0.529659
C	-0.207827	3.536754	-0.506107
C	-0.715478	-0.276351	-0.335167
C	-1.674125	-1.106500	0.550258
O	-3.346306	0.898551	0.268120
O	-4.511897	-1.328120	0.873710
P	-3.383641	-0.729468	0.122213
O	-3.429330	-1.002682	-1.485183
H	-1.706244	5.062239	-0.187336
H	-3.526109	3.405924	0.260563
H	1.047167	1.825508	-0.771265
H	0.594884	4.237971	-0.715895
H	-0.877659	-0.590312	-1.377579
H	-1.558462	-0.796220	1.596828
H	-4.320955	-1.267932	-1.768899
C	2.795433	0.103352	0.756221
C	2.949302	-0.257734	-0.591435
C	4.156135	-0.044536	-1.263103
C	5.207841	0.532514	-0.549690
C	5.053471	0.895206	0.802325
C	3.844160	0.689048	1.468700
C	1.412636	-0.202357	1.216945
C	0.662963	-0.895230	0.043334
C	1.683772	-0.831274	-1.118312

O	0.954777	0.024610	2.319768
O	1.477561	-1.211099	-2.257571
7	0.328914	-2.281361	0.452043
C	1.162942	-3.388979	0.010079
C	-1.118038	-2.513427	0.372398
H	4.261990	-0.323761	-2.307535
H	6.161302	0.707058	-1.041208
H	5.890926	1.342968	1.330934
H	3.711100	0.970781	2.509256
H	1.059408	-3.619579	-1.063819
H	0.886064	-4.278925	0.585946
H	2.217708	-3.180657	0.220064
H	-1.432986	-3.200628	1.165036
H	-1.419528	-2.939714	-0.600370

X-Ray data

Table S4 The detailed x-ray data for the compounds **2c** and **2g**

**Compound**	**2c**	**2g**
Chemical formula	$2(C_{19}H_{13}Cl_2NO_6P)\cdot H_{12}MgO_6\cdot 6(H_2O)$	$C_{25}H_{19}BrNO_5P\cdot C_2H_3N\cdot 2(H_2O)$
M_r	1146.85	601.38
Crystal system	Monoclinic	Monoclinic
Space group	$P2_1/c$	$P2_1/c$
Temperature (K)	100	100
a, *b*, *c* (Å)	18.7333(14)<br>8.5707(6)<br>17.6005(12)	13.6142(12)<br>17.6579(15)<br>11.1130 (9)
β (°)	116.541 (3)	102.538 (3)
V (Å^3)	2528.1 (3)	2607.8 (4)
Z	2	4
Radiation type		
m (mm^{-1})	0.39	1.69
Crystal size (mm)	0.18 × 0.16 × 0.12	0.16 × 0.12 × 0.08
Diffractometer	Bruker *APEX*-II CCD	Bruker *D8 Venture*
T_{min}, T_{max}	0.571, 0.746	0.429, 0.494
No. of measured reflections	19592	25374
No. of independent reflections	4978	5122
No. of observed [I > 2s(I)] reflections	3048	4363
R_{int}	0.110	0.033
R_1/ $wR(F^2)$, [I>2σ]	0.0628 / 0.1361	0.0296 / 0.0678
R_1/ $wR(F^2)$, (all data)	0.1190 / 0.1613	0.0392 / 0.0717
GOOF	0.998	1.047
No. of reflections	4978	5122
No. of parameters	364	364
No. of restraints	1	0
Dρ_{max}, Dρ_{min} (e Å^{-3})	0.76 / −0.64	0.39 / −0.51
**CCDC no**	2179367	2179368

Table S5 H-bonds in crystals of compounds **2c** and **2g**

H-bond	D – H, Å	H...A, Å	D...A, Å	D - H...A, °
		**2c**		
O1S-H1SA...O1	0.87	1.88	2.749(4)	178
O1S-H1SB...O1W	0.87	1.95	2.740(5)	151
O2S-H2SA...O2	0.87	1.84	2.683(5)	161
O2S-H2SB...O1W	0.87	2.24	2.842(6)	126
O3S-H3SA...O4	0.88	2.53	3.226(6)	136
O3S-H3SA...N1	0.88	2.13	2.836(6)	137'
O3S-H3SB...O2W	0.88	2.31	3.125(10)	155
O3S-H3SB...O3W	0.88	2.45	2.754(9)	101'
O2W'-H2WC...O3W'	0.87	1.73	2.560(12)	157
O2W'-H2WC...O3W	0.87	1.91	2.745(12)	161'
O2W'-H2WD...O3W'	0.87	2.37	2.841(13)	115
O1W-H1WA...O2	0.98	1.77	2.743(4)	174
O1W-H1WB...O4	0.98	1.99	2.859(5)	147
O3W'-H3WC...O4	0.87	2.15	3.012(10)	172
O3W'-H3WD...O2W'	0.87	1.91	2.560(12)	131
O3W'-H3WD...O2W	0.87	2.04	2.836(12)	152'
O18-H18...Cl2	0.81(6)	2.62(8)	3.013(4)	111(6)
O18-H18...O1	0.81(6)	1.91(7)	2.614(4)	145(8)'
C2-H2B...O11	0.99	2.58	3.141(7)	116
C9-H9...O11	0.95	2.54	3.212(7)	128
C12-H12...O18	1.00	2.38	2.731(5)	100
		**2g**		
N1-H1...O2	0.94(3)	1.67(3)	2.603(2)	174(2)
O1W-H1WA...O1	0.87(4)	1.89(4)	2.765(2)	176(4)
O1W-H1WB...O4	0.85(4)	2.04(3)	2.855(2)	161(3)
O2W-H2WA...O1W	0.89(3)	1.91(3)	2.776(2)	166(3)
O2W-H2WB...O2	0.87(3)	2.12(3)	2.936(2)	158(3)
C2-H2...O11	1.00	2.51	3.033(3)	112
C7-H7...N1S	0.95	2.52	3.331(3)	143
C12-H12...O1	1.00	2.21	3.191(2)	165
C19-H19...O1	0.95	2.58	3.379(3)	142
C20-H20...O2W	0.95	2.44	3.307(3)	151
C23-H23A...O2W	0.99	2.56	3.465(3)	152
C23-H23B...O1	0.99	2.47	3.439(2)	166
C25-H25B...O2W	0.99	2.55	3.234(3)	126

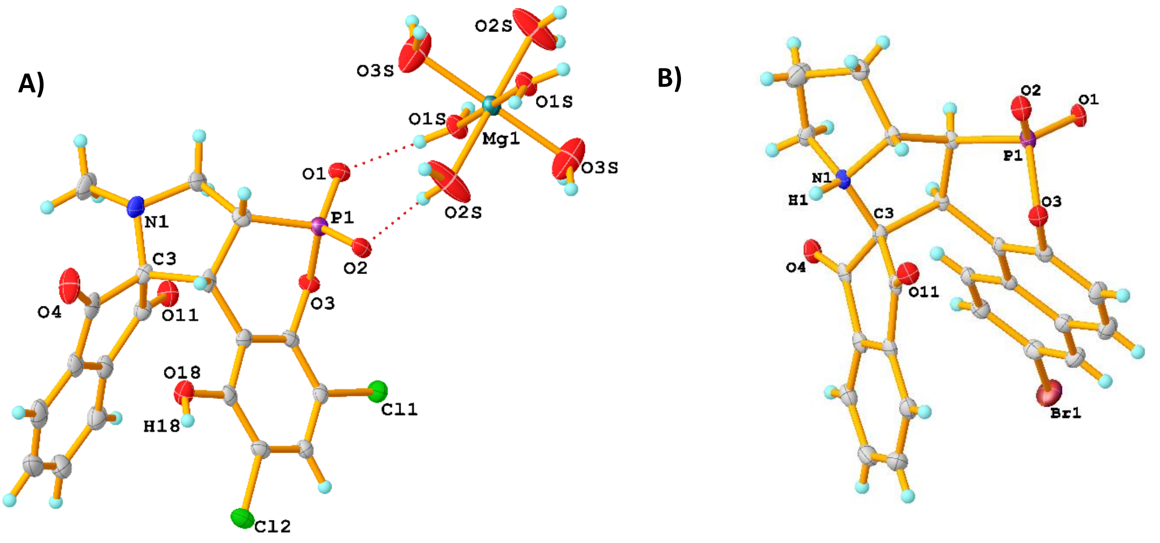

Figure S1 Molecular structure of compounds **2c** (A) and **2g** (B), ellipsoids are shown with 50% probability

The molecular and crystal structure of compounds **2c** and **2g** were determined by X-ray analysis (Figure S1). Compound **2c** crystallizes as a coordination salt with hexaaqua-magnesium and solvated by three water molecules. Compound **2g** is the crystal solvate with acetonitrile and two water molecules. The structure of polycyclic condensed structures in the compounds **2c** and is almost identical except for the position of the ninhydrin moiety. This is explained by the difference in the conformation of the five-membered cycle, as a result of which the C3 atom leaves the plane of the bicyclic fragment by 0.162 and 0.179 Å in compounds **2c** and **2g** respectively. The nitrogen-containing five -membered cycle is in the envelope conformation with the deviation from the plane of the nitrogen atom N1 by 0.269Å in compound **2c** and 0.241Å in compound **2g**. Hexaaqua-magnesium cation in crystal **2c** and all solvate water molecules are bound to the main substance by classical O-H…O and N-H…O hydrogen bonds, acetonitrile molecule in **2c** – by C-H…O interaction (Table S5).

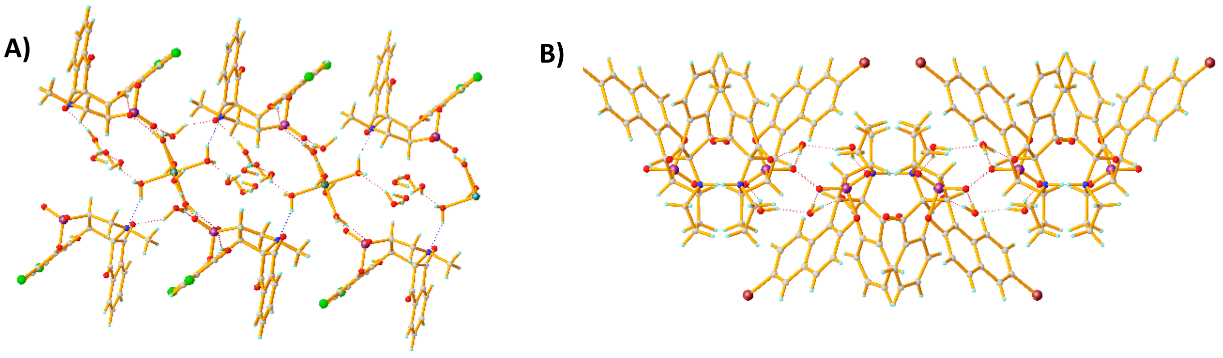

Figure S2 Fragments of crystal packing of the compound **2c** (A) and **2g** (B)

The molecular packing in crystals represents infinite layers parallel to the b0c axis. The layers are formed mainly by the classical hydrogen bonds. The three-dimensional systems are formed by the weak C-H…π and C-H…O interactions.

References

1. Y.M. Sadykova, L.M. Sadikova, A.R. Badrtdinova, A.B. Dobrynin, A.R. Burilov, M.A. Pudovik. Condensation of 2-Ethoxyvinylphosphonic Acid Dichloroanhydride with 2,3,5-Trimethylphenol. Novel Method for Preparation of Phosphacoumarins. *Phosphorus. Sulfur. Silicon Relat. Elem.*, **2015**, *190*, 2267–2272. DOI: 10.1080/10426507.2015.1073283.

Copies of NMR spectra

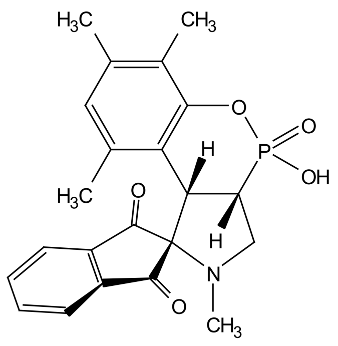

2a

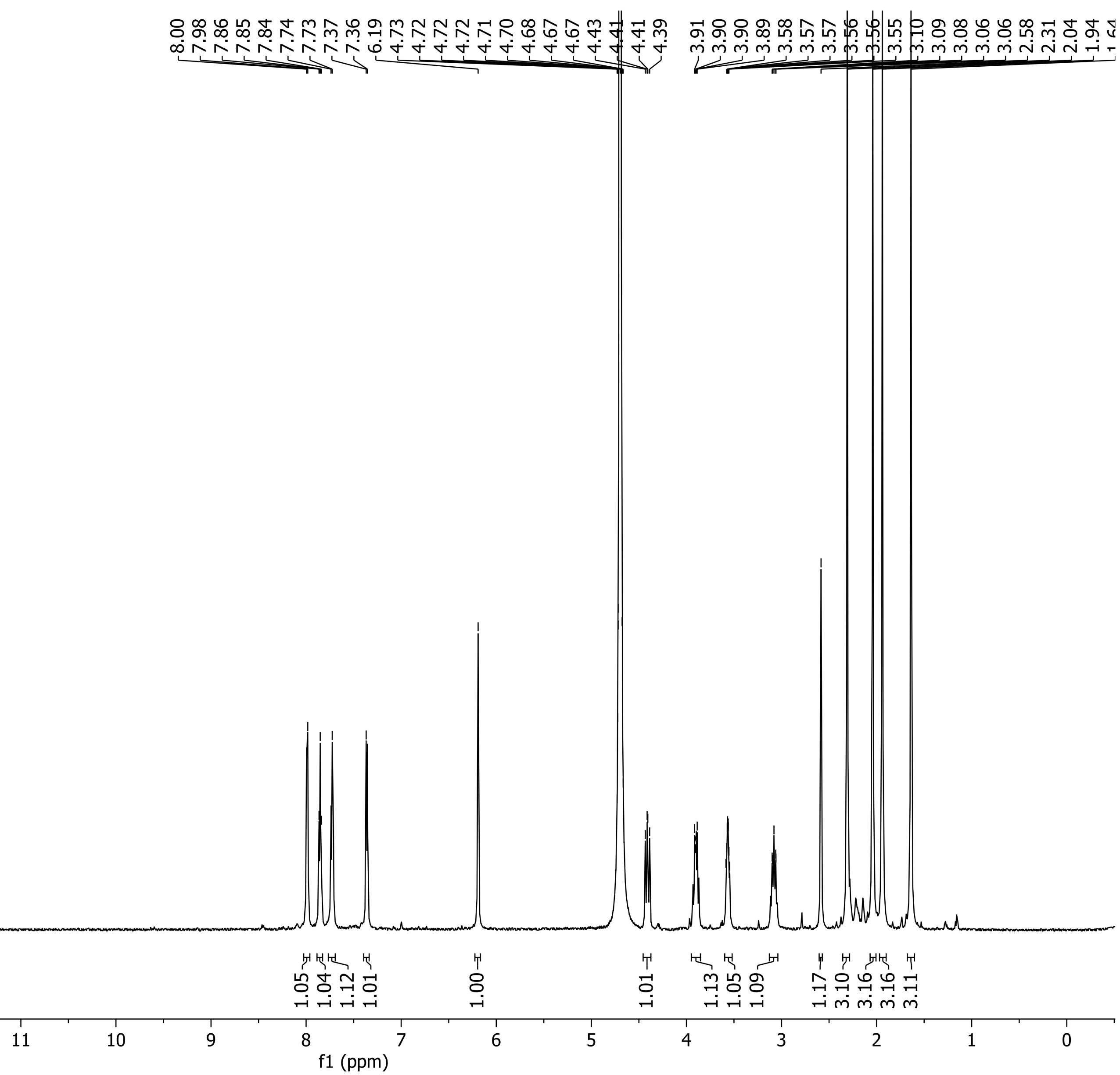

Figure S3. ^{1}H NMR spectrum (D_2O, 600MHz) of the compound **2a**

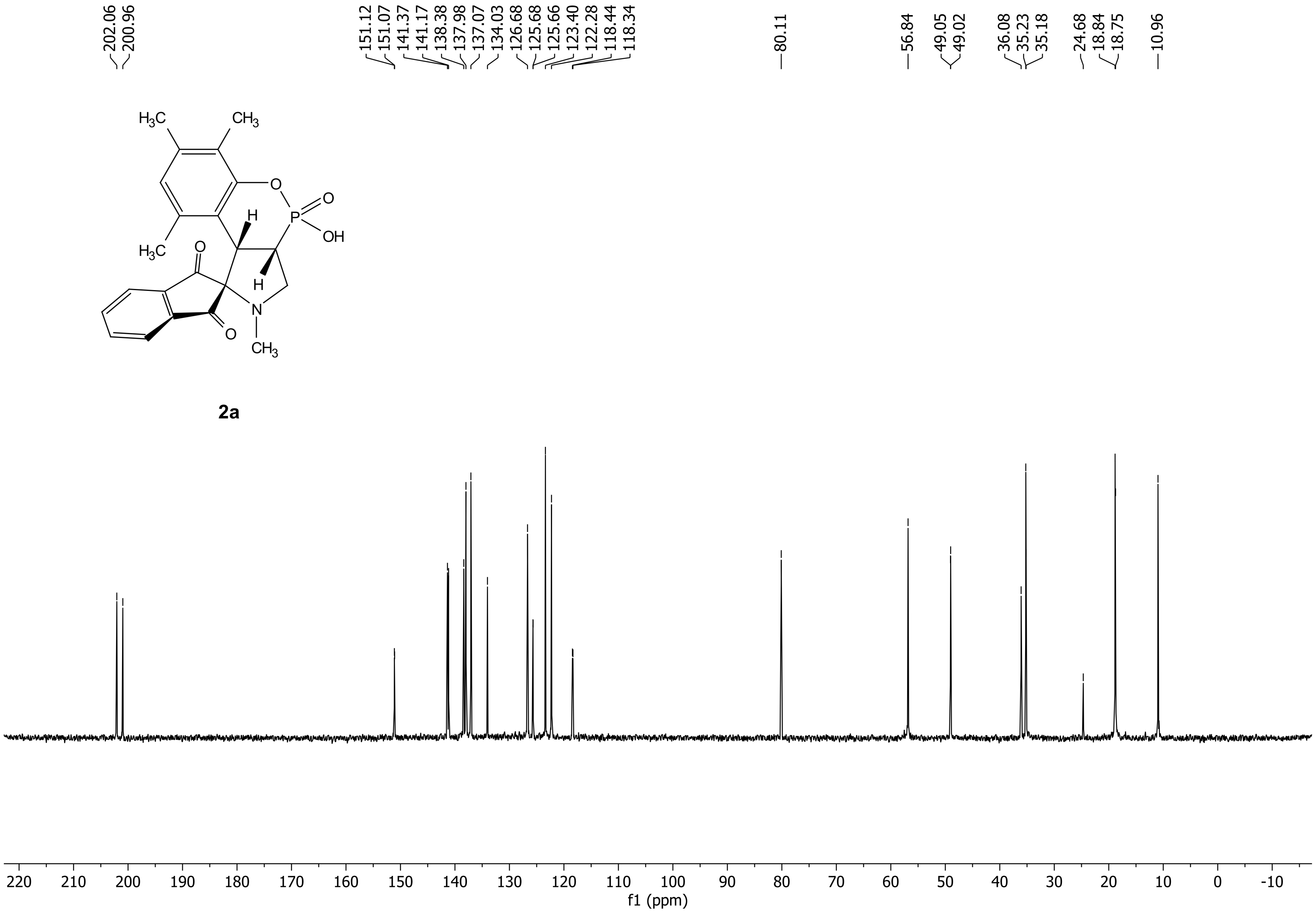

Figure S4. {^{1}H}-^{13}C NMR spectrum (D_2O, 151MHz) of the compound **2a**

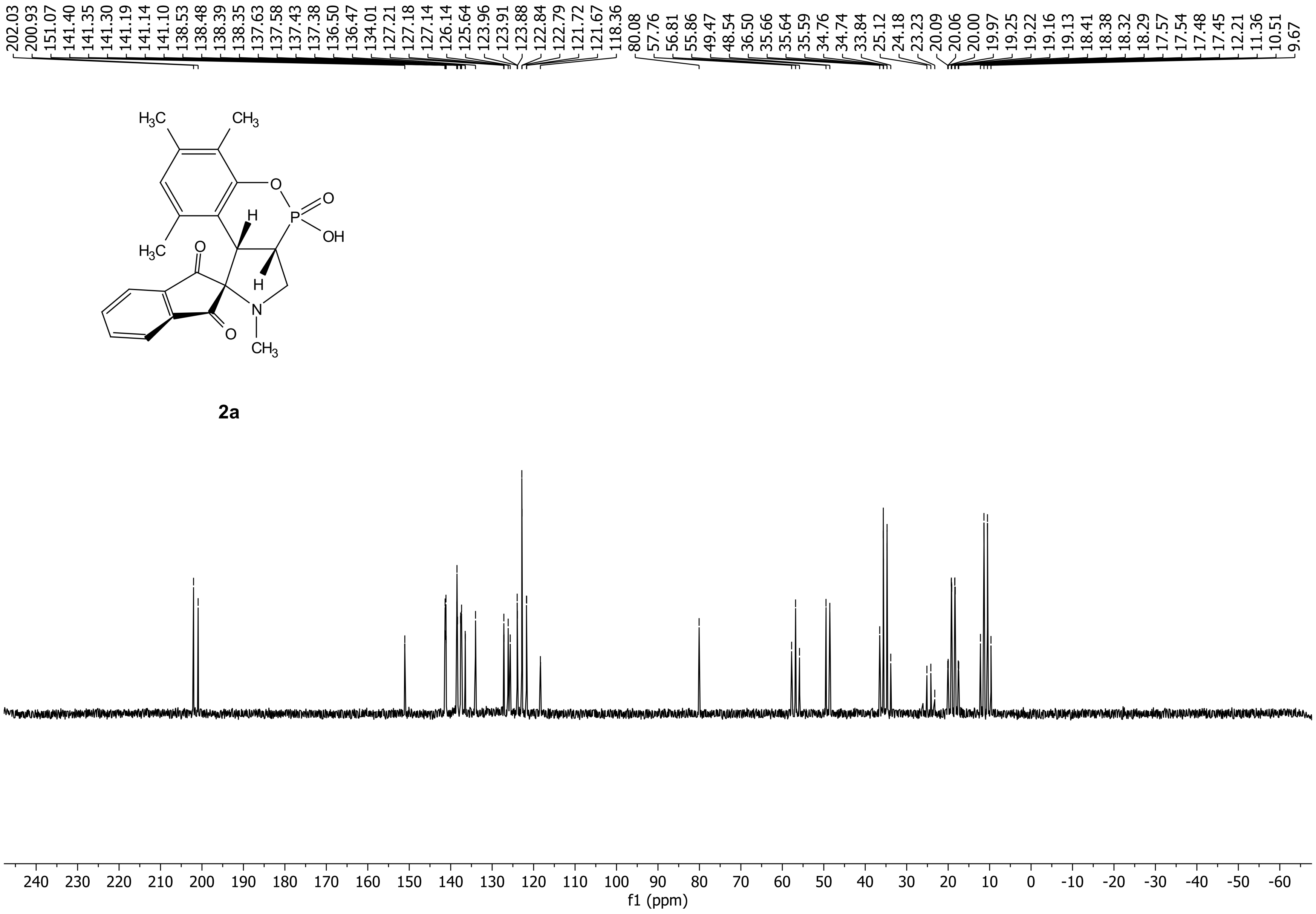

Figure S5. ^{13}C NMR spectrum (D_2O, 151MHz) of the compound **2a**

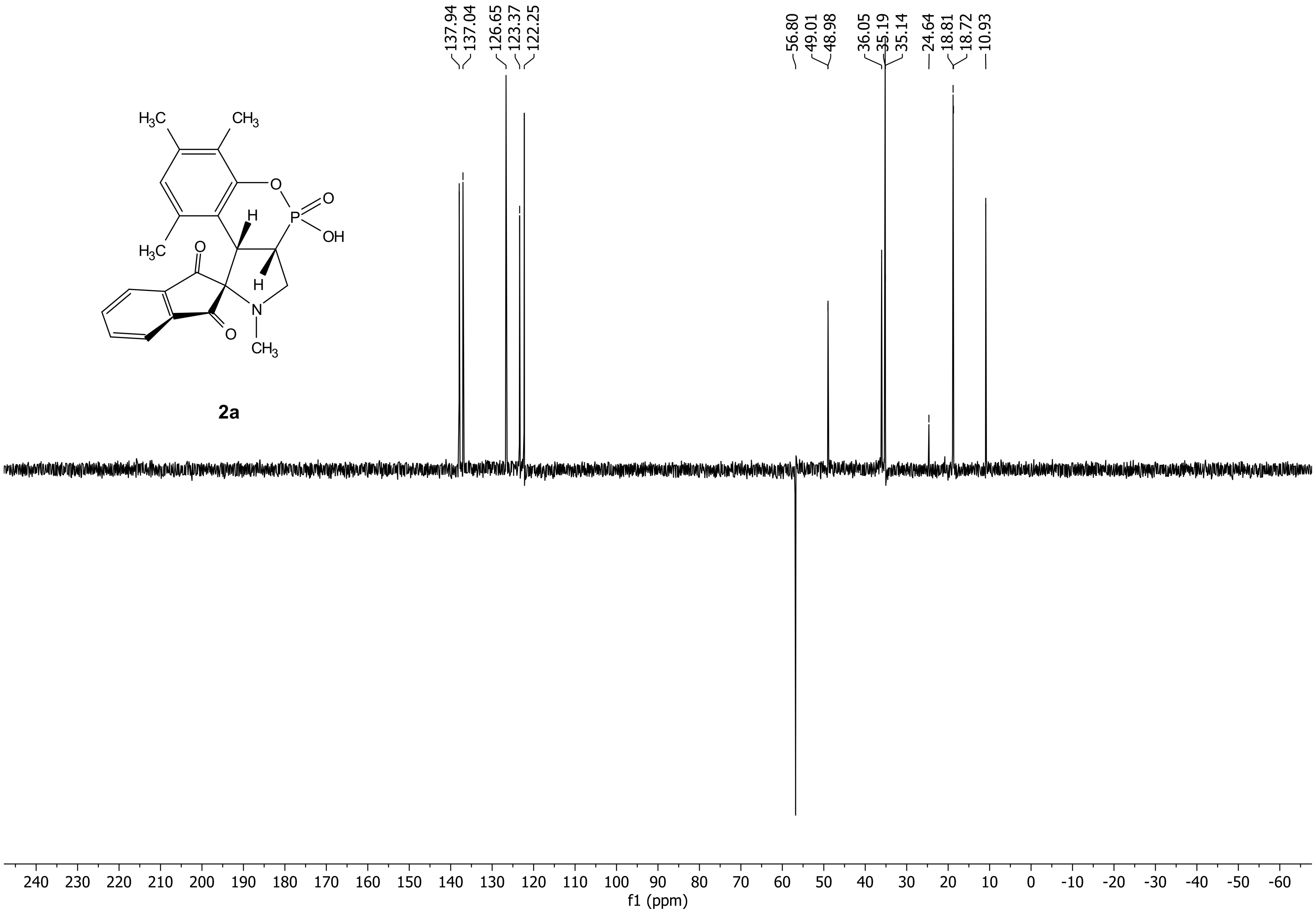

Figure S6. ^{13}C DEPT spectrum (D_2O, 151MHz) of the compound **2a**

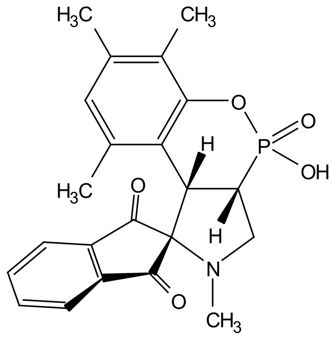

2a

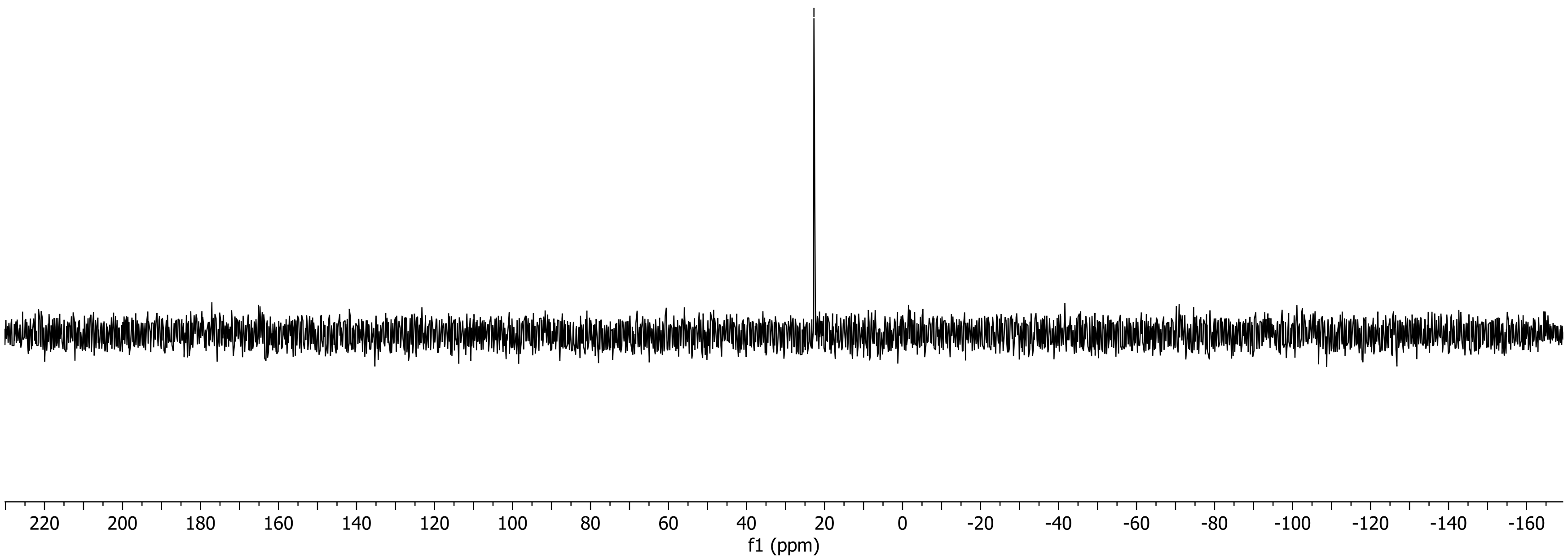

Figure S7. ^{31}P NMR spectrum (D_2O, 243MHz) of the compound **2a**

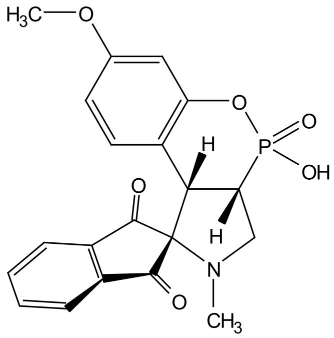

2b

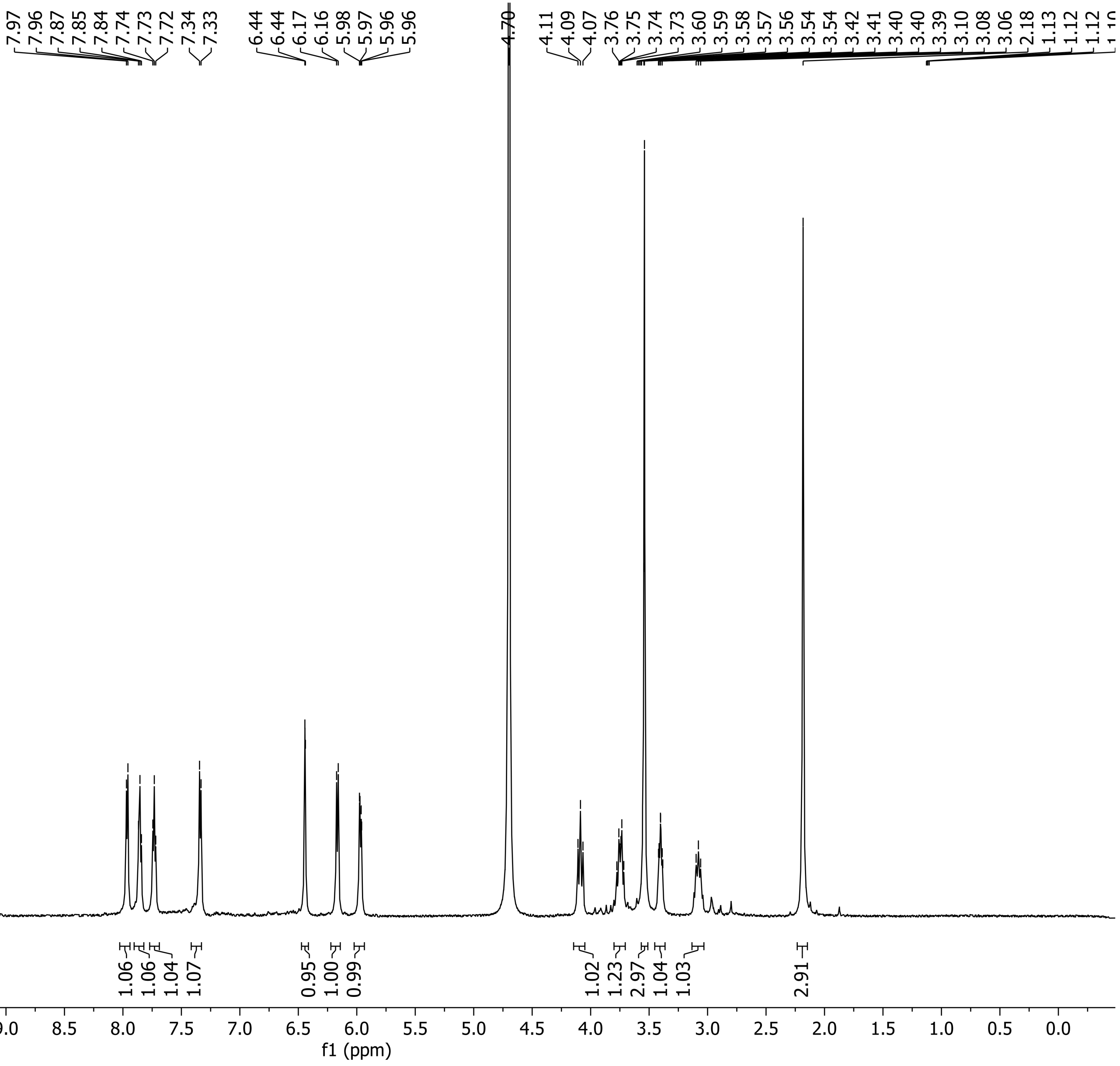

Figure S8. ^{1}H NMR spectrum (D_2O, 600MHz) of the compound **2b**

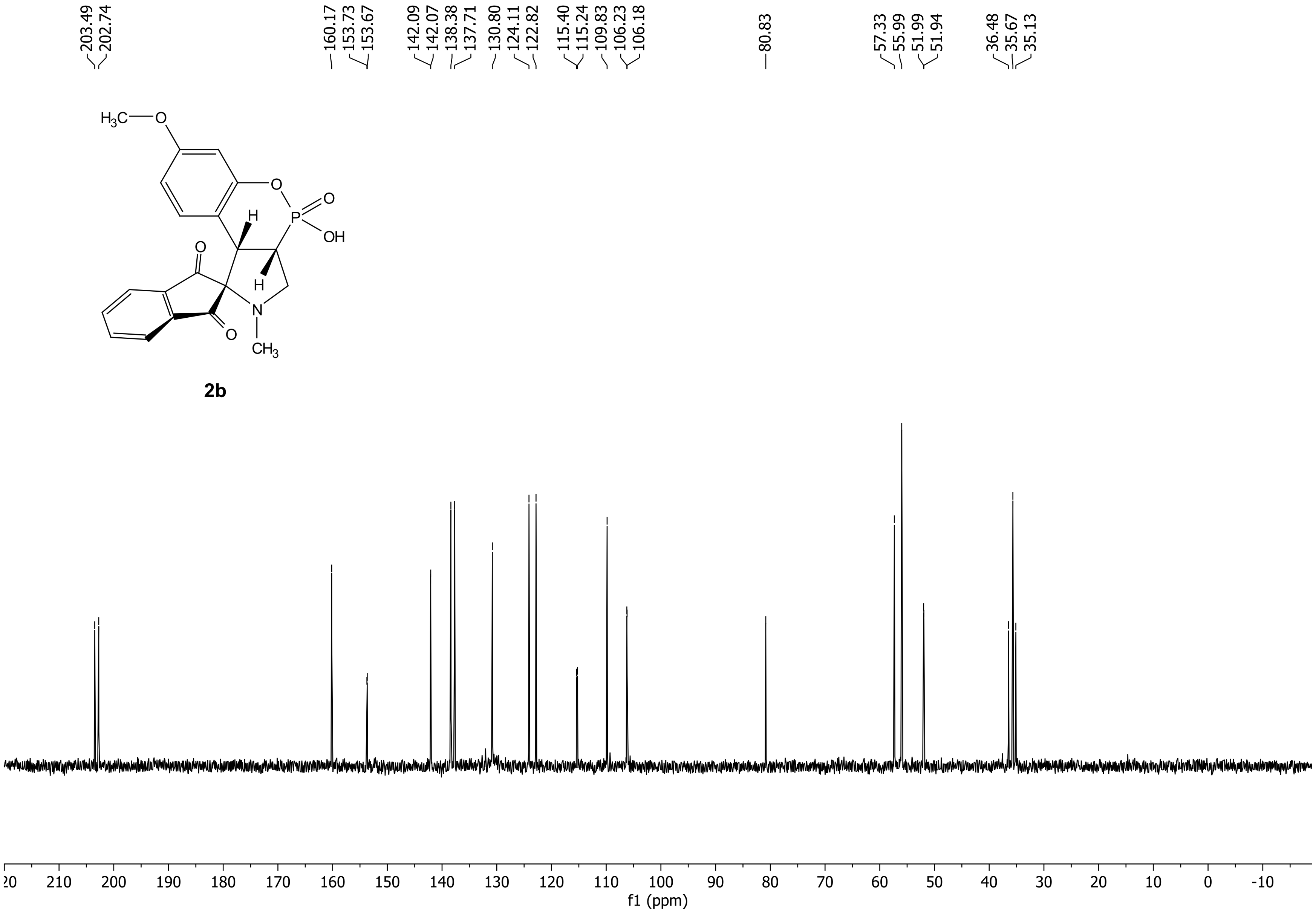

Figure S9. {^{1}H}-^{13}C NMR spectrum (D_2O, 151MHz) of the compound **2b**

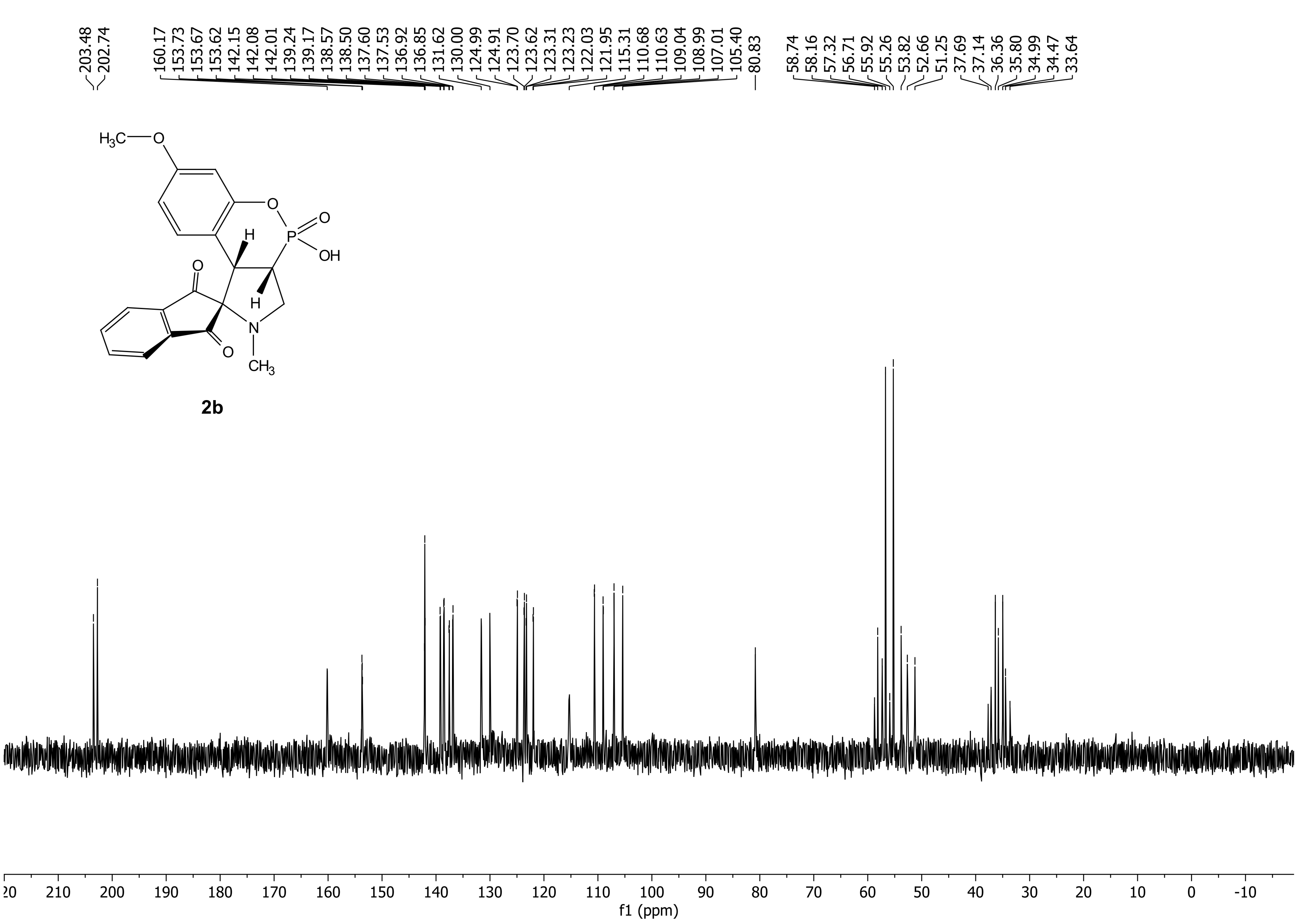

Figure S10. ^{13}C NMR spectrum (D_2O, 151MHz) of the compound **2b**

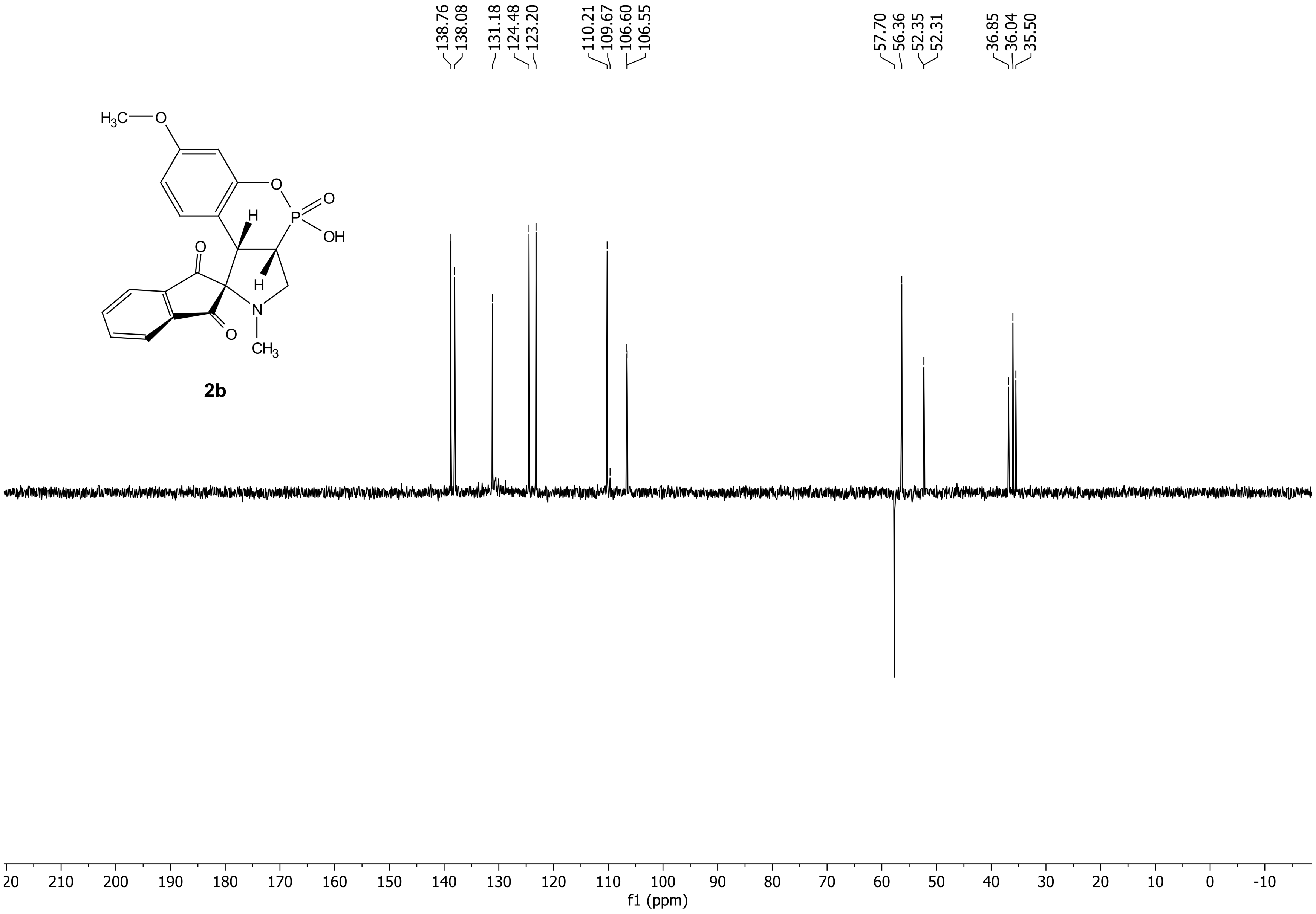

Figure S11. ^{13}C DEPT spectrum (D_2O, 151MHz) of the compound **2b**

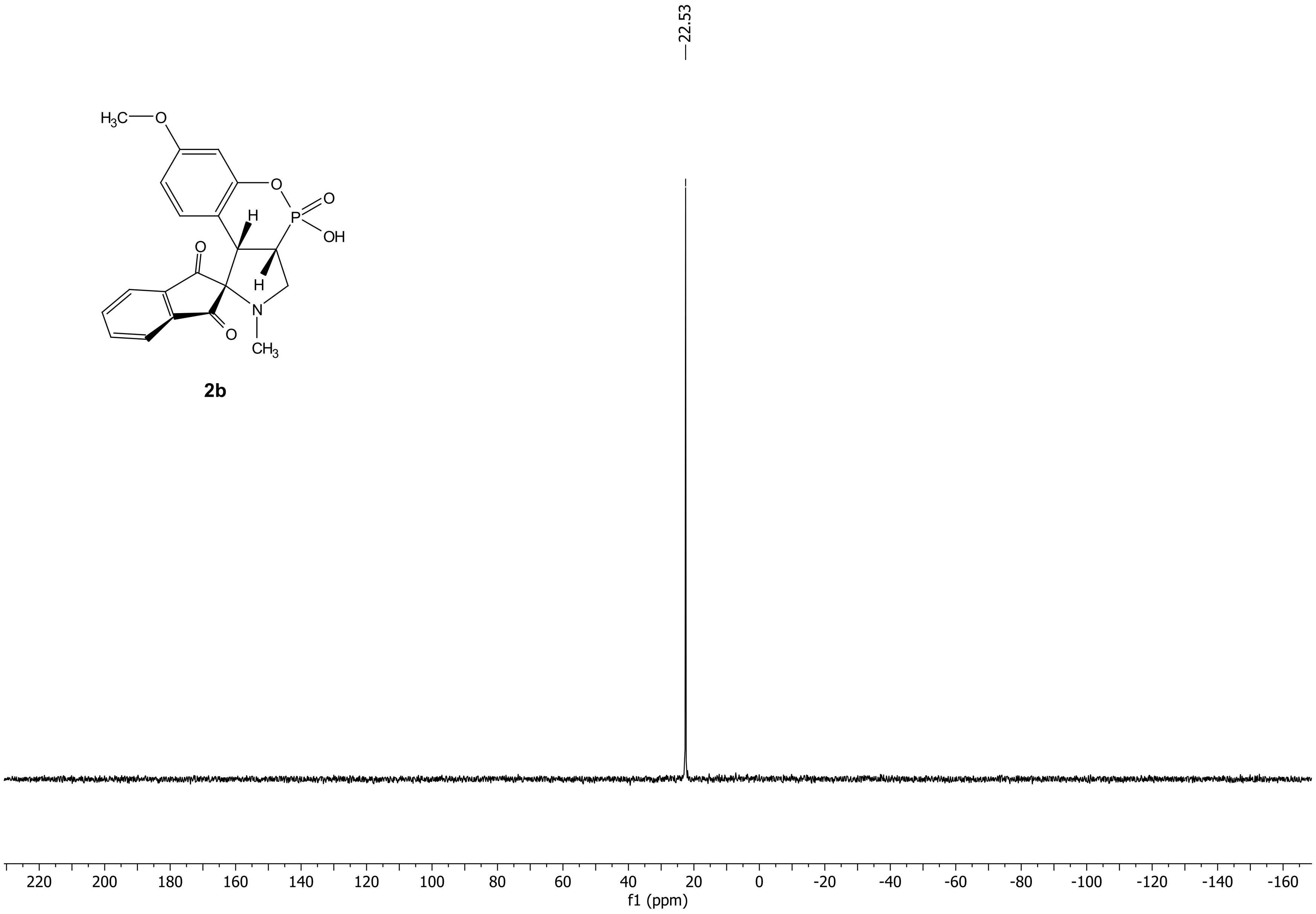

Figure S12. ^{31}P NMR spectrum (D_2O, 243MHz) of the compound **2b**

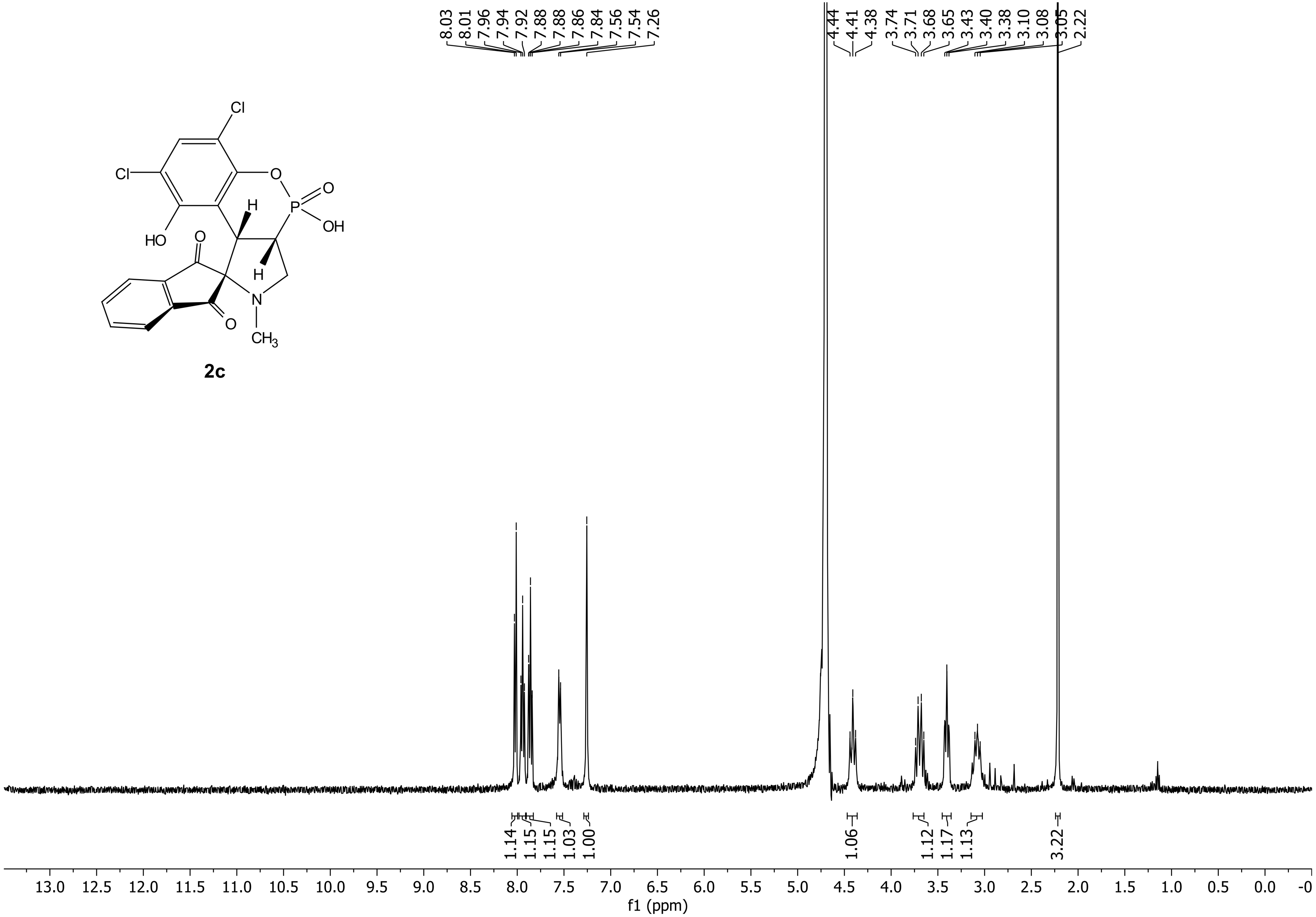

Figure S13. ^{1}H NMR spectrum (D_2O, 600MHz) of the compound **2c**

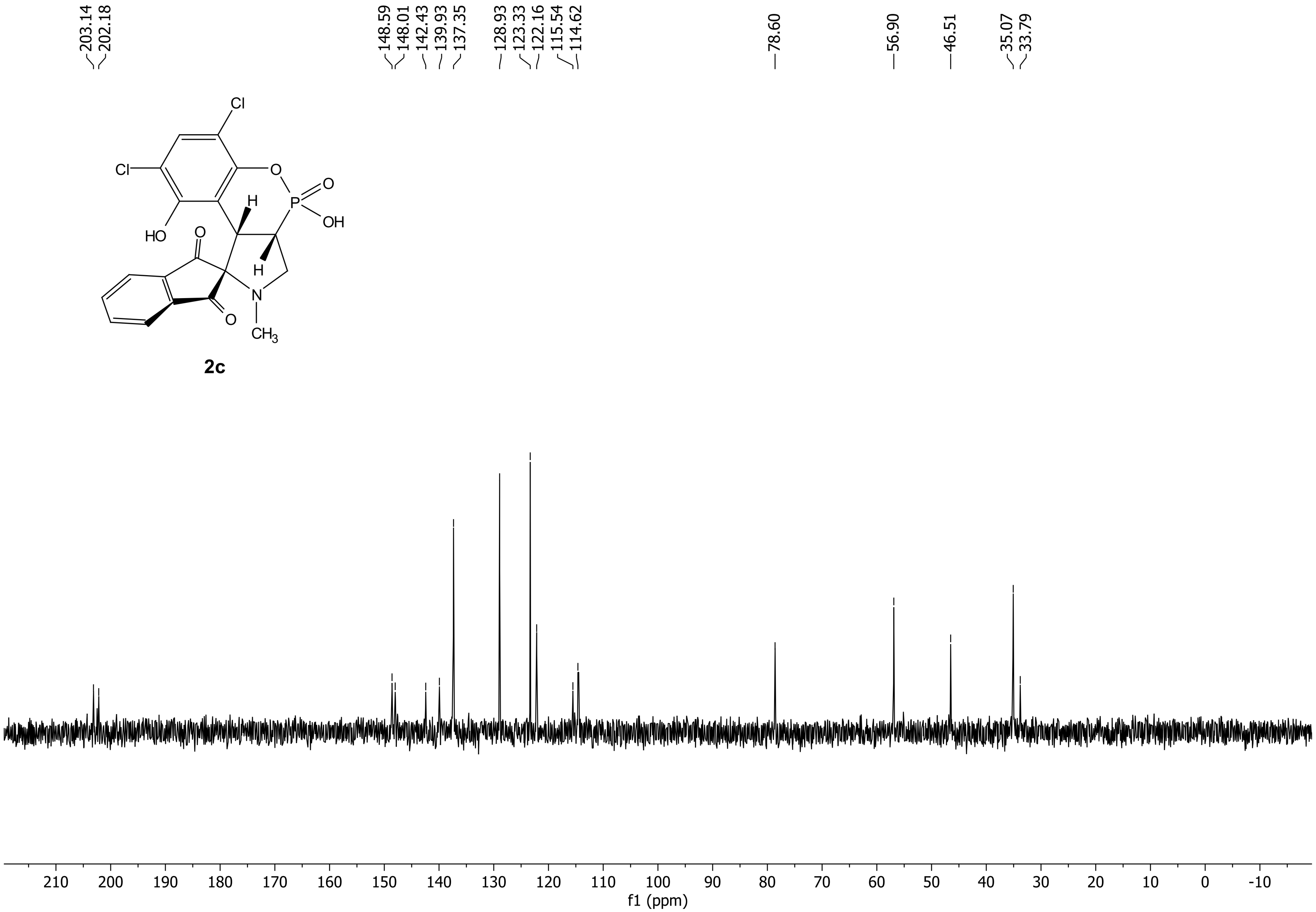

Figure S14. {^{1}H}-^{13}C NMR spectrum (D_2O, 151MHz) of the compound **2c**

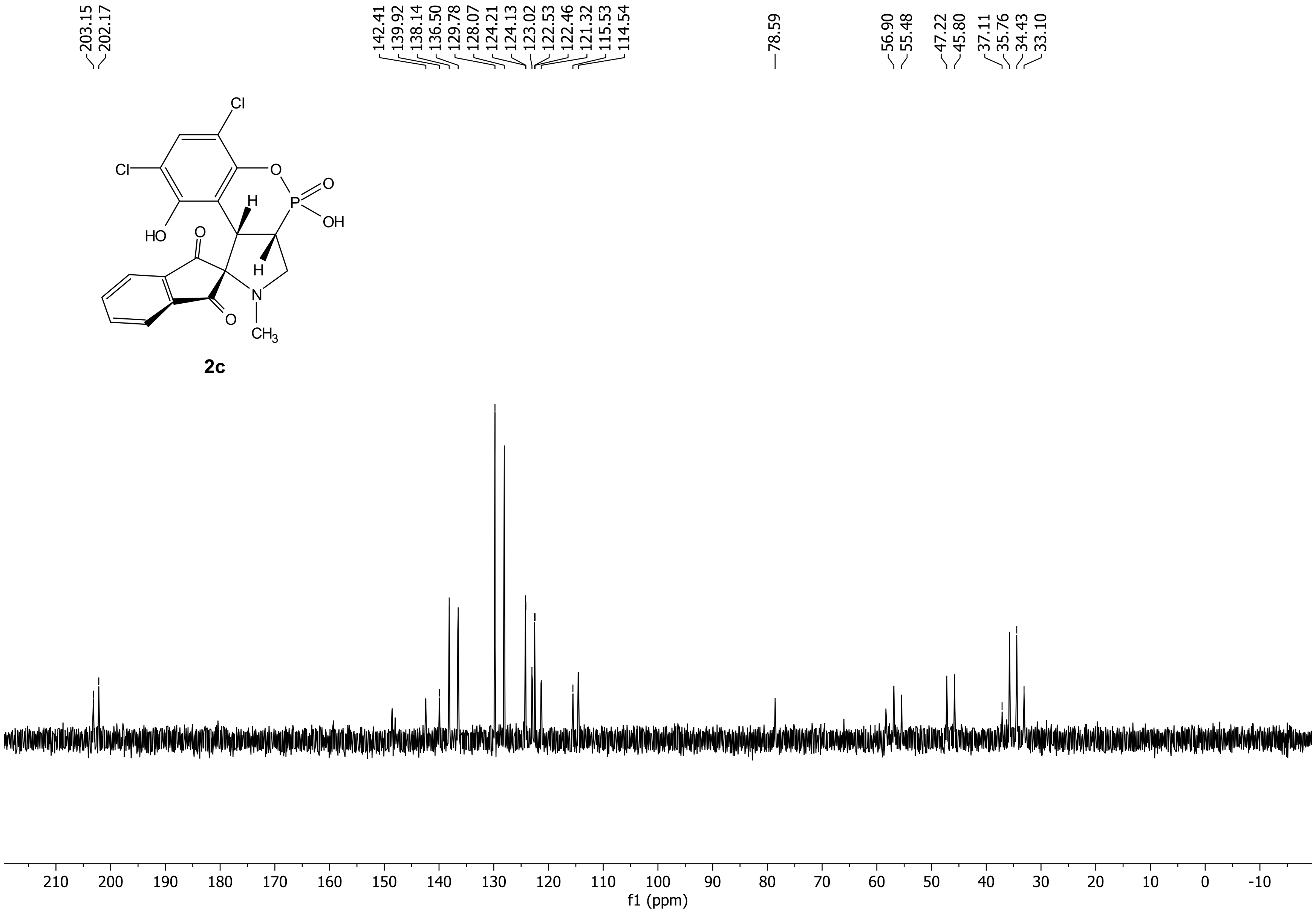

Figure S15. ^{13}C NMR spectrum (D_2O, 151MHz) of the compound **2c**

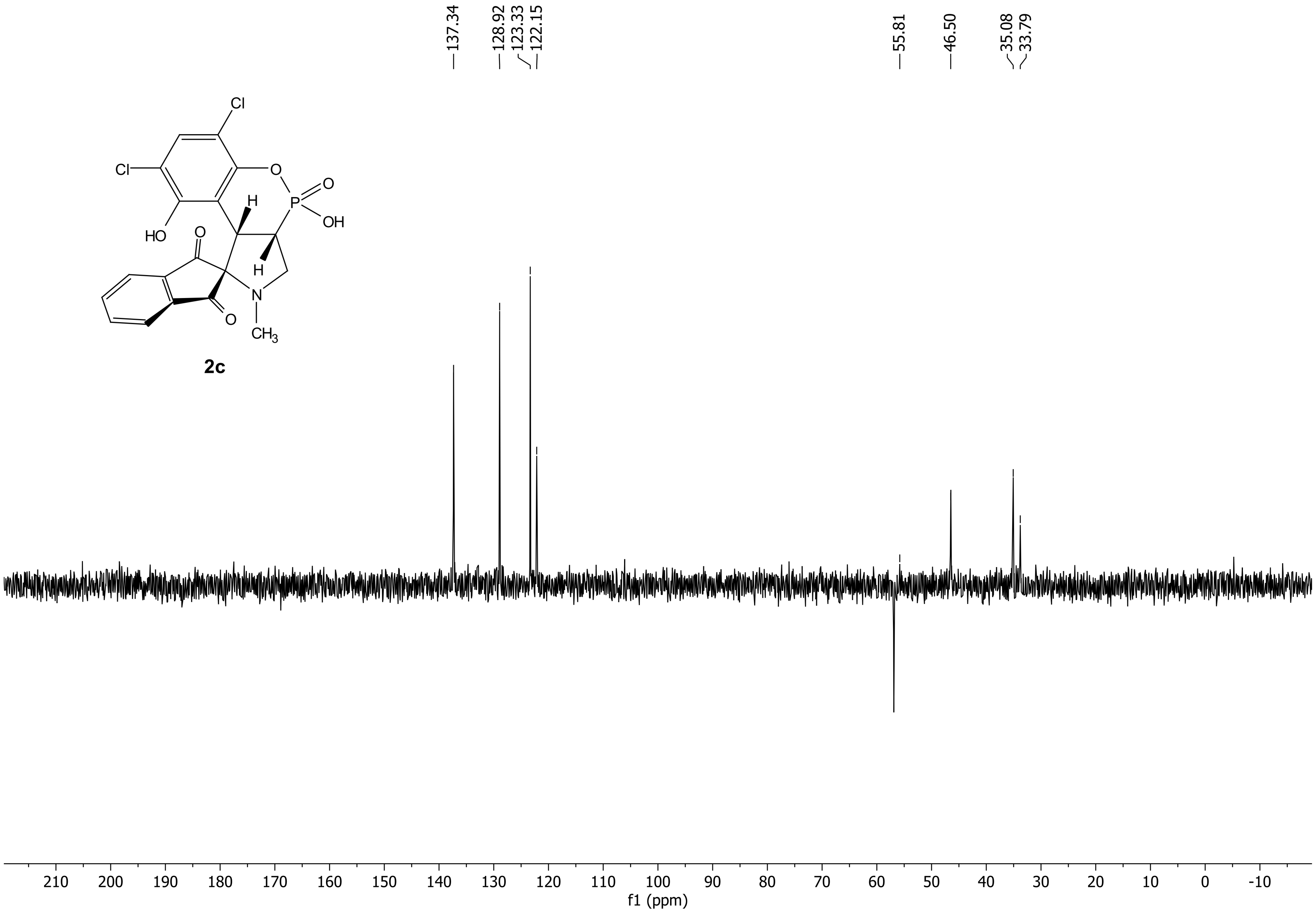

Figure S16. ^{13}C DEPT spectrum (D_2O, 151MHz) of the compound **2c**

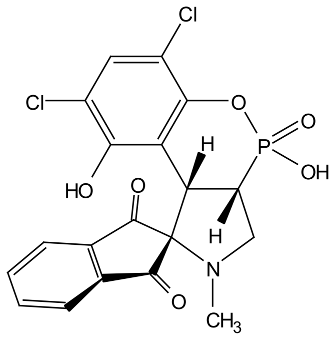

2c

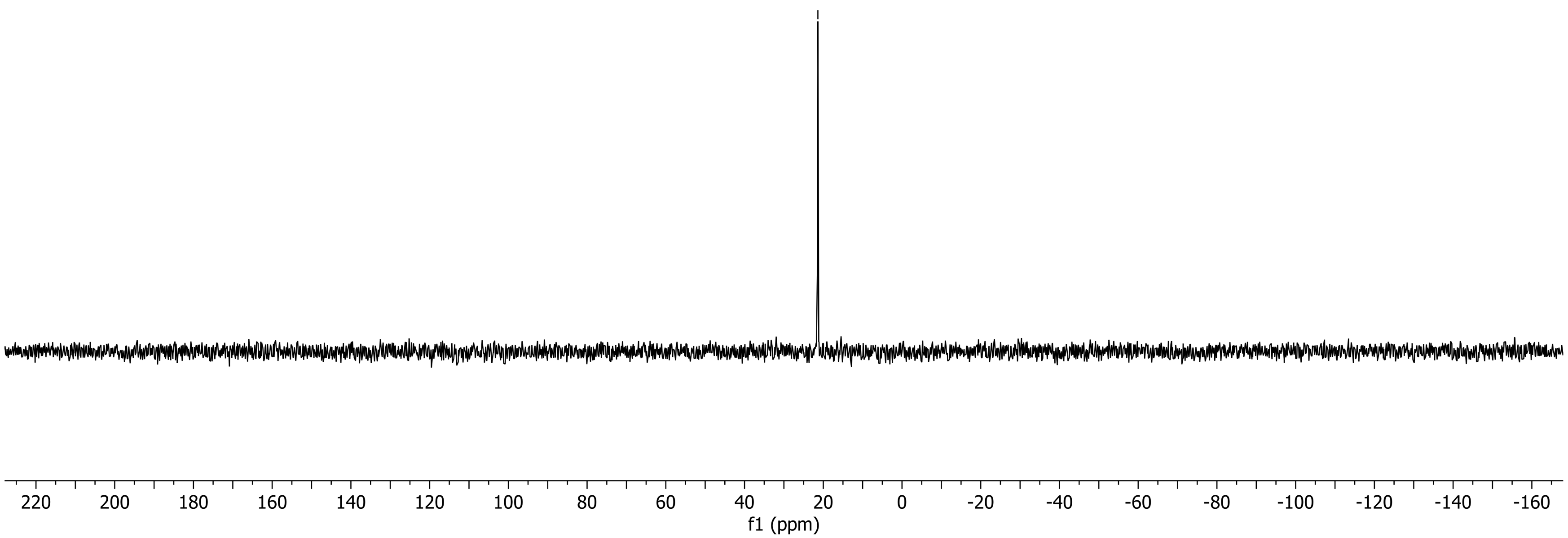

Figure S17. ^{31}P NMR spectrum (D_2O, 243MHz) of the compound **2c**

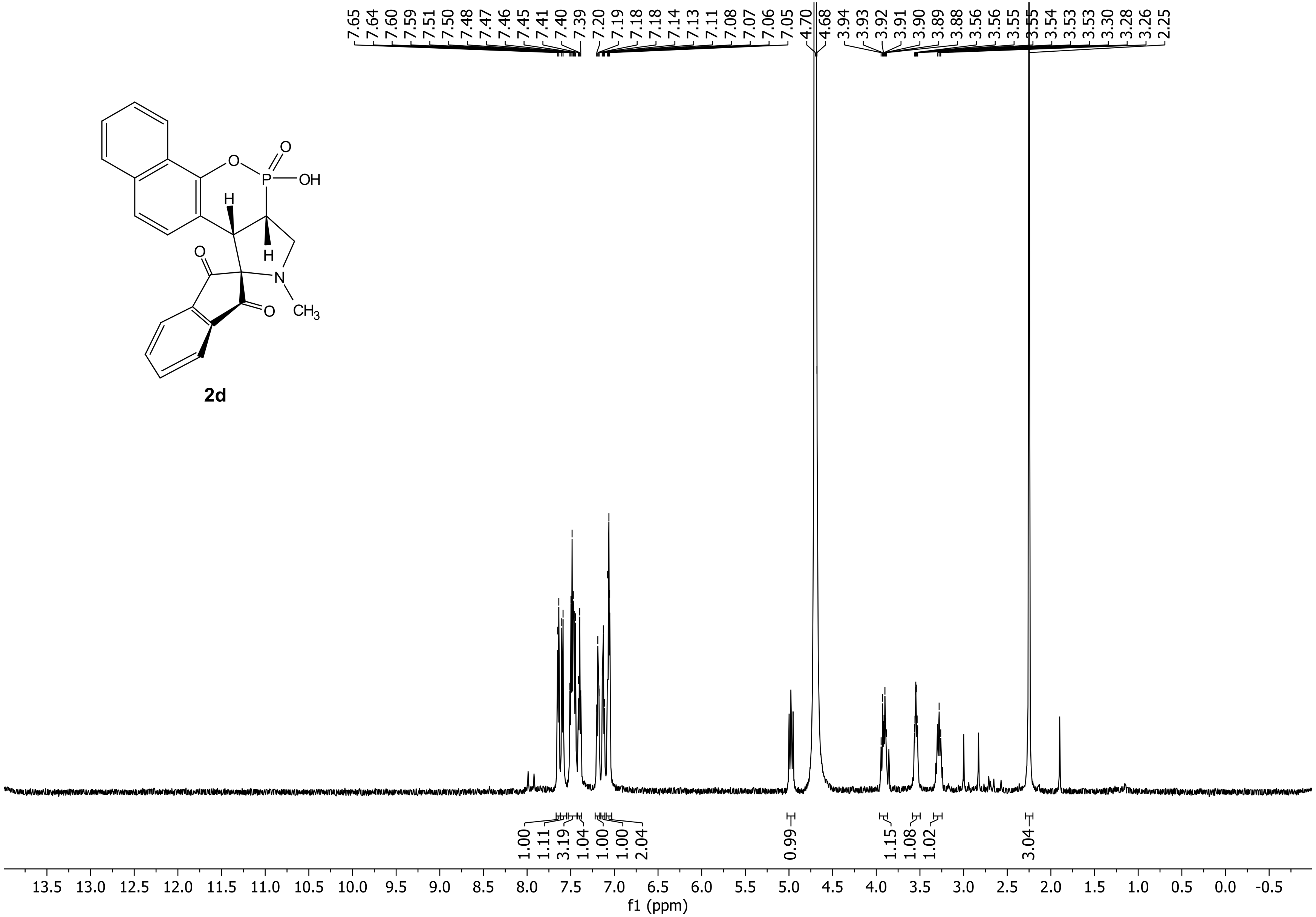

Figure S18. ^{1}H NMR spectrum (D_2O, 600MHz) of the compound **2d**

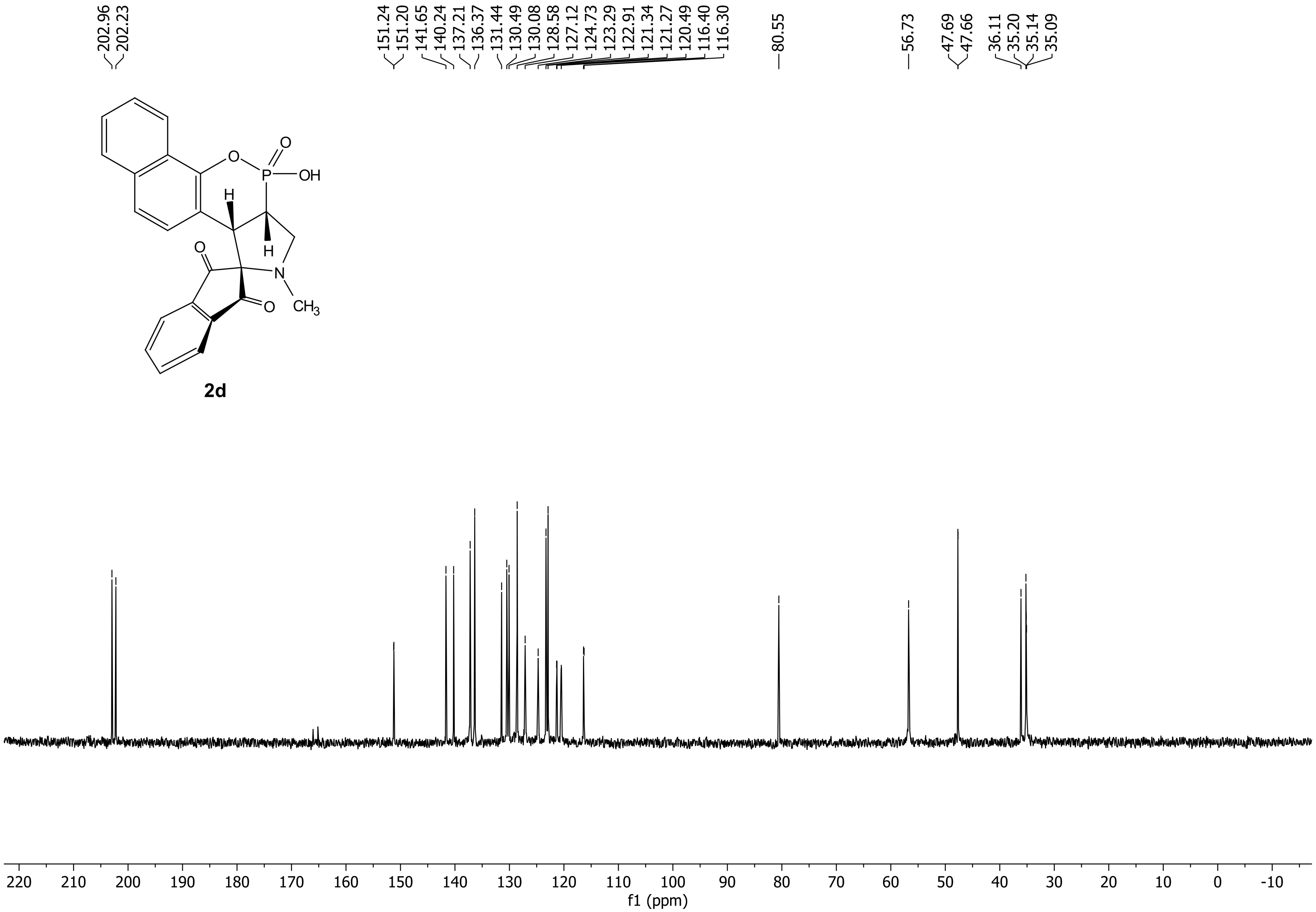

Figure S19. {^{1}H}-^{13}C NMR spectrum (D_2O, 151MHz) of the compound **2d**

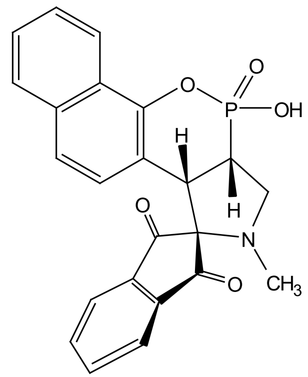

2d

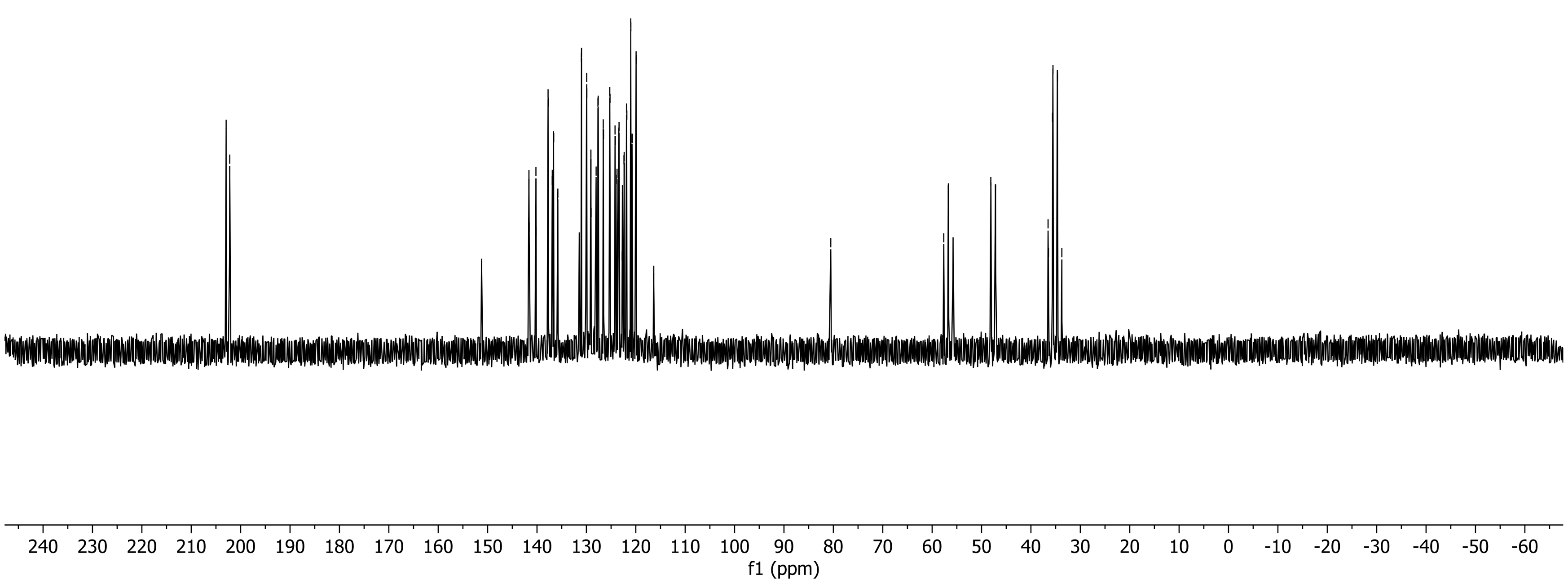

Figure S20. ^{13}C NMR spectrum (D_2O, 151MHz) of the compound **2d**

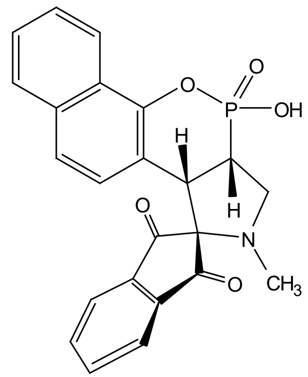

2d

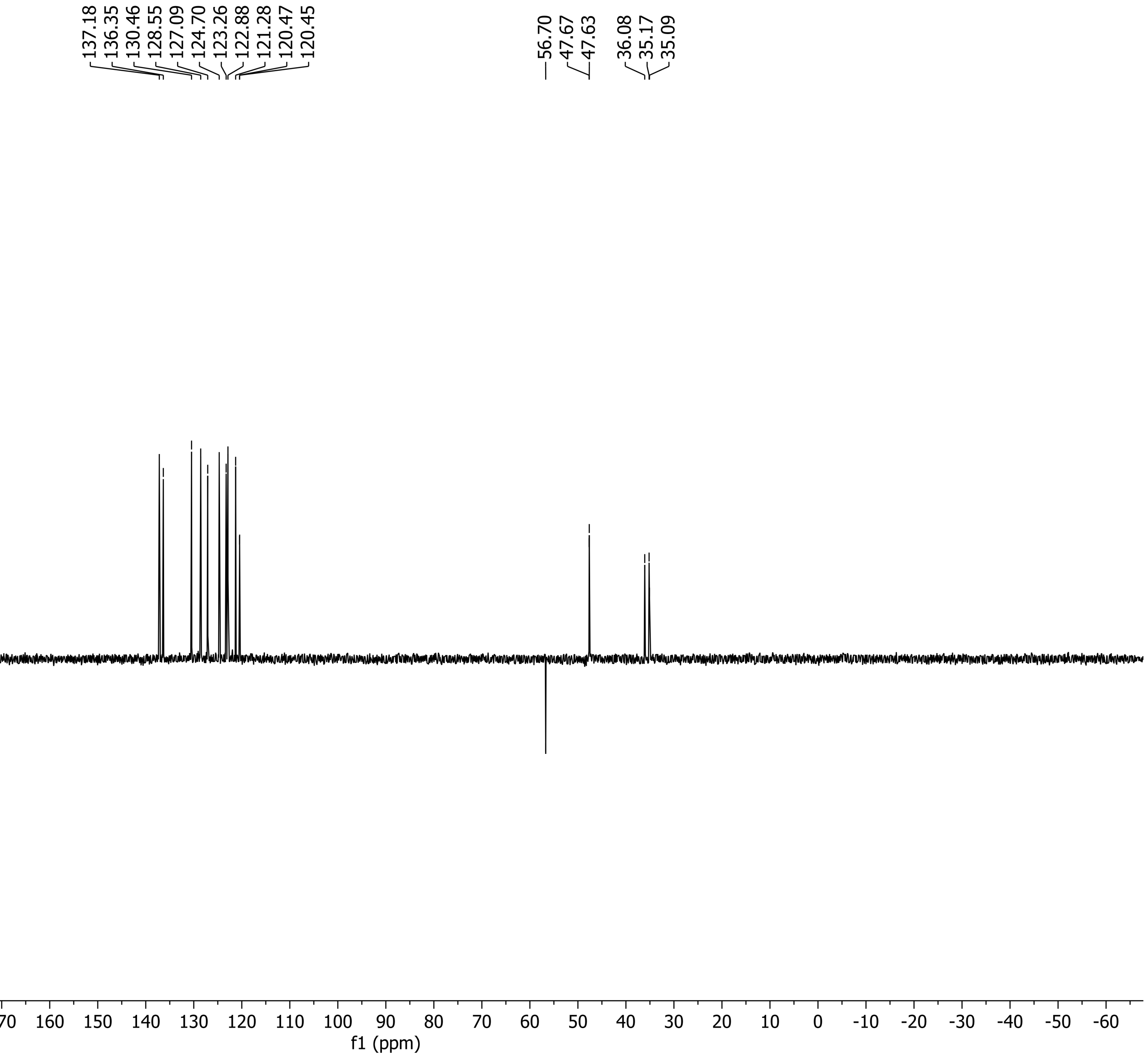

Figure S21. ^{13}C DEPT spectrum (D_2O, 151MHz) of the compound **2d**

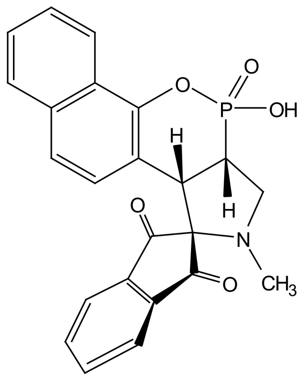

2d

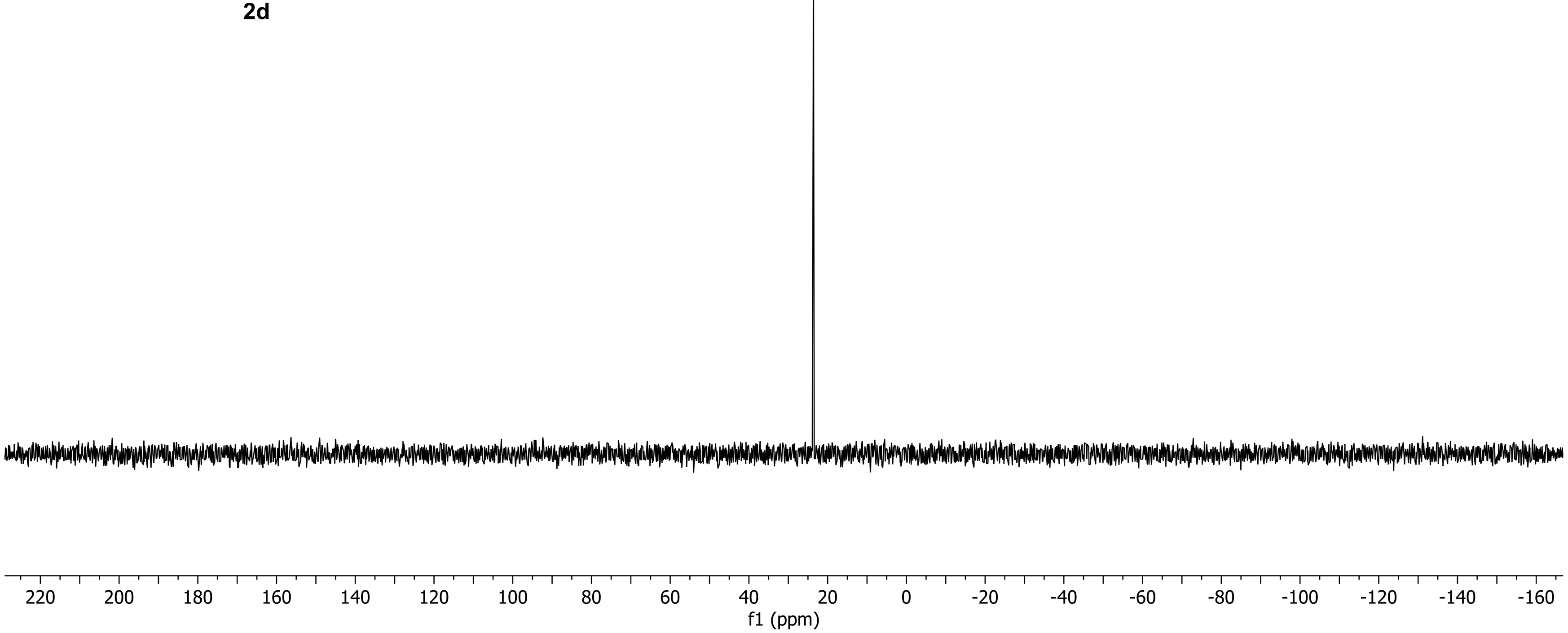

Figure S22. ^{31}P NMR spectrum (D_2O, 243MHz) of the compound **2d**

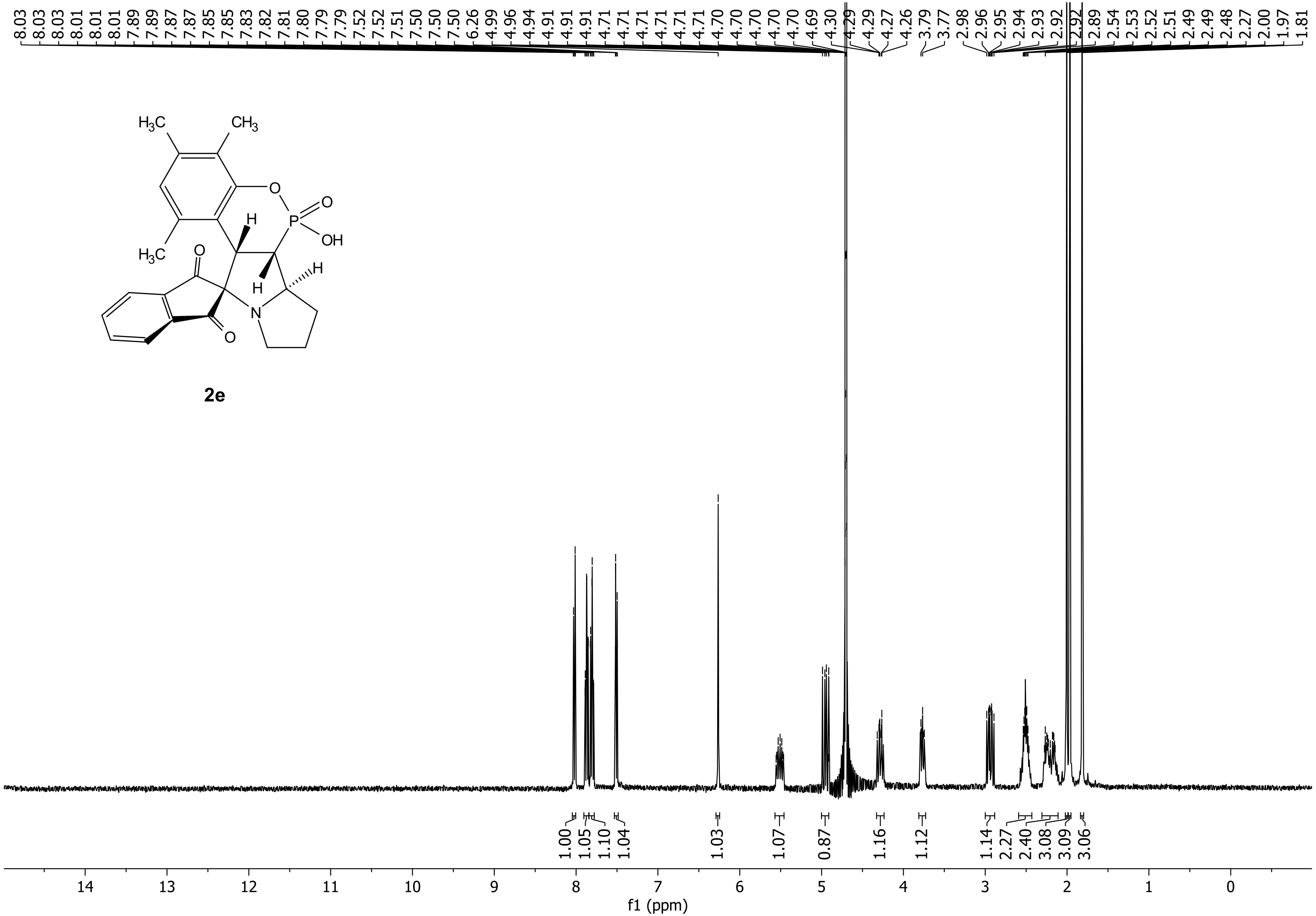

Figure S23. ^{1}H NMR spectrum (D_2O, 600MHz) of the compound **2e**

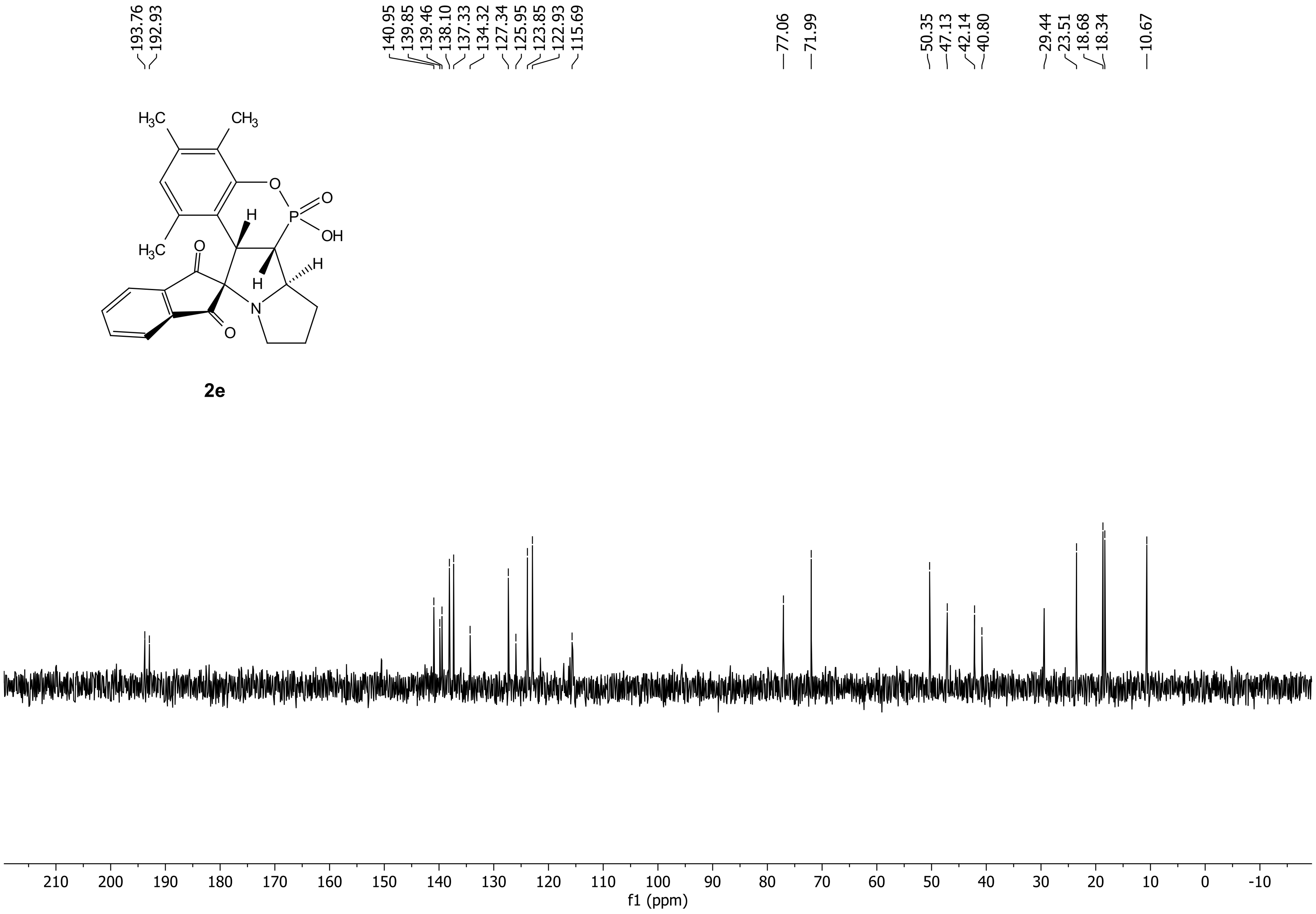

Figure S24. {^{1}H}-^{13}C NMR spectrum (D_2O, 151MHz) of the compound **2e**

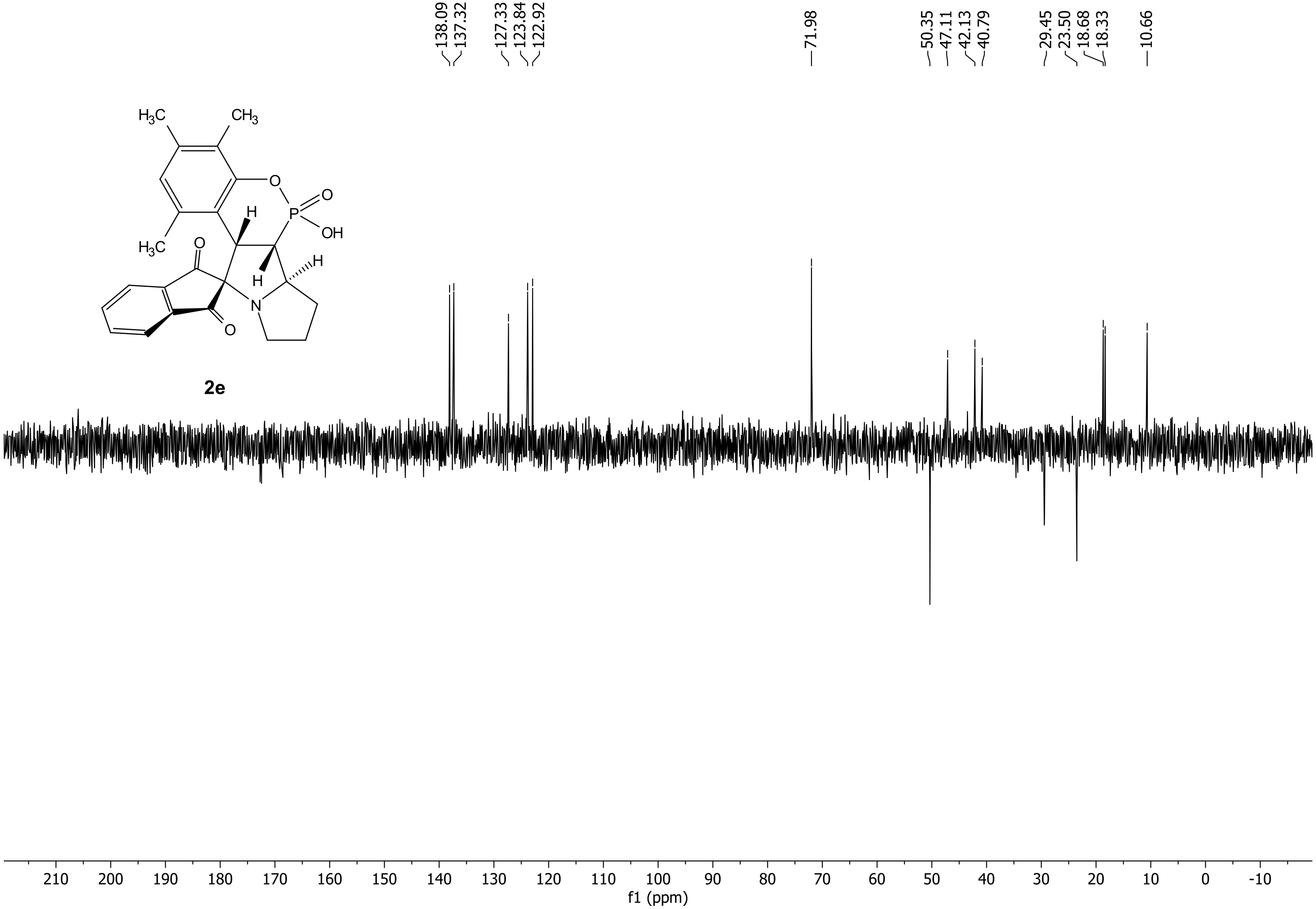

Figure S25. ^{13}C DEPT spectrum (D_2O, 151MHz) of the compound **2e**

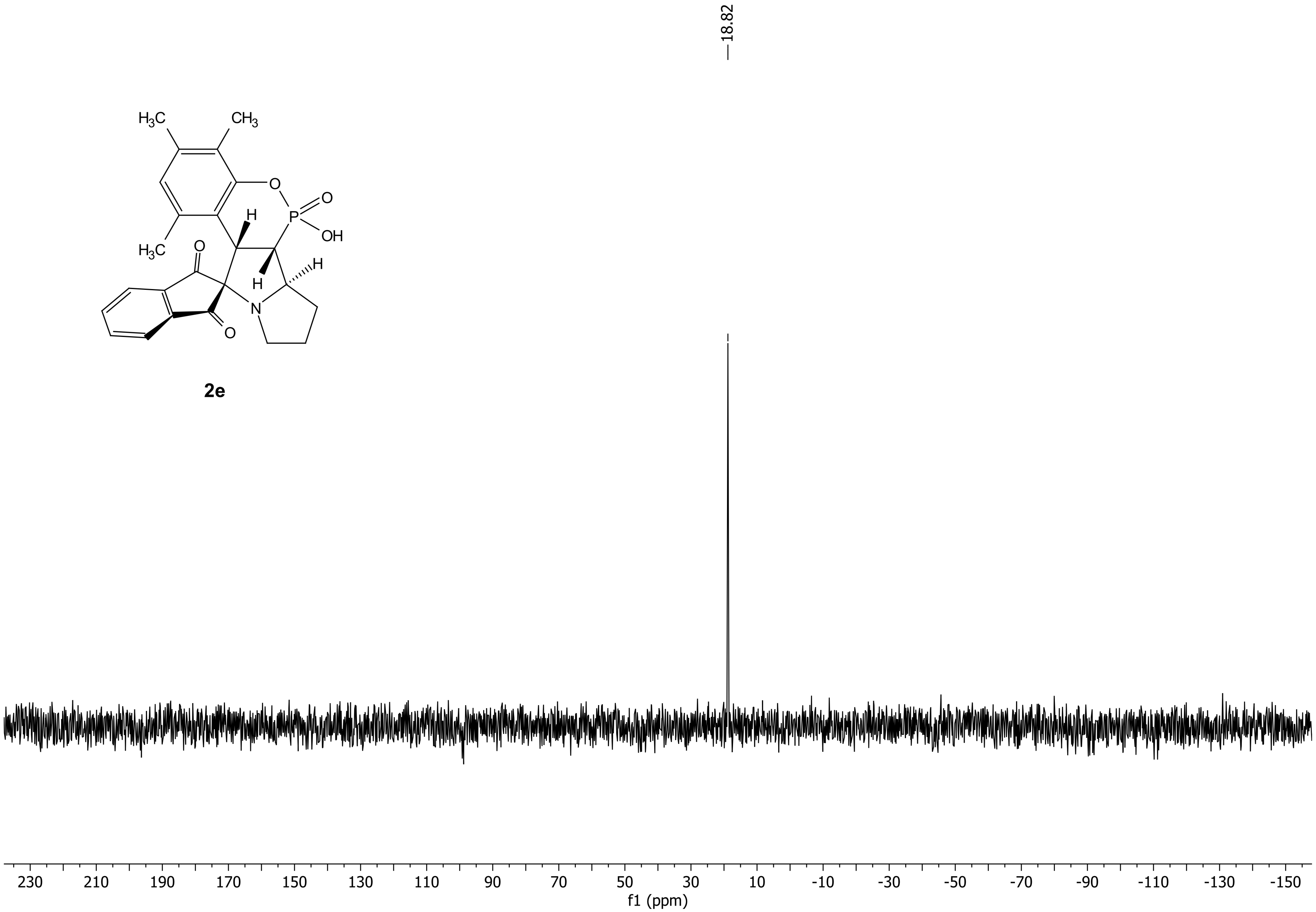

Figure S26. ^{31}P NMR spectrum (D_2O, 243MHz) of the compound **2e**

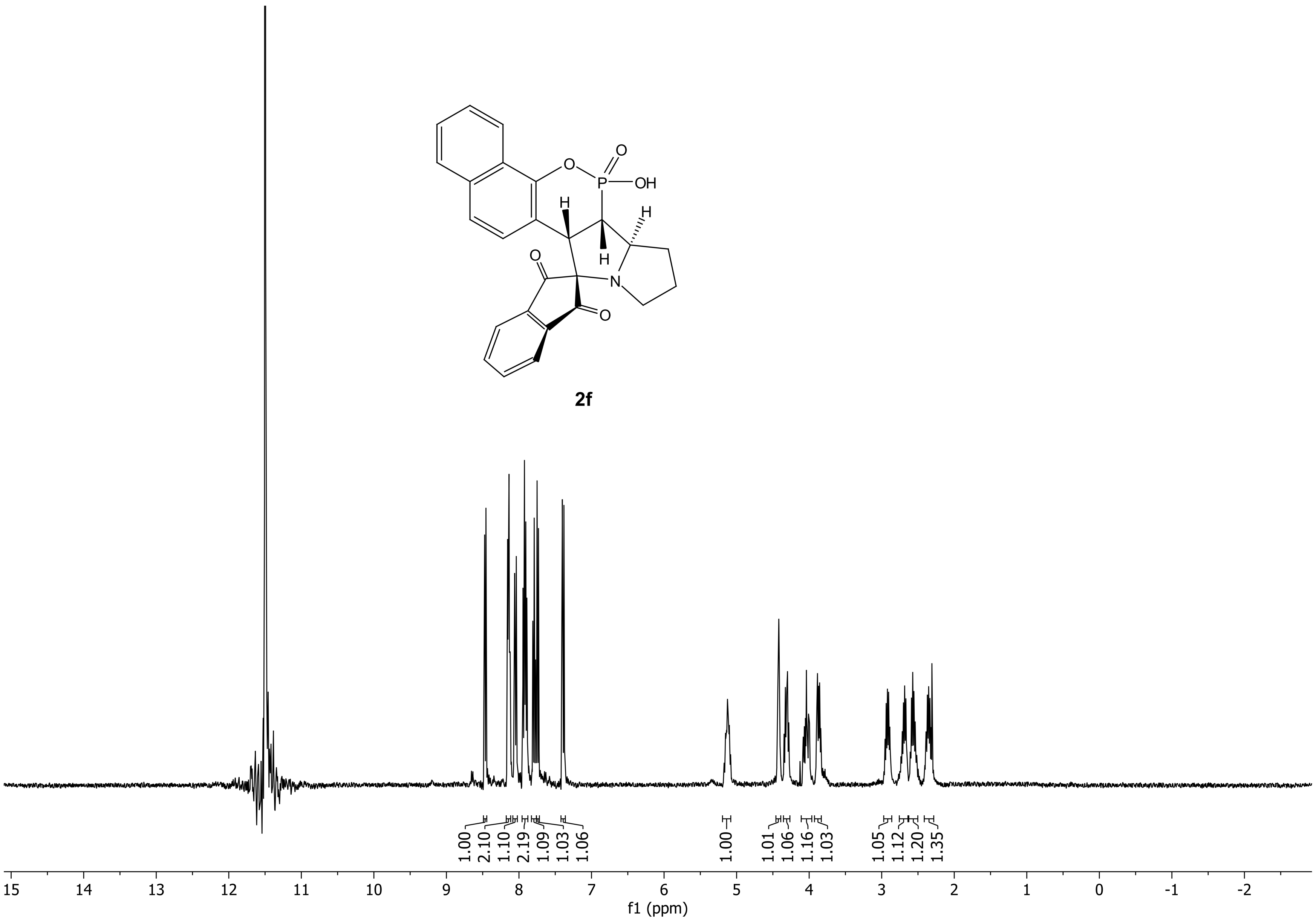

Figure S27. ^{1}H NMR spectrum (CD_3CO_2D, 600MHz) of the compound **2f**

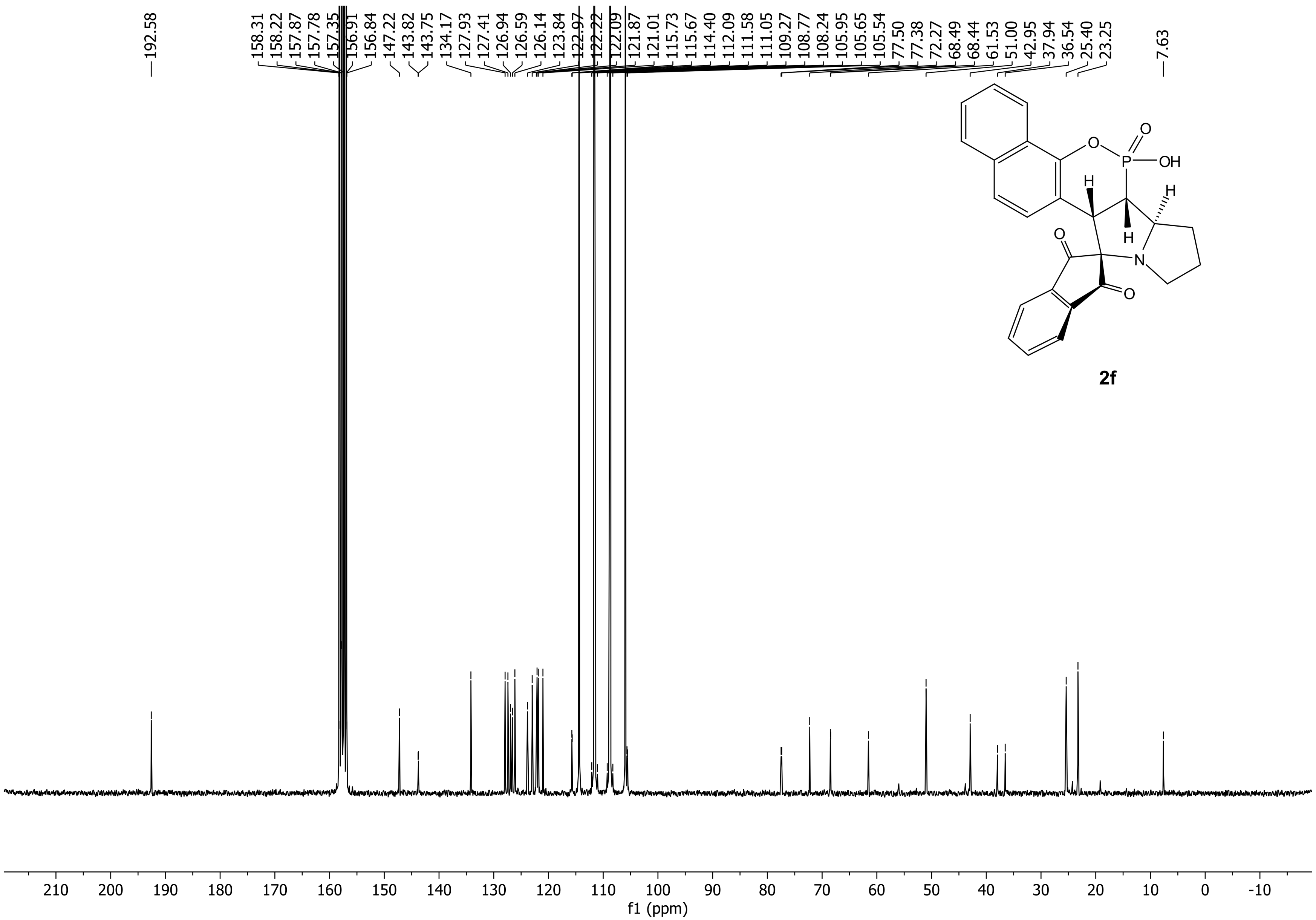

Figure S28. {^{1}H}-^{13}C NMR spectrum (CD_3CO_2D, 151MHz) of the compound **2f**

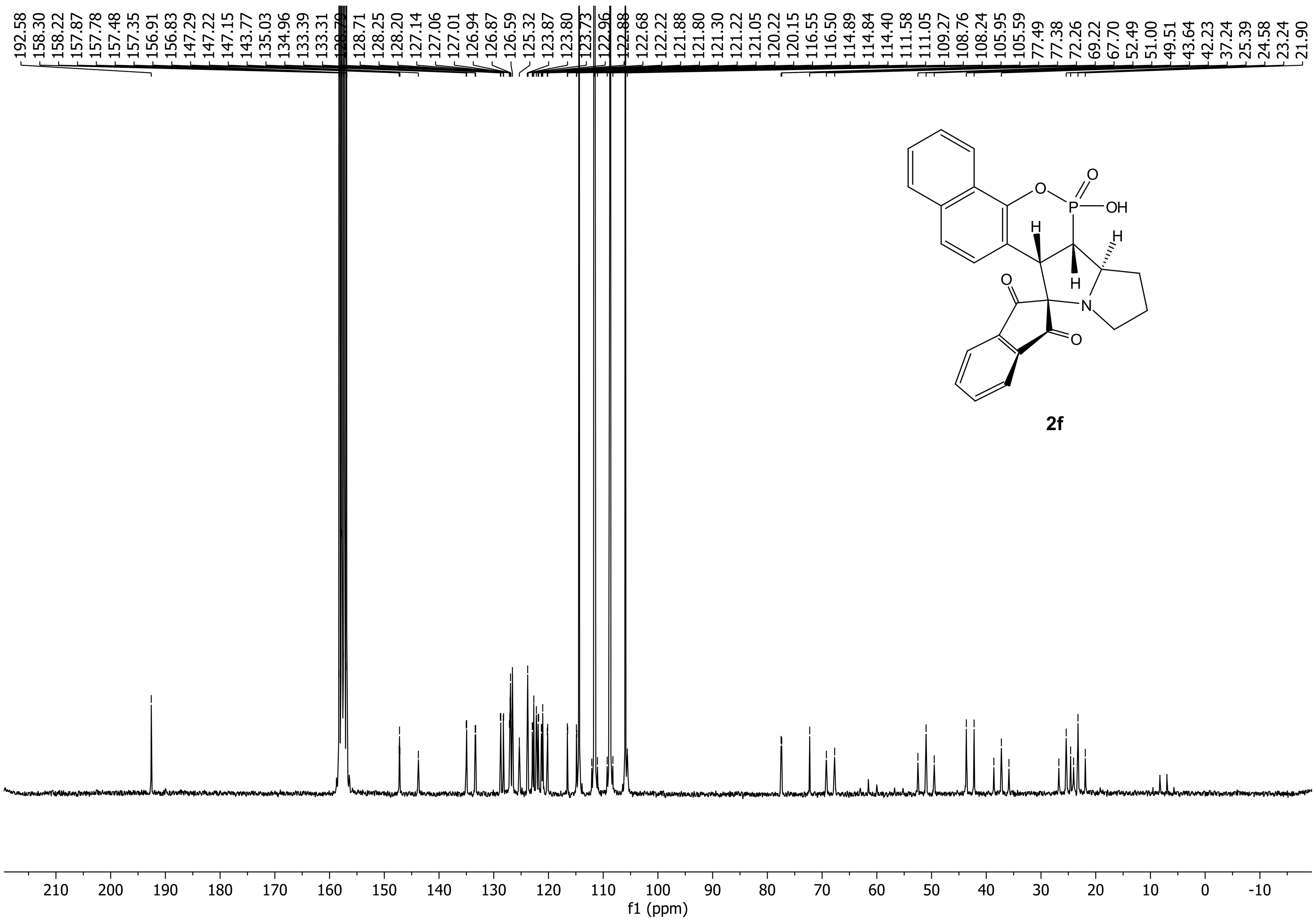

Figure S29. ^{13}C NMR spectrum (CD_3CO_2D, 151MHz) of the compound **2f**

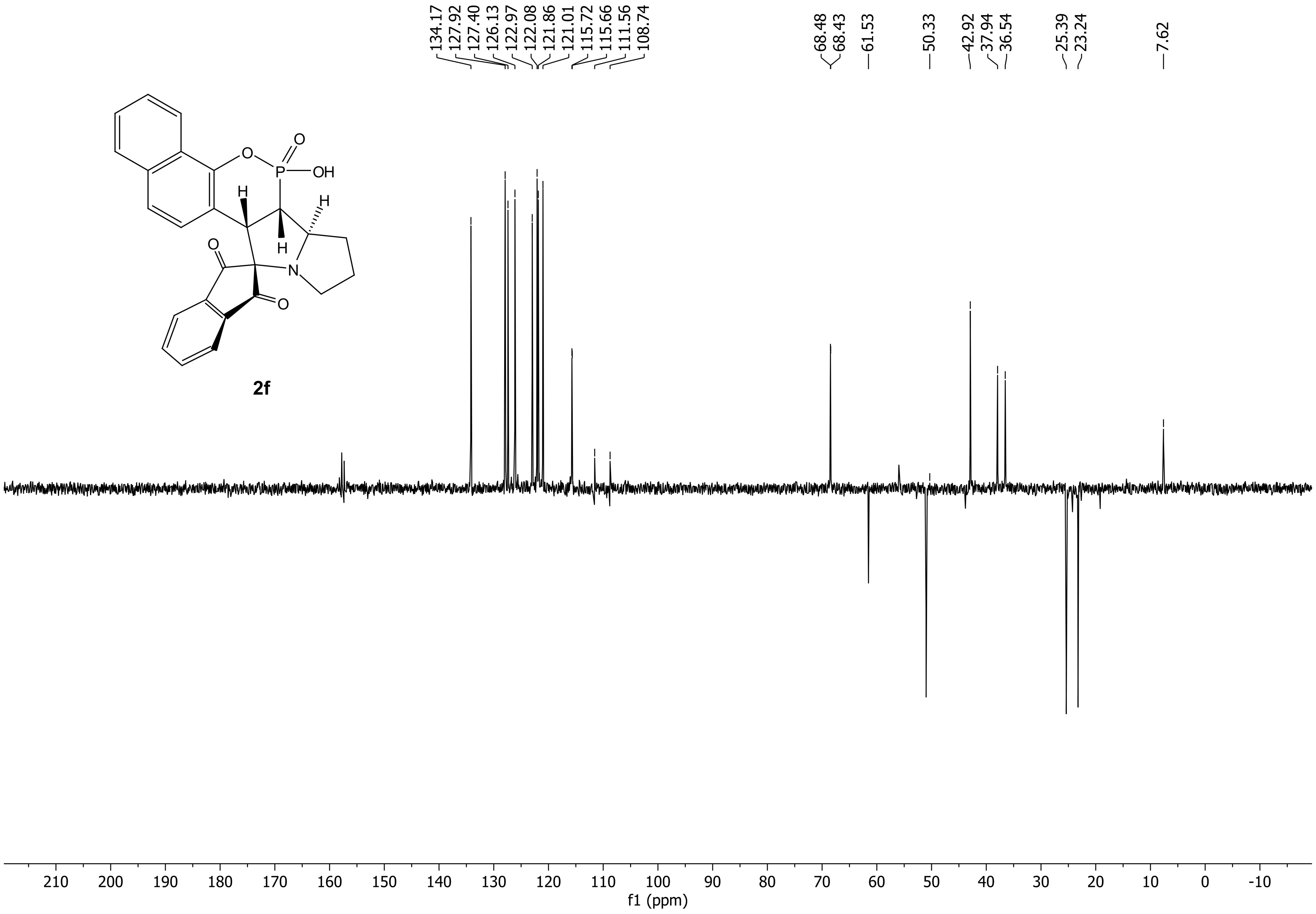

Figure S30. ^{13}C DEPT spectrum (CD_3CO_2D, 151MHz) of the compound **2f**

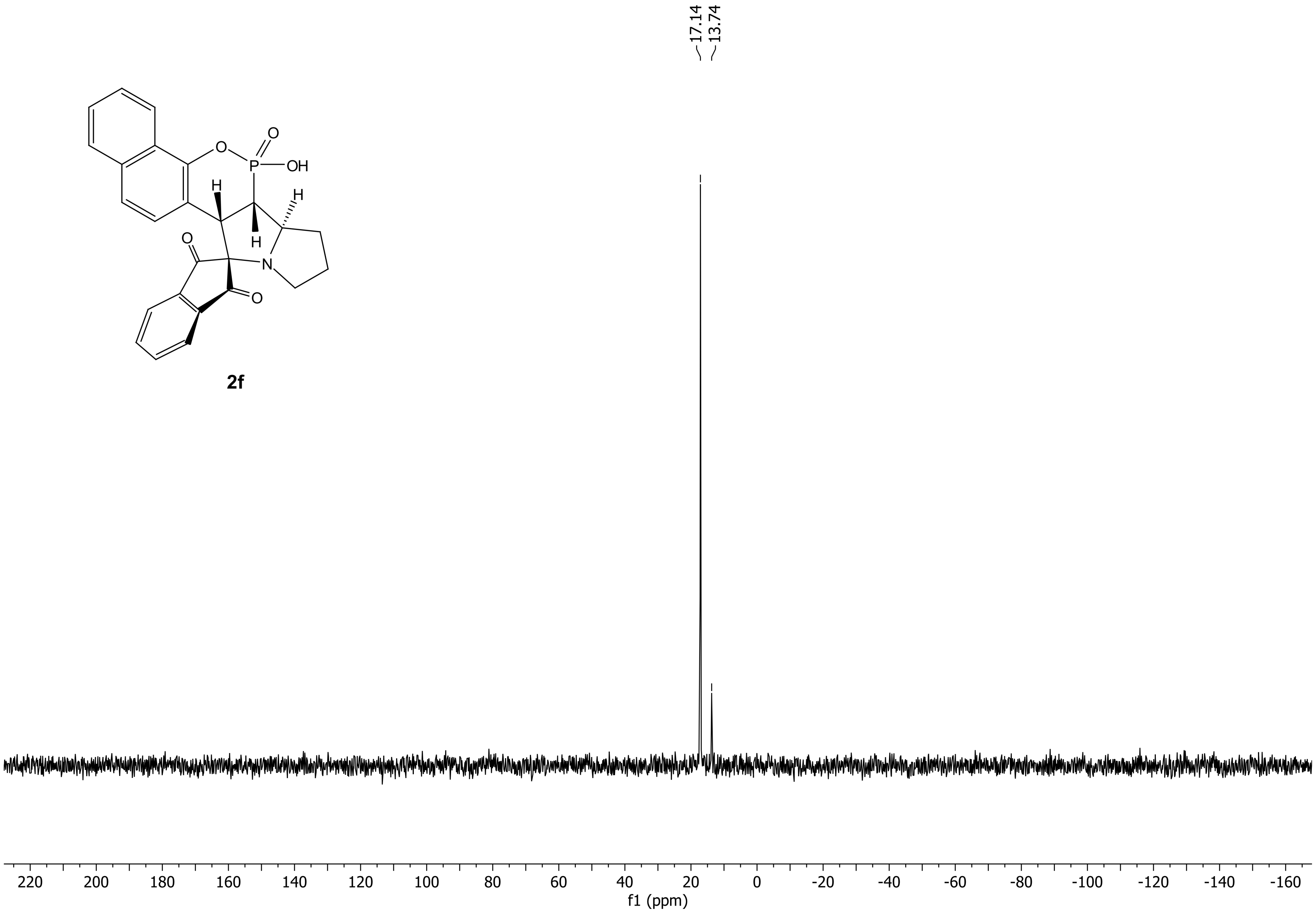

Figure S31. ^{31}P NMR spectrum (CD_3CO_2D, 243MHz) of the compound **2f**

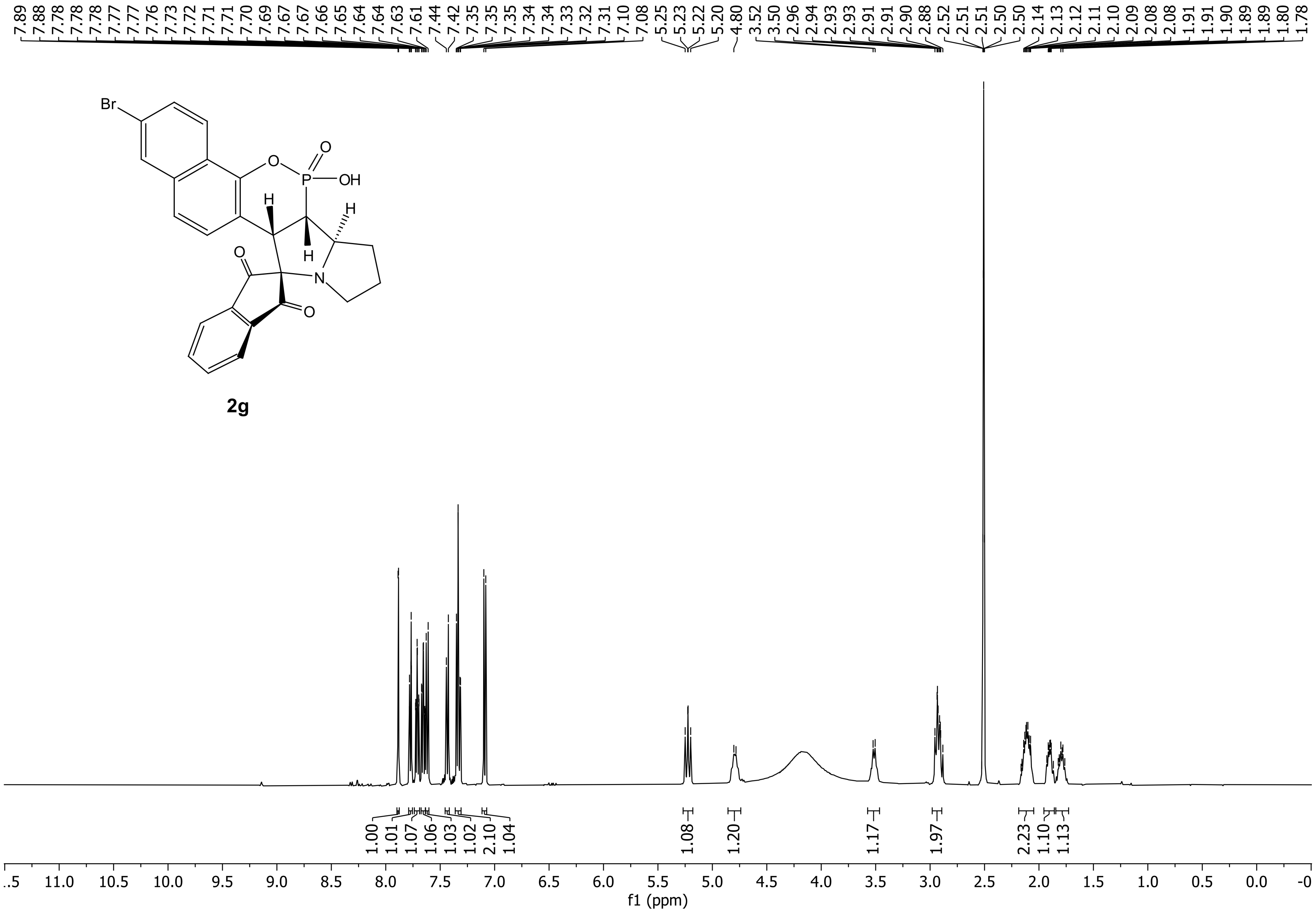

Figure S32. ^{1}H NMR spectrum ($(CD_3)_2SO$, 600MHz) of the compound **2g**

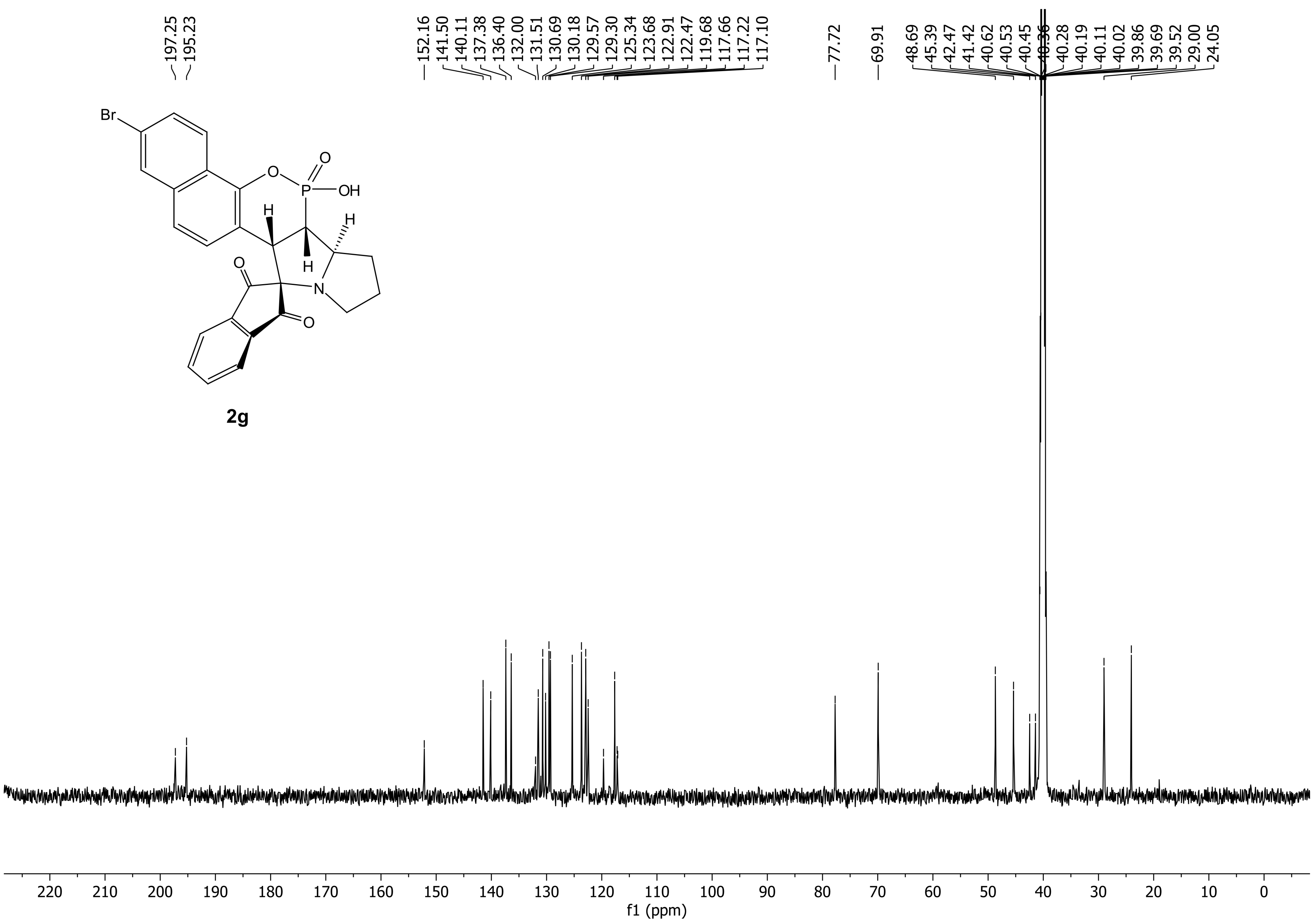

Figure S33. {^{1}H}-^{13}C NMR spectrum ($(CD_3)_2SO$, 151MHz) of the compound **2g**

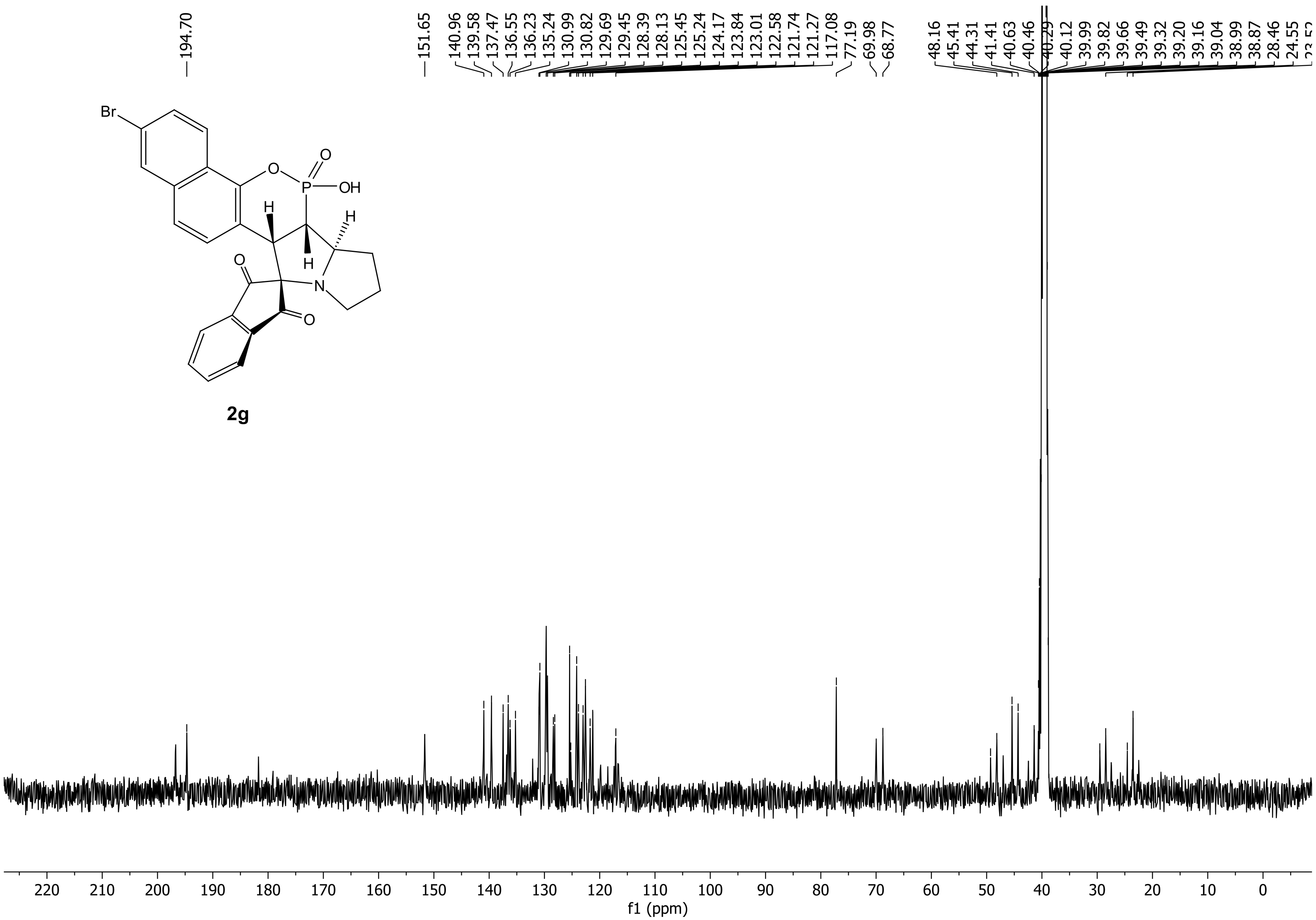

Figure S34. ^{13}C NMR spectrum ($(CD_3)_2SO$, 151MHz) of the compound **2g**

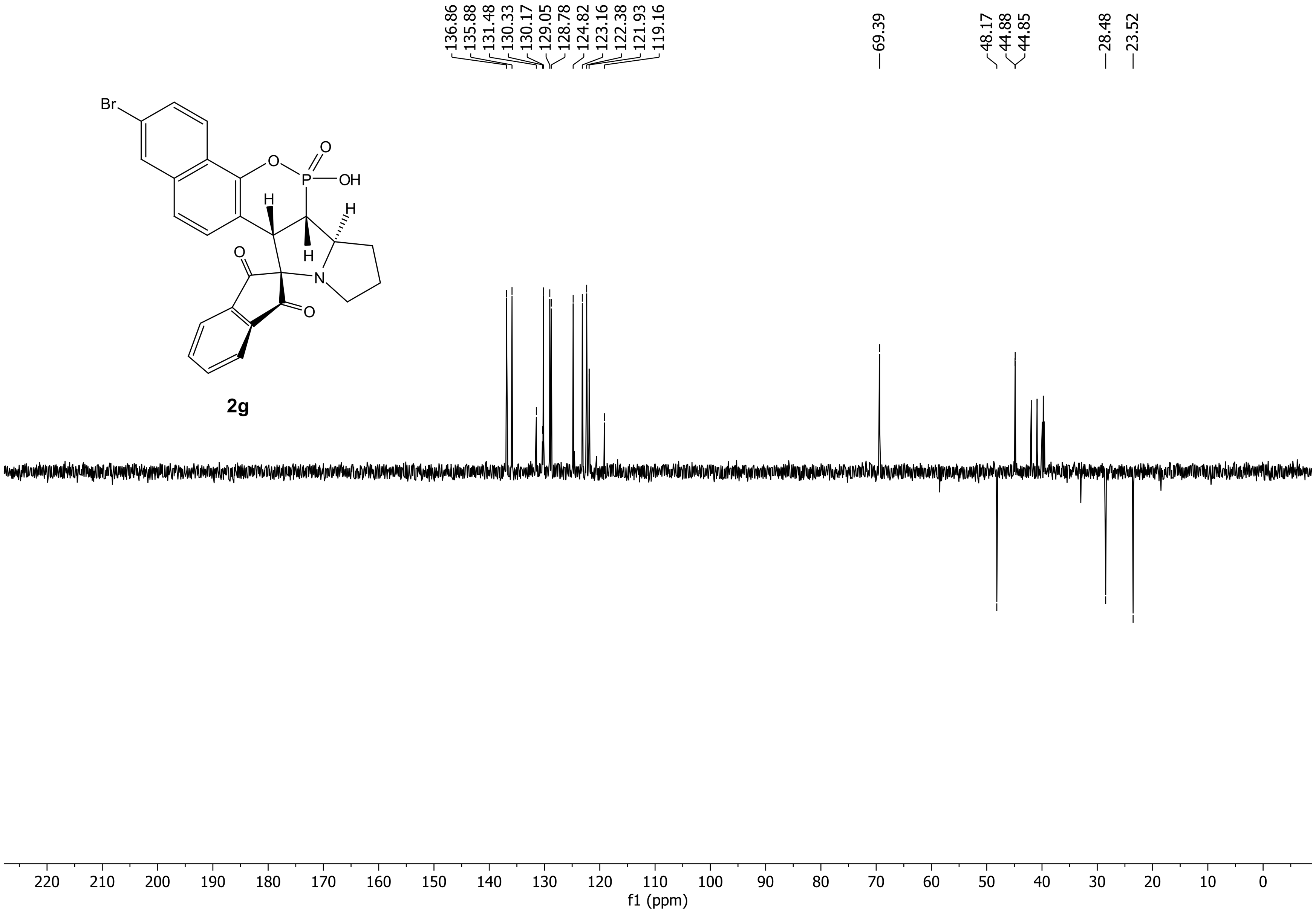

Figure S35. ^{13}C DEPT spectrum ($(CD_3)_2SO$, 151MHz) of the compound **2g**

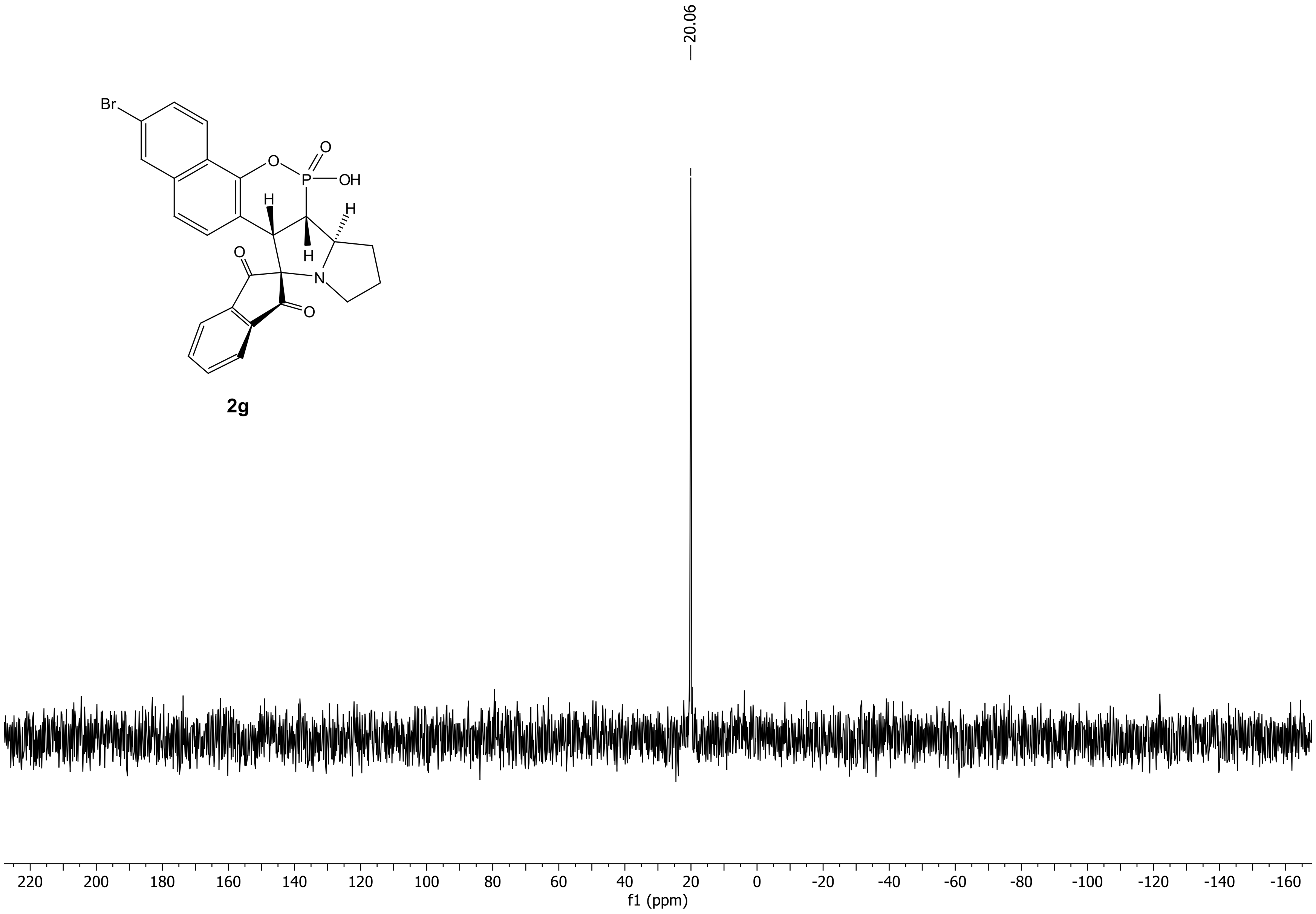

Figure S36. ^{31}P NMR spectrum ($(CD_3)_2SO$, 243MHz) of the compound **2g**
